# Distinct genomic features across cytolytic subgroups in skin melanoma

**DOI:** 10.1007/s00262-021-02918-3

**Published:** 2021-03-29

**Authors:** Constantinos Roufas, Ilias Georgakopoulos-Soares, Apostolos Zaravinos

**Affiliations:** 1 Department of Life Sciences, School of Sciences, European University Cyprus, 1516 Nicosia, Cyprus; 2 Department of Bioengineering and Therapeutic Sciences, University of California, San Francisco, CA 94158 USA; 3 Institute for Human Genetics, University of California, San Francisco, CA 94143 USA; 4 Department of Basic Medical Sciences, College of Medicine, Member of QU Health, Qatar University, 2713 Doha, Qatar

**Keywords:** Skin melanoma, Cytolytic activity, Immune checkpoints, Chromothripsis, Kataegis, Immunophenoscore

## Abstract

**Background:**

Skin melanoma is a highly immunogenic cancer. The intratumoral immune cytolytic activity (CYT) reflects the ability of cytotoxic T and NK cells to eliminate cancer cells, and is associated with improved patient survival. Despite the enthusiastic clinical results seen in advanced-stage metastatic melanoma patients treated with immune checkpoint inhibitors, a subgroup of them will later relapse and develop acquired resistance. We questioned whether CYT associates with different genomic profiles and thus, patient outcome, in skin melanoma.

**Methods:**

We explored the TCGA-SKCM dataset and stratified patients to distinct subgroups of cytolytic activity. The tumor immune contexture, somatic mutations and recurrent copy number aberrations were calculated using quanTIseq, MutSigCV and GISTIC2. Chromothriptic events were explored using CTLPScanner and cancer neoepitopes were predicted with antigen garnish. Each tumor's immunophenoscore was calculated using Immunophenogram. Mutational signatures and kataegis were explored using SigProfiler and compared to the known single or doublet base substitution signatures from COSMIC.

**Results:**

Metastatic skin melanomas had significantly higher CYT levels compared to primary tumors. We assessed enrichment for immune-related gene sets within CYT-high tumors, whereas, CYT-low tumors were enriched for non-immune related gene sets. In addition, distinct mutational and neoantigen loads, primarily composed of C > T transitions, along with specific types of copy number aberrations, characterized each cytolytic subgroup. We found a broader pattern of chromothripsis across CYT-low tumors, where chromosomal regions harboring chromothriptic events, contained a higher number of cancer genes. SBS7a/b, SBS5 and SBS1 were the most prevalent mutational signatures across both cytolytic subgroups, but SBS1 differed significantly between them. SBS7a/b was mutually exclusive with SBS5 and SBS1 in both CYT subgroups. CYT-high patients had markedly higher immunophenoscore, suggesting that they should display a clinical benefit upon treatment with immune checkpoint inhibition therapy, compared to CYT-low patients.

**Conclusions:**

Overall, our data highlight the existence of distinct genomic features across cytolytic subgroups in skin melanoma, which might affect the patients' relapse rate or their acquisition of resistance to immune checkpoint inhibition therapies.

**Supplementary Information:**

The online version contains supplementary material available at 10.1007/s00262-021-02918-3.

## Background

Cutaneous melanoma is a very aggressive and highly immunogenic cancer with extensive genetic and transcriptional diversity [1]. The tumor usually results after exposure to UV radiation, and has the highest rate of somatic mutations and neoantigens among all cancer types [2]. Recently, it was classified into mutant BRAF, mutant RAS, mutant NF1, and Triple-WT genetic subtypes, by the Cancer Genome Atlas (TGCA) Network, based on the pattern of the top mutated genes [3].

The tumor’s microenvironment comprises a very heterogeneous cell population, including fibroblasts, lymphocytes, macrophages and other immune cells, as well as, adipocytes and cells that form the structural elements of skin blood vessels dashed in the extracellular matrix [4]. A high content of tumor-infiltrating lymphocytes (TILs) in the tumor’s microenvironment associates with a favorable prognosis and improved overall survival of the patients [5, 6]. An increased immune cytolytic activity (CYT), defined by the attempt of CD8 + cytotoxic T cells and natural killer (NK) cells to kill cancer cells via the secretion of GZMA and PRF1 toxins, has also been linked with better patient survival [7].

With the advent of immunotherapies, the tumor’s management has shifted from cytokine-based treatment to immune checkpoint inhibition, primarily of cytotoxic T-lymphocyte-associated antigen-4 (CTLA-4) [8] and programmed cell-death protein 1 (PD-1) or its ligands (PD-L1 and PD-L2) [9]. Monoclonal antibodies including Ipilimumab, Tremelimumab (anti-CTLA-4), Pembrolizumab, Nivolumab, Cemiplimab (anti-PD-1), Atezolizumab, Avelumab and Durvalumab (anti-PD-L1) among others, including lymphocyte activation gene-3 (LAG-3, CD223) which is currently on clinical trial [10], have enthusiastically provided long-lasting responses and significantly improved the overall survival in patients with metastatic skin melanoma.

Early clinical results have also been stated with IDO inhibitors [11] and Treg depletion [12]. Other immunotherapeutic agents are also under study, targeting 4-1BB, Ox40, Inducible T-cell COStimulator (ICOS), CD40, B7-H3, B7-H4, Tim3 and killer inhibitory receptors (KIRs); as well as cytokines IL-7, IL-15 and IL-21.

Due to the complexity of immune regulation in vivo, combination immunotherapies are expected to provide a better therapeutic outcome for the patients. Nevertheless, a subgroup of responders will later relapse and develop acquired resistance; whereas, others do not respond at all to immunotherapy, due to primary resistance [13]. Therefore, the elucidation of the underlying mechanisms of such a resistance is emergently needed, but the intratumoral immune cytolytic activity has not been previously considered in this context.

Here, we questioned whether CYT associates with different genomic profiles in skin melanoma. In specific, we investigated how the immune “cytolytic” landscape in these (primary and metastatic) tumors relates to different types of somatic mutations, mutational signatures, copy number aberrations and chromothriptic events; as well as with the expression of immune checkpoints (or other immune-related markers) and the presence of different types of immune cells within their tumor microenvironment. We also predicted how patients with different levels of immune cytolytic activity are expected to respond to single or combined immune checkpoint blockade. Overall, we provide enough evidence of the existence of distinct genomic features across immune cytolytic subgroups in skin melanoma patients, which could significantly affect their resistance to immune checkpoint inhibition therapies.

## Methods

### Skin melanoma (SKCM) data extraction

We extracted the clinical information, Mutation Annotation files (MAF) and mRNA-Seq ‘level 3′ data of a total of 470 skin melanomas (103 primary tumors, 68 distant metastasis, 74 regional cutaneous or subcutaneous tissues and 222 regional lymph nodes), along with their matched peripheral blood, from the Cancer Genome Atlas’ (TCGA-SKCM dataset) GDC Data Portal (https://portal.gdc.cancer.gov/). Three samples were non-informative and were thus, excluded from subsequent analyses. In addition, we accessed each patient’s gene-level, zero-centered, focal copy-number data analyzed by GISTIC (v2.0.22) from Broad GDAC Firehose (https://gdac.broadinstitute.org/).

### Cytolytic activity calculation and downstream RNA-seq analysis

We calculated each SKCM patient’s levels of immune cytolytic activity (CYT), as the geometric mean of the expression of *GZMA* and *PRF1* [7, 14, 15]. GZMA is one among five different types of granzymes, i.e., serine proteases that cytotoxic T lymphocytes (CTL) and natural killer (NK) cells use to kill their target cells via caspase-independent apoptosis; while PRF1 forms pores in the surface of the target cells facilitating the entry of granzymes into them [16]. Gene expression values were presented in Transcripts Per Million (TPM).

We then divided patients into two cohorts, the upper 25th quartile of the cytolytic index (CYT-high) and the lower 25th quartile (CYT-low), each with an identical admixture of histology-stage combinations. In all subsequent evaluations we compared between CYT-high and CYT-low (metastatic or primary) skin melanomas. P-values were adjusted using the Benjamini-Hochberg (BH) method.

To analyze RNA-seq data, we first mapped the reads to the human reference genome (GRCh37/hg19) and summarized them to the gene level using *Rsubread*. This produced a matrix of counts, which were then converted to log_2_-counts-per-million (logCPM). We estimated the mean–variance relationship of the log-counts using voom. *Limma* was used to detect the significantly differentially expressed genes between the two cytolytic subsets in skin melanoma (primary and metastatic) vs the normal skin tissue. We considered as differentially expressed genes, those having a BH-adjusted *p*-value < 0.1.

We used gene set variation analysis (GSVA) to refine alterations in pathway activity and clustered tumors hierarchically, with complete linkage as the distance metric. Graphs were plotted with ggplot2.

Immunohistochemistry (IHC) data were extracted from the Human Protein Atlas [17] (http://www.proteinatlas.org), and further analyzed. The antibodies used in IHC and corresponding method of retrieval, were as follows: rabbit pAb anti-GZMA, HPA054134, 1:200 dilution, Sigma-Aldrich, Atlas Antibodies Cat#HPA054134, RRID:AB_2682395; Antigen retrieval was performed using HIER pH6; and mouse mAb anti-PRF1, CAB002436, 1:10 dilution, Leica Biosystems, Cat#NCL-PERFORIN, RRID:AB_563955; Antigen retrieval was performed using HIER pH6.

### Cell type fractions and heterogeneity

We quantified the tumor immune contexture from the RNA-seq data, using quanTIseq (https://icbi.i-med.ac.at/software/quantiseq/doc/). Additionally, we used CIBERSORT to identify fractions of immune subpopulations in each cytolytic subgroup’s tissue (https://cibersort.stanford.edu/). Tumor purity and ploidy estimates were produced using ABSOLUTE and plotted for each cytolytic subgroup of primary and metastatic tumors.

### Detection of somatic mutations and copy number aberrations (SCNA)

We calculated the most significantly mutated genes (SMG) in each CYT subtype of primary and metastatic skin melanomas, using MutSigCV (v1.3.01) with an FDR (*q*-value) equal to 0.1 as threshold of significance. We further employed GISTIC (v2.0.22) to identify genomic regions with significant amplifications or deletions in each sample. A G-score considering the amplitude of the aberration, as well as the frequency of its occurrence across each tumor sample was assigned. We calculated the SCNA in each sample by keeping the sum of segment mean changes ≥ 0.6 and ≤ − 0.4 between somatic and normal samples. Regions with FDR q-values < 0.1 were considered significant. Both mutational and copy number analyses were performed using the GenePattern platform from the Broad Institute (http://software.broadinstitute.org/cancer/software/genepattern).

### Detection of chromothriptic events

Chromothriptic events in CYT-high or low skin melanomas were investigated using CTLPScanner. In brief, after downloading segmentation data (level 3) of single nucleotide polymorphism (SNP) arrays from the TCGA-SKCM dataset, we implemented DNA copy number segmentation data, using the circular binary segmentation algorithm and the NCBI38/hg38 genome assembly. For the detection of chomothripsis or chomothripsis-like regions, we used the default parameters of copy number status change ≥ 20 times, log10 of likelihood ratio ≥ 8, minimum segment size = 10 kb, and signal distance between adjacent segments = 0.3. Shattered chromosomomal regions were visualized based on the signal value for genomic gains (≥ 0.15) or losses (≤ -0.15) and further highlighted. Finally, chromothripsis-located genes were annotated using the COSMIC database. Histograms of copy number segment switches were drawn to indicate the emergence of the major genomic rearrangements.

### Detection of cancer neoepitopes and immunophenoscores

We predicted the cancer neoepitopes using *antigen.garnish*, with the following MHC molecules, as previously described [14]: H-2-IAb, H-2-IAd, HLA-DPA1*01:03, HLA-DPA1*02:01, HLA-DPA1*02:01, HLA-DPA1*03:01, HLA-DPB1*03:01, HLA-DQA1*01:01, HLA-DQA1*01:02, HLA-DQA1*03:01, HLA-DQA1*04:01, HLA-DQA1*05:01, HLA-DQA1*05:01, HLA-DRB1*01:01. We classified the predicted cancer neoepitopes as classically (CDNs) or alternatively defined (ADNs).

The Cancer Immunome Database (TCIA) [18] (https://tcia.at/) was also queried for cellular composition of neoantigens among the two cytolytic subsets of skin melanomas. The immunophenoscore (IPS) of patients within each cytolytic subgroup was calculated using *Immunophenogram* and immunophenograms were constructed to visualize the immunophenotypes of each melanoma sample. The IPS ranged from 0 to 10, according to the expression of MHC molecules, immunomodulators, effector cells [activated CD8 + and CD4 + T cells, effector memory (Tem) CD8 + and CD4 + cells] and suppressor cells [regulatory T cells (Tregs) and myeloid-derived suppressor cells (MDSCs)].

### Mutational signatures and kataegis

We extracted mutational signatures as previously described [19] and analyzed them using SigProfiler’s MatrixGenerator and Extractor bioinformatic tools in Python with the default parameters. We focused on single base substitutions (SBS) and doublet base substitution (DBS) signatures. We then compared these signatures against the known SBS and DBS signatures from the Catalog of Somatic Mutations in Cancer [COSMIC v3.1, release v91 (June 2020)]. We further applied cosine similarity to identify the best matches within signatures. We calculated each signature’s contribution within each cytolytic subgroup and estimated statistical significance with the Mann–Whitney U test. All p-values were Bonferonni-corrected.

The existence of localized hyper-mutations, or kataegis, is a specific mutation pattern caused by APOBEC enzymes which was recently found to correlate independently with the expression of PD-L1/PD-L2 in melanoma [20]. We defined as kataegistic regions within each cytolytic subgroup in skin melanoma, those that contained ≥ 6 consecutive mutations with an average inter-mutation distance of ≤ 1000 bp [19].

### Tumor genomic heterogeneity

We inferred tumor genomic heterogeneity in SKCM tumors by clustering the variant allele frequencies (VAF). We quantitatively calculated the magnitude of each skin melanoma’s intra-tumoral heterogeneity by the width of its corresponding VAF distribution. We also assigned a mutant-allele tumor heterogeneity (MATH) score to each skin melanoma sample.

### Patient survival and synergistic target analysis

The overall survival of the melanoma patients was performed using data extracted from the TCGA-SKCM dataset. We used Kaplan–Meier curves with the log rank (Mantel Cox) test to estimate patient survival. We also examined whether *GZMA* and *PRF1* act synergistically on patient survival, using SynTarget [21].

## Results

### Immune cytolytic activity in cutaneous melanoma

**Fig. 1:**
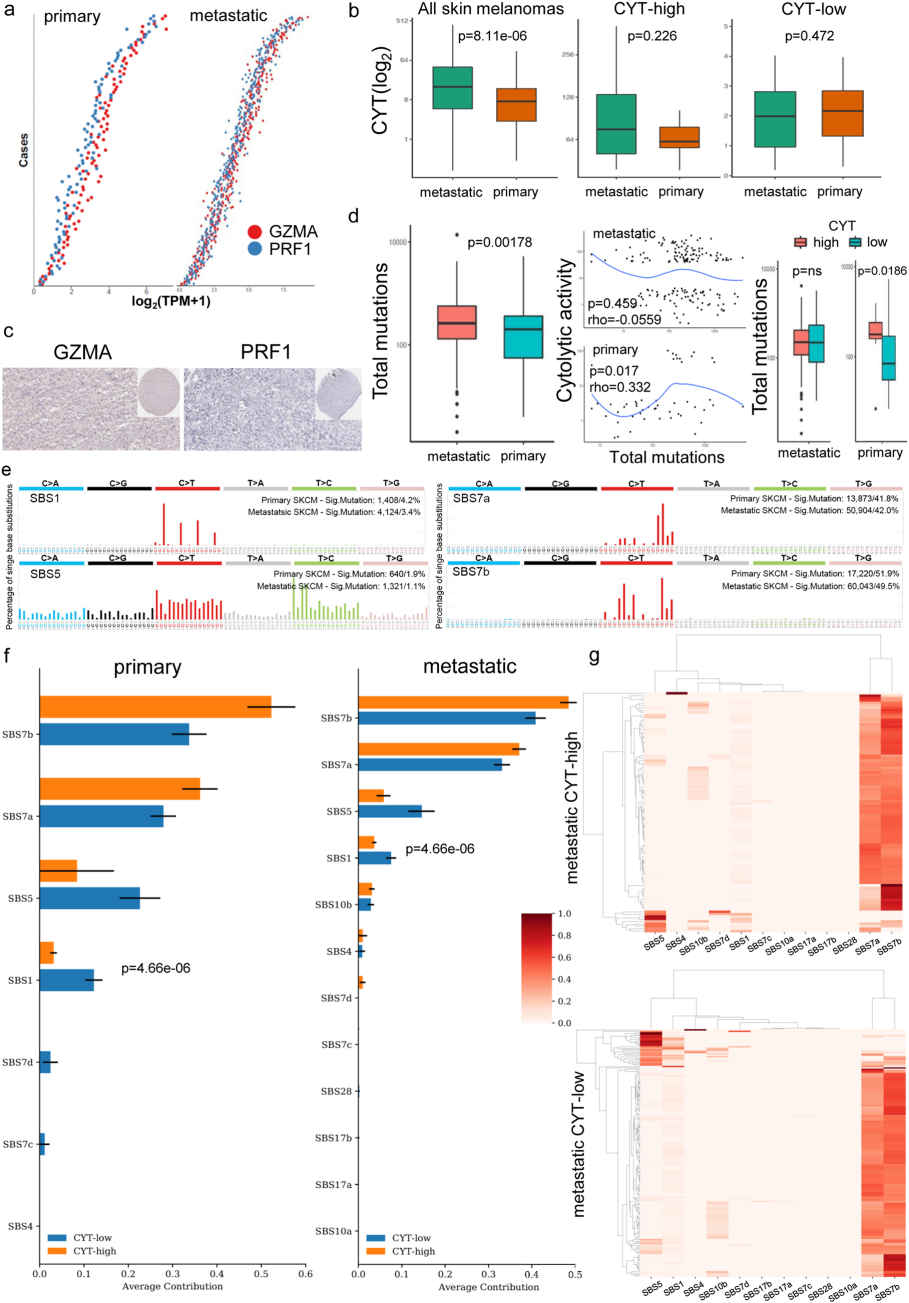
Immune cytolytic activity in cutaneous melanoma. **a** Distribution of the GZMA and PRF1 genes across primary and metastatic skin melanomas. GSVA signature scores for CYT discriminated the upper quartile (CYT-high) from the bottom quartile (CYT-low) tumors. **b** Cytolytic activity (CYT(log_2_)) across all melanoma samples in the SKCM dataset, as well as among the two cytolytic subgroups of melanomas. **c** GZMA and PRF1 protein expression in tissue microarrays (TMA) of skin melanoma, derived from the Human Protein Atlas. In the SKCM dataset, the GZMA protein levels were low in 6 skin melanomas and absent in 5/12 analyzed skin melanoma samples. PRF1 protein was absent in all the skin melanoma samples. **d** Total mutation count in metastatic and primary melanomas. The local regression curves in the middle depict the (Spearman rank) correlation between CYT and the total mutation count. Boxplots in the right show the distributions between the two cytolytic groups in metastatic and primary melanomas (Mann–Whitney). **e** Mutational profiles in primary and metastatic skin melanomas, using the conventional 96 mutation type classification, which is based on the 6 substitution subtypes: C > A, C > G, C > T, T > A, T > C, and T > G. Further, each of the substitutions is examined by incorporating information on the bases immediately 5′ and 3′ to each mutated base generating 96 possible mutation types (6 types of substitution × 4 types of 5′ base × 4 types of 3′ base). **f** Average contribution of each single base substitution (SBS) mutational signature in CYT-high and CYT-low (primary and metastatic) skin melanomas. The most prevalent mutational signatures were SBS7a/b, SBS5 and SBS1. Differences between CYT-high and CYT-low were calculated using Mann–Whitney U test with Bonferroni correction. **g** Two-way hierarchical clustering of the SBS signatures in CYT-high and CYT-low metastatic skin melanoma

We assessed the intratumoral immune cytolytic activity in skin melanoma patients, measuring the TPM values of their *GZMA* and *PRF1* [7]. Table S1 lists the exact TCGA-SKCM dataset samples that were included in our analysis, along with their clinical information. We then stratified patients by defining skin melanomas in the upper quartile of the cytolytic index, as CYT-high, and those in the lower quartile, as CYT-low. GZMA and PRF1 were tightly co-expressed across both primary and metastatic tumors (Spearman rank correlation, rho ~ 0.9) (Fig. 1a).

We and others have previously shown that CYT is markedly higher in skin melanoma relative to the normal skin [7, 15]. Here, we found that metastatic melanomas showed significantly higher cytolytic activity compared to primary tumors (*p* = 8.11e-06). Importantly, this was not observed between primary and metastatic tumors falling within the same extreme CYT percentiles, suggesting the existence of two main subgroups in these tumors (Fig. 1b).

At the protein level, it was evident that neither toxin was highly expressed in these tumors, probably reflecting their short half-lives. In specific, GZMA protein expression (nuclear or cytoplasmic/membranous) was medium only in one skin melanoma; whereas, low in six and absent in five out of twelve skin tumors. The corresponding intensity scores were moderate (2 out of 12 tumors), weak (7 out of 12 tumors) and negative (3 out of 12 tumors). The absence of PRF1 protein was more evident, as it was not detected in any of the 11 tumor samples (Fig. 1c and Table S2).

As expected, metastatic melanomas had a significantly higher mutational load compared to primary tumors (*p* = 0.00178) (Fig. 1d). However, when CYT-high and CYT-low tumors were assessed separately, the mutation load did not differ within metastatic tumors (*p* = 0.772) and did not correlate with the cytolytic index. In contrast, CYT-high primary tumors had a higher mutational burden (*p* = 0.0186), which was significantly correlated with the CYT index (Fig. 1d). These data suggest a primary association between cytolytic activity and mutation load in primary skin melanoma.

In tune with the mutagenic role of UV radiation in melanoma, the most prevalent mutational signatures that we detected had higher cosine-similarity with COSMIC's signatures SBS7a/SBS7b, SBS5 and SBS1 (Fig. 1e).

SBS7a and SBS7b are both associated with DBS1, which exhibits predominantly CC > TT mutations and with the small Insertion and Deletion (ID) Signature 13 (ID13), also characterized by single base T deletions at TT dinucleotides and found in cancers of the skin from sun exposed areas. The SBS7a/b signatures also exhibit transcriptional strand bias with more mutated C than G bases on untranscribed strands of genes compatible with damage to C and activity of transcription-coupled nucleotide excision repair (TC-NER).

SBS5 and SBS1 are both very similar (cosine similarity = 0.96) clock-like signatures, in that the number of their mutations correlates with aging. SBS1 is due to deamination of 5-methylC to T which generates G:T mismatches in double stranded DNA. Failure to detect and remove these mismatches prior to DNA replication results in fixation of the T substitution for C. SBS5 also shows transcriptional strand bias for T > C substitutions at ATN context with more mutated A than T bases on the untranscribed strands of genes compatible with damage to A and activity of TC-NER.

Interestingly, the SBS7a/b signature was mutually exclusive with SBS1 and SBS5 in both cytolytic subgroups in metastatic tumors (Fig. 1e).

### Kataegis is equally distributed across different cytolytic subgroups of skin melanoma

We hypothesized that mutation showers (or kataegis) are associated with cytolytic-high cutaneous melanomas. Kataegis were recently uncovered by whole-genome sequencing of B cell and non-hematopoietic tumors [19, 22–25]. We identified 74 kataegic sites across 26 tumors, associated with 1,567 mutations. Five (19.2%) of these tumors were CYT-high metastatic SKCMs and six (23.07%) CYT-low (3 metastatic and 3 primary tumors), suggesting that kataegis is equally distributed across skin tumors of different cytolytic activity.

Importantly, 1,395 of the mutations (89%) were C > T transitions, which is consistent with the notion that kataegis results from DNA replication over cytidine deamination of resected DNA [25, 26]. Considering the established role of the APOBEC cytidine deaminases in kataegis [20], these data support a partial APOBEC involvement in cutaneous melanomas, irrespective of their immune cytolytic index.

We next investigated whether CYT associates with transcriptional or genomic aberrations in cytolytic subgroups in primary and metastatic skin melanomas.

### CYT correlates with distinct gene sets in melanoma

We initially identified the top deregulated genes in skin melanoma (primary and metastatic) versus the normal skin tissue (Figure S2). Using principal-components analysis (PCA), it was evident that the differentially expressed genes in metastatic skin melanomas could discriminate them better from the normal skin, compared to those in primary tumors. We detected 1,054 (11.5%) co-deregulated genes (adj. *p* < 0.001) between primary and metastatic skin melanomas, but also 13 (0.1%) genes that were deregulated only in primary and 8,121 (88.4%) genes in metastatic skin melanomas (Table S3). Importantly, *PRF1, GZMA, NKG7, SLA2, GBP5 and CD2* were among the top 20 upregulated genes both in primary and metastatic skin melanomas. In primary SKCM the top 20 deregulated genes also included *CD8A, LAG3, PDCD1, ITGAL, TRAC, TIGIT, TNFRSF9, CD247, CD27, SIT1, CD8B, CCR5, SIRPG* and *CD3D* (Table S3 and Figure S3). On the other hand, the top 20 upregulated genes among metastatic tumors also included *IRF1, CD74, FASLG, JAKMIP1, C1QA, IFNG, TNIP3, C1QB, APOL3, C1QC, CCL5, TRGV10, CRTAM* and *CD8A* (Table S3 and Figure S3).

To better evaluate the sources of gene expression across all SKCM tumors, we calculated tumor purity and ploidy, using ABSOLUTE [27]. Overall, CYT-low tumors had higher purity compared to the CYT-high ones; whereas, the ploidy values of most tumors clustered around 2.4–2.7 (genome-wide duplication), without differences between the two cytolytic subsets (Figure S4).

**Fig. 2:**
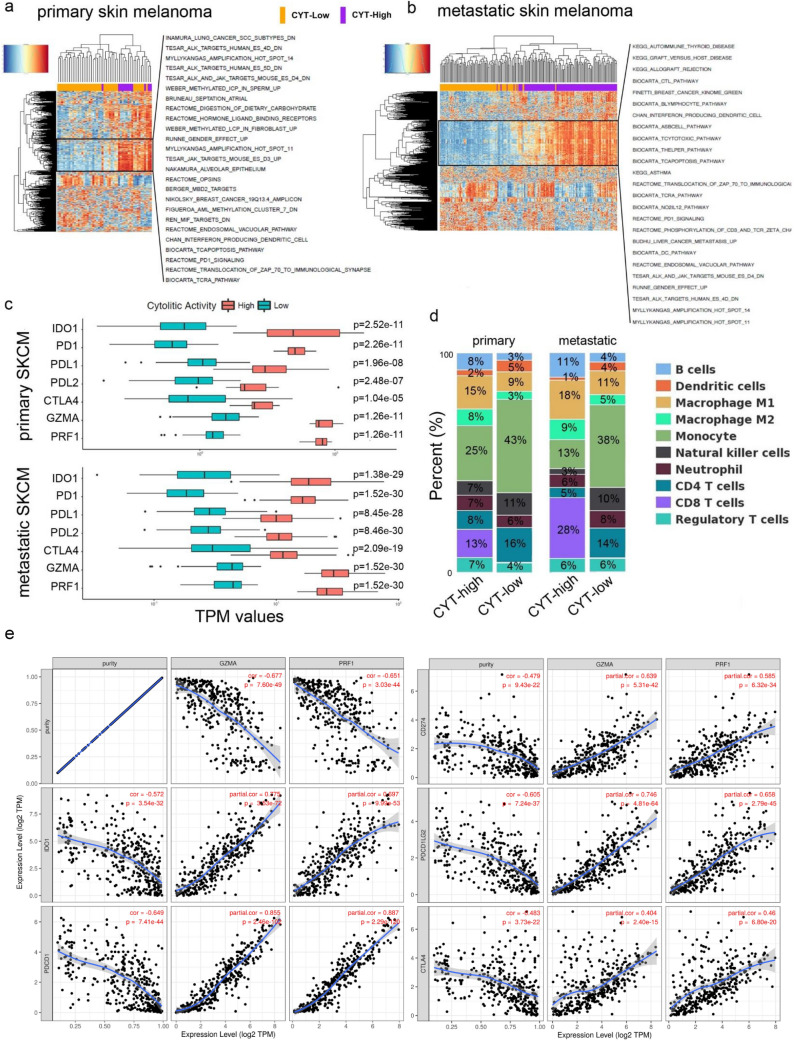
CYT correlates with distinct gene sets in melanoma. GSVA analysis in primary **a** and metastatic skin melanoma **b** revealed enrichment of gene sets containing markers for dendritic cell activation and T-cell inhibition in CYT-high skin tumors. **c** Other than GZMA and PRF1, the immune checkpoint molecules IDO1, PD1, PD-L1, PD-L2, and CTLA-4 were significantly overexpressed in cytolytic-high (primary and metastatic) SKCM tumors. **d** Cell type fractions within each cytolytic subgroup of primary and metastatic SKCM. CYT-high tumors are enriched in B cells, M1 macrophages and CD8 + T cells; whereas, CYT-low tumors contain significantly higher levels of monocytes, NK cells and CD4 + T cells. **e** Scatterplot depicting the significant correlation between IDO1, PD-1, PD-L1, PD-L2 and CTLA-4 and the two cytolytic genes (GZM1 and PRF1) in SKCM. The correlation was adjusted by tumor purity. The Spearman’s rho value and estimated statistical significance are provided in red

Using GSVA analysis and the “C2: curated gene sets” from MSigDB v6.1 (containing 4,738 gene sets) [28], we assessed enrichment within the CYT-high primary skin melanomas, for the immune-related gene sets BIOCARTA_TCRA_PATHWAY, REACTOME_TRANSLOCATION_OF_ZAP_70_TO_IMMUNOLOGICAL_SYNAPSE, REACTOME_PD1_SIGNALING, BIOCARTA_TCAPOPTOSIS_PATHWAY, CHAN_INTERFERON_PRODUCING_DENDRITIC_CELL and REACTOME_ENDOSOMAL_VACUOLAR_PATHWAY. On the other hand, CYT-low primary tumors were statistically enriched for DNA replication and DNA-repair gene sets, including REACTOME_REPAIR_SYNTHESIS_FOR_GAP_FILLING_BY_DNA_POL_IN_TC_NER, REACTOME_POL_SWITCHING, REACTOME_LAGGING_STRAND_SYNTHESIS, REACTOME_PROCESSIVE_SYNTHESIS_ON_THE_LAGGING_STRAND, REACTOME_DNA_STRAND_ELONGATION, REACTOME_REMOVAL_OF_THE_FLAP_INTERMEDIATE_FROM_THE_C_STRAN, REACTOME_UNWINDING_OF_DNA, BIOCARTA_MCM_PATHWAY, REACTOME_ACTIVATION_OF_THE_PRE_REPLICATIVE_COMPLEX, KALMA_E2F1_TARGETS, CROSBY_E2F4_TARGETS, among others (Fig. 2).

CYT-high metastatic melanomas were also enriched for immune-related gene sets, including KEGG_AUTOIMMUNE_THYROID_DISEASE, KEGG_GRAFT_VERSUS_HOST_DISEASE, KEGG_ALLOGRAFT_REJECTION, BIOCARTA_CTL_PATHWAY, BIOCARTA_TCYTOTOXIC_PATHWAY, BIOCARTA_THELPER_PATHWAY, BIOCARTA_THELPER_PATHWAY, BIOCARTA_TCAPOPTOSIS_PATHWAY, BIOCARTA_TCRA_PATHWAY, REACTOME_PD1_SIGNALING, and BIOCARTA_DC_PATHWAY (Fig. 2); whereas, CYT-low metastatic tumors were enriched for non-immune-related gene sets (TESAR_ALK_TARGETS_HUMAN, MYLLYKANGAS_AMPLIFICATION, R_KINOME_KUMAMOTO_RESPONSE_TO_NUTLIN_MONTERO_THYROID_CANCER_POOR_SEMBA_FHIT_TARGETS_DN, CROSBY_E2F4_TARGETS.

REACTOME_UNWINDING_OF_DNA, ZERBINI_RESPONSE_TO_SULINDAC_OHASHI_AURKA_TARGETS, etc.).

Interferon-gamma (IFN-γ) is an important cytokine produced by activated T cells, NK and NK T cells in the tumor microenvironment, where it orchestrates innate and adaptive antitumor immune responses [29]. To gain further insight into the relationship of IFN-γ and the cytolytic index in skin melanoma, we explored two IFN-γ signatures that were recently proposed to predict patient response to Pembrolizumab (anti-PD-1), the “*IFN-γ*” and the “*expanded immune*” signatures [30]. These signatures contain IFN-γ–responsive genes related to antigen presentation, chemokine expression, cytotoxic activity and adaptive immune resistance (“*IFN-γ*” signature: *IFNγ, IDO1, CXCL9, CXCL10, HLA-DRA, STAT1*; and “*expanded immune*” signature: *CD30, IDO1, CIITA, CD3E, CCL5, GZMK, CD2, HLA-DRA, CXCL13, IL2RG, NKG7, HLA-E, CXCR6, LAG3, TAGAP, CXCL10, STAT1, GZMB*). Both gene signatures were markedly overexpressed in CYT-high tumors, corroborating the enrichment that we found for the similar gene set CHAN_INTERFERON_PRODUCING_DENDRITIC_CELL across these tumors (Figure S5) and their high discriminatory value in enriching response rates to Pembrolizumab [30].

Taken together, the above results provide further proof that the tumor microenvironment in CYT-high skin melanomas is more inflamed and immunogenic compared to that in CYT-low tumors.

Other than GZMA and PRF1, the immune checkpoint genes *CTLA-4, PDCD1, CD274, PDCD1LG2* and *IDO1* were considerably overexpressed in CYT-high (primary and metastatic) SKCM tumors, underlying their immunosuppressive microenvironment (Fig. 2c).

To assess the relationship between CYT markers and immune checkpoint molecules, we run correlation analysis of their gene expression levels. Both cytolytic genes were notably correlated with the expression of several inhibitory checkpoints in SKCM tumors (*p* < 0.0001), corroborating that combining immune checkpoint inhibitors should effectively be used to overcome resistance and augment the clinical benefit for these patients (Fig. 2e).

In addition, we examined the expression of the immunoinhibitor LAG3, the immunostimulators CD70, lipoteichoic acid (LTA), ecto-5′-nucleotidase (NT5E) and ectonucleoside triphosphate diphosphohydrolase 1 (ENTPD1), as well as several regulatory cytokines and chemokines, known for their pro- and anti-inflammatory roles within the tumor microenvironment [7, 14]. The expression of these molecules was also compared between the two cytolytic subgroups in primary and metastatic SKCM. In specific, we focused on markers for activated CD8 + T cells (NKG7, CD3E, GZMA, GZMH, GZMK), MDSCs (CD2), activated dendritic cells (UBD, C1QB, C1QC), NK cells (FASLG and FAS), and IFN-stimulated chemokines that attract T cells (CXCL9, CXCL10, CXCL11 and CXCL13). All these genes were significantly overexpressed in CYT-high (primary and metastatic) melanomas, supporting the existence of an inflamed microenvironment in them (Figure S6).

Cell type fraction analysis revealed that CYT-high (primary and metastatic) tumors are significantly enriched in B cells, M1 macrophages and CD8 + T cells; whereas CYT-low tumors contain significantly higher levels of monocytes, NK cells and CD4 + T cells (Fig. 2d). Further CIBERSORT analysis revealed that the majority of (primary and metastatic) CYT-high tumors were significantly enriched in γδT cells, follicular helper T cells (Tfh) and Tregs, compared to their CYT-low counterparts (**Figure S7**). These cells were previously shown to participate in cancer immunosurveillance [31], autoimmunity [32] and immunosuppression [33].

### CYT correlates with discrete mutational events in skin melanoma

**Fig. 3:**
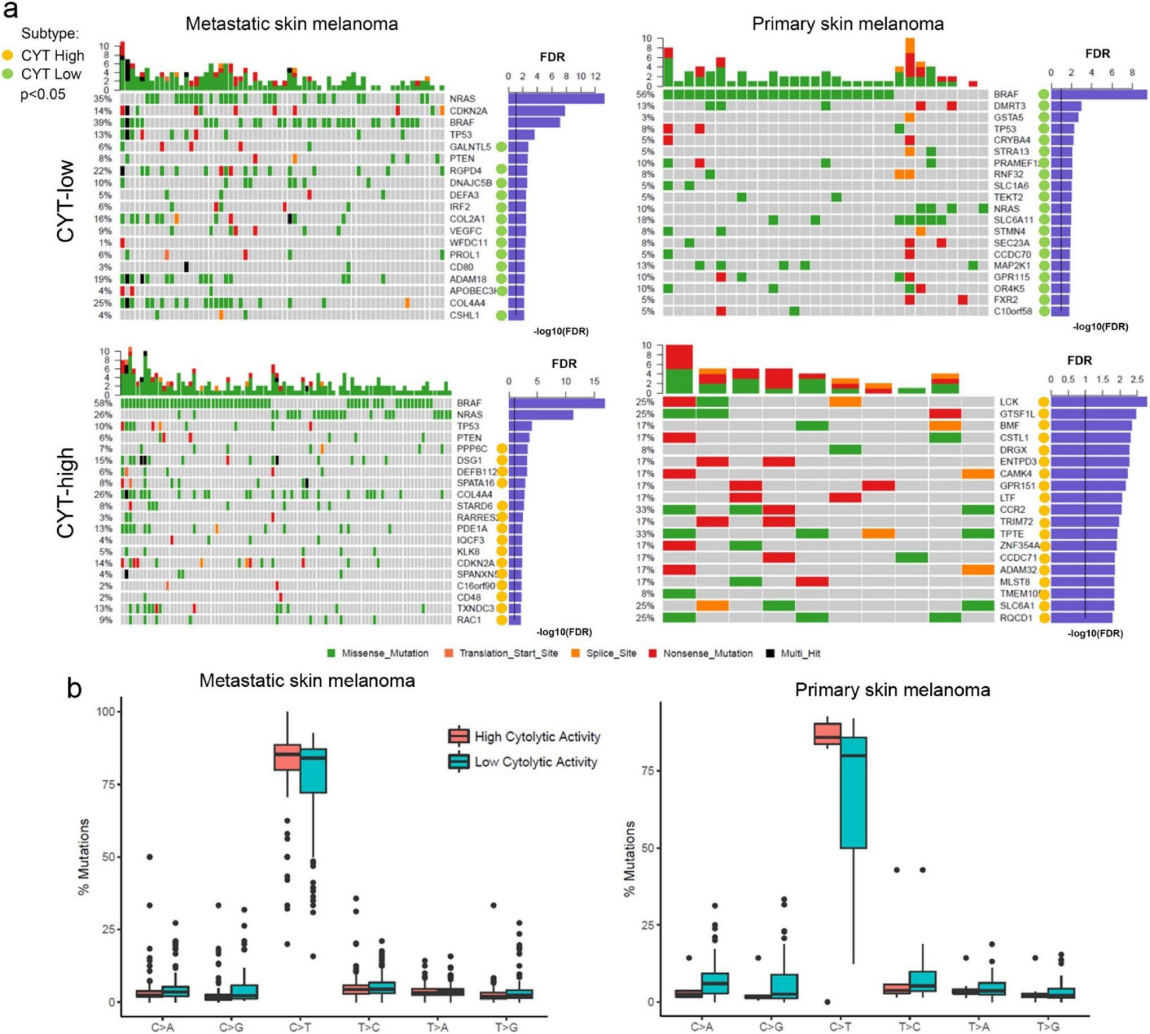
CYT correlates with discrete mutational events in melanoma. **a** The co-mutation plots depict the significantly mutated genes (SMGs, FDR < 0.1) in CYT-high and CYT-low metastatic (left) and primary (right) skin melanomas. Green, red, orange and black boxes indicate missense, nonsense, splice site and multi-hit mutations, respectively. The SMGs that correlate with the two cytolytic subtypes (*p* < 0.05) are marked with green (CYT-low) or orange (CYT-high) circles next to each gene’s name. The FDR p-values for SMGs are plotted in -log10 on the right side of the plots. **b** Nonsynonymous mutation spectra across the two cytolytic subgroups in primary and metastatic skin melanomas

We next focused on the exome-seq data of the TCGA-SKCM and detected the significantly mutated genes in each cytolytic subgroup of skin melanoma. CYT-low primary tumors were significantly associated with mutations in *BRAF, DMRT3, GSTA5, TP53, CRYBA4, STRA13, PRAMEF12, RNF32, SLC1A6, TEKT2* and *NRAS* among others; whereas, CYT-high primary tumors with a totally different group of genes, including *LCK, GSTSF1L, BMF, CSTL1, DRGX, ENTPD3, CAMK4, GPR151, LTF* and others (Fig. 3a). This discrepancy hints that the two cytolytic subsets correlate with different somatic mutations; but we should also take into consideration the small sample number of CYT-high primary tumors (*n* = 10) compared to the CYT-low ones (*n* = 31).

In contrast, the SMGs in both cytolytic subgroups of metastatic melanomas included previously described [34] driver mutations in oncogenes (*NRAS, BRAF* and *CDKN2A*) and tumor suppressors (*TP53* and *PTEN*), as well as *COL4A4*.

CYT-low metastatic melanomas were associated with non-silent mutations in *GALNTL5, RGPD4, DNAJC5B, DEFA3, IRF2, COL2A1, VEGFC, WFDC11, PROL1, CD80, ADAM18, APOBEC3H* and *CSHL1*; whereas CYT-high metastatic skin tumors were significantly mutated in *PPP6C, DSG1, DEFB112, SPATA16, STARD6, RARRES2 PDE1A, IQCF3, KLK8, CDKN2A, SPANXN5, C16orf90, CD48, TXNDC3* and *RAC1* (Fig. 3a).

Although the frequency of specific substitutions did not differ between the two cytolytic subgroups in metastatic melanoma, CYT-high tumors had more C > T transitions compared to CYT-low ones (*p* < 0.01) in primary SKCM. Nevertheless, this could probably be due to the difference in sample number (103 primary vs 368 metastatic skin melanomas) (Fig. 3b).

Likewise, there was no association between CYT and *BRAF, NRAS, TP53* or *NF1* mutations, indicating that immune-related alterations within the tumor microenvironment, and therefore immunotherapies, are independent of the tumor’s genotype (Figure S8).

### CYT associates with different structural changes in skin melanoma

Skin melanoma is characterized by increased chromosomal instability (CIN) with extensive gains and losses, which associate with poor patient prognosis [35]. However, it is unknown whether these chromosomal aberrations correlate with the patients' immune cytolytic index. Therefore, we assessed the somatic copy number aberrations (SCNA) within each cytolytic subtype in primary and metastatic skin melanomas.

CYT-low primary tumors had recurrent amplification in locus 1p12 (NOTCH2) and deletions in loci 9p22.4 (JAK2, PDL1/CD274, PDL2/PDCD1LG2), 10q23.1 (miR346), 11q25 (ETS1), 15q15.1 (RAD51), 16q23.3 (CDH13) and 6q27 (CCR6, RNASET2, FGFR1OP). On the other hand CYT-low metastatic melanomas had recurrent amplifications in loci 5p15.33 (TERT), 6p25.1 (CDYL), 7p22.2 (RAC1), 12q15 (MDM1) and recurrent deletions in 5q31.2 (CTNNA1, FGF1), 11q22.3 (FDX1, RDX, ZC3H12C), and 15q14 (RASGRP1, CSNK1A1P1, SPRED1).

CYT-high primary melanomas on the other hand, did not have recurrent copy number amplifications above the threshold (Q value = 0.25), but rather had loses at 9p21.3 (CDKN2A/B), 9q34.11 (CDK9) and 16q24.2 (IRF8). CYT-high metastatic melanomas had recurrent copy number amplifications in loci 1p12 (NOTCH2), 1q44 (OR2M4), 7q36.1 (KCNH2), 11q13.3 (FGF3/4), 12p11.23 (MED21) and 12q13.3 (R3HDM2) and deletions in 1p22.1 (RHOC, NRAS), 9p21.3 (CDKN2B), 10q23.2 (miR346), 11q23.3 (ATM, CHEK1, ETS1) and 15q15.3 (CASC4).

**Fig. 4:**
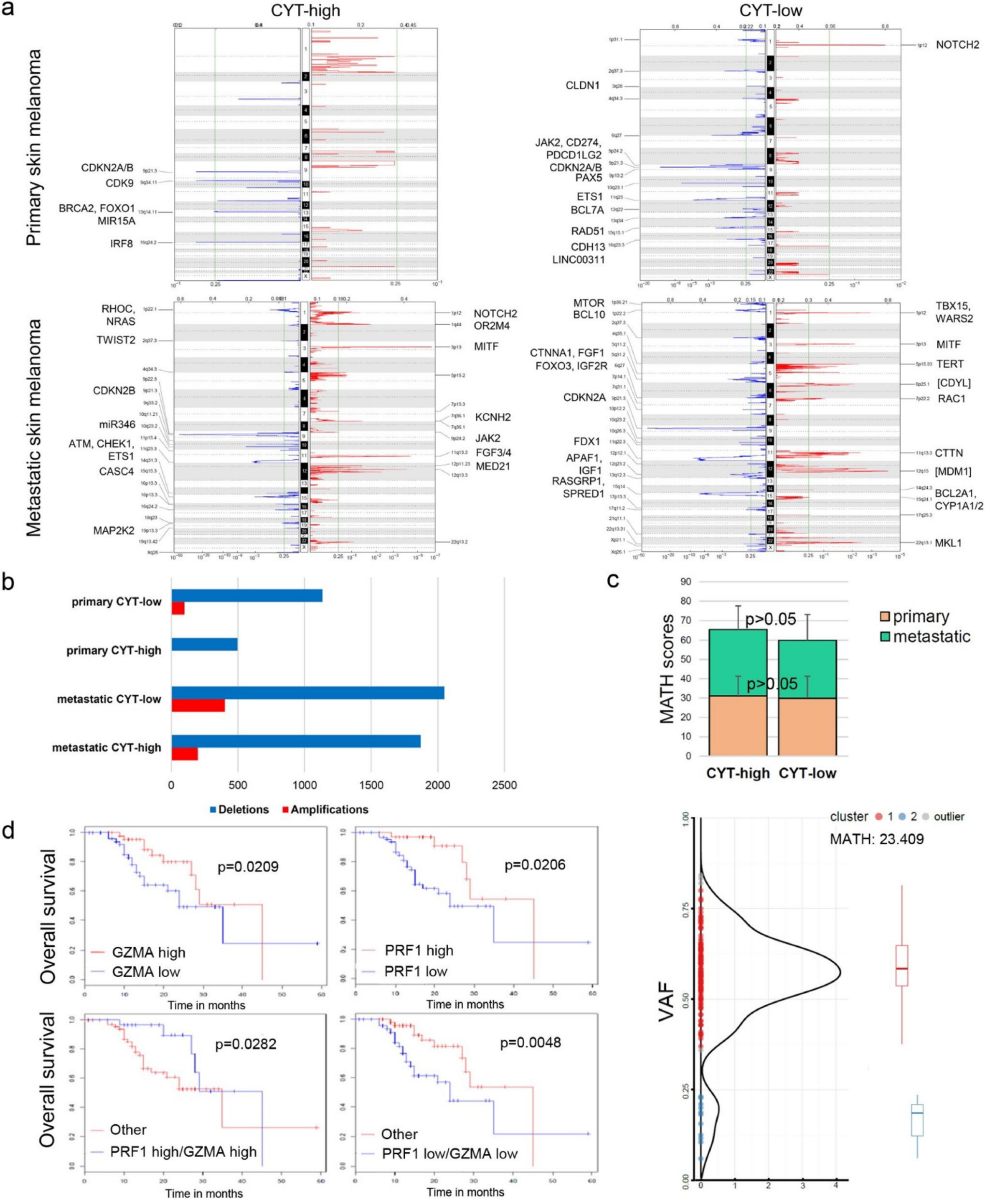
CYT correlates with different structural changes in melanoma. **a** Genomic locations of the amplified (red) or deleted (blue) chromosomal regions, within each cytolytic subset in primary and metastatic skin melanomas, as assessed by GISTIC2 analysis. In CYT-low primary skin melanomas recurrent amplifications were found at 1p.12 (NOTCH2), and deletions at 9p13.2 (PAX5), 11q25 (ETS1), 12q22 (BCL7A), 15q15.1 (RAD51) and 16q23.3 (CDH13). CYT-high metastatic melanomas (lower panel) had recurrent copy number amplifications in loci 1p12 (NOTCH2), 1q44 (OR2M4), 7q36.1 (KCNH2), 11q13.3 (FGF3/4), 12p11.23 (MED21) and 12q13.3 (R3HDM2) and deletions in 1p22.1 (RHOC, NRAS), 9p21.3 (CDKN2B), 10q23.2 (miR346), 11q23.3 (ATM, CHEK1, ETS1), 15q15.3 (CASC4). The x-axis represents the normalized amplification signals (top) and significance by Q value (bottom). The green line represents the significance cutoff at Q value = 0.25. **b** Overall, metastatic skin melanomas had significantly more recurrent SCNAs compared to primary tumors, but also CYT-low tumors had more SCNAs compared to CYT-high ones. **c** Tumor heterogeneity and CYT in SKCM. The two cytolytic subsets of skin melanomas had similar (*p* > 0.05) MATH scores. *P*-values were calculated using the Kruskal–Wallis test. Below is a representative plot depicting the distribution of the VAF and the two corresponding clusters in a skin melanoma sample (MATH = 23.409). **d** Synergistic analysis of GZMA and PRF1 showed that their over-expression (either alone or of both) led to a significant positive effect in SKCM patient survival; whereas, their simultaneous under-expression shifted significantly (*p* = 0.0048) toward a negative effect in patient survival

CDKN2A/B deletions (9p21.3) were common between CYT-high and CYT-low melanomas (both primary and metastatic). Also, amplifications in loci 1p12 (NOTCH2), 3p13 (MITF), 13q13.3 (FGF3/4, CTTN) and 22q13 (MLK1) were found in both cytolytic subtypes of metastatic melanoma (Fig. 4a). Overall, metastatic melanomas had significantly more recurrent SCNAs compared to primary tumors (602 amplifications and 3,920 deletions in metastatic SKCM relative to 99 amplifications and 1,632 deletions in primary SKCM), but also CYT-low tumors had significantly more SCNAs compared their CYT-high counterparts (Fig. 4b).

Taken together, the above findings show that specific types of somatic mutations and copy number aberrations are characteristic for each cytolytic subgroup in primary and metastatic melanoma.

To eliminate the possibility of assessing lower confidence of detecting somatic mutations and SCNAs due to tumor cellularity, we performed ABSOLUTE analysis and found no variance in the calculated cellularity estimates between CYT-high and –low SKCM samples. MATH scores ranged from 14.70 to 47.17 in primary and 14.59–62.22 in metastatic SKCMs. Neither the total mutation load, nor the total copy number events, correlated with tumor heterogeneity in melanoma. In addition, the MATH scores were similar between the two cytolytic subgroups (in primary SKCM, CYT-high vs. CYT-low, 31.04 ± 11.52 vs 29.86 ± 12.97, *p* > 0.05; in metastatic SKCM, 34.43 ± 12.02 vs 29.94 ± 13.31, *p* > 0.05), signifying that the dissimilarities in mutations and SCNA, are not due to a variable intra-tumor heterogeneity (Fig. 4c). Therefore, we propose that distinctive mutational and structural changes can discriminate skin melanomas of different cytolytic activity.

### Overexpression of GZMA and PRF1 synergistically affects patient survival

To determine whether CYT-high is a good prognostic indicator, we explored the overall survival of skin melanoma patients having high or low expression levels of *GZMA* and *PRF1*, using SynTarget.

High expression of each cytolytic gene, individually, was positively correlated with the overall melanoma patient survival (*p* = 0.02). The subgroup of non-metastatic melanoma patients having both genes over-expressed showed a significant positive effect in survival compared to the remaining patients (“other”) (*p* = 0.0282); whereas, simultaneous low levels of both genes shifted significantly (*p* = 0.0048) toward a negative effect (Fig. 4d). This observation implies that the overexpression of both GZMA and PRF1 genes, can synergistically affect the overall survival of skin melanoma patients.

### Chromothriptic events in cytolytic subsets of skin melanoma

Local chromosome shattering (chromothripsis) is a mechanism proposed to cause clustered chromosomal rearrangements, following chromosomal breaks at multiple locations, and the criteria for its inference were previously described [36]. Chromothripsis has been detected in 2–3% of cancers and involves impaired DNA repair [37]. Such genomic rearrangements may drive the development of cancer through the deletion of tumor suppressor genes or an increase in copy number of oncogenes, among other mechanisms [38]. The occurrence of chromothriptic events in skin melanoma, and their effect on genes associated with checkpoint inhibition and immune cytolytic activity is still unclear.

**Fig. 5:**
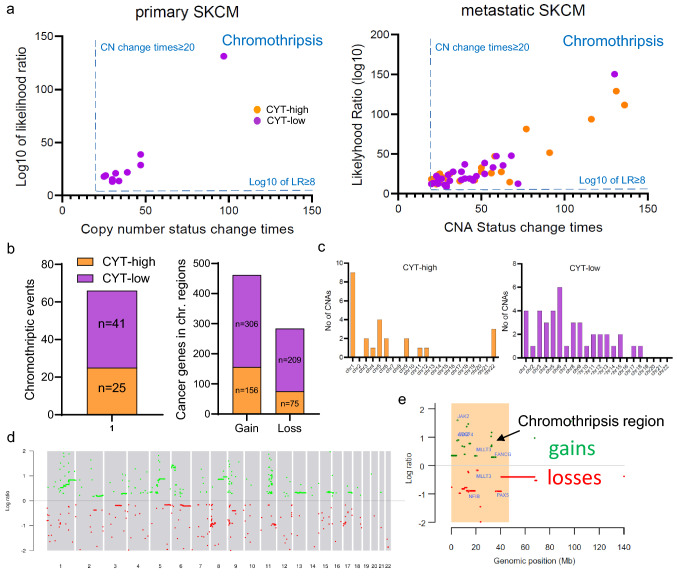
Chromothriptic events in cytolytic subsets of skin melanoma. **a** Skin melanomas exhibiting patterns of chromosomal chromothripsis (CN change times ≥ 20 and log10 of LR ≥ 8) are highlighted in the orange box. **b** The majority of chromothriptic events were harbored mainly in CYT-low (*n* = 41) and less in CYT-high (*n* = 25) skin melanomas. Importantly, the chromothriptic regions affecting chromosomal regions across CYT-low tumors were enriched in cancer genes, as these are registered in COSMIC. **c** The majority of chromothriptic events affected more chromosomes in CYT-low tumors. In specific, chromothriptic events largely affected chromosomes 1, 5 and 22 among CYT-high tumors, and 1, 3, 5 and 6among CYT-low tumors. **d** The general view of copy number aberration profile in a representative SKCM tumor (TCGA_SKCM_955602). Green dots depict genomic gains; whereas, red dots, genomic losses in this tumor sample. **e** Indicative chromothripsis region in chromosome 9 of a skin melanoma (TCGA_SKCM_955602) with chromosomal gains and losses. COSMIC cancer genes are denoted

Overall, we detected chromothriptic events of various sizes in 211/470 (44.89%) skin melanomas (Fig. 5a, d, e). Among these, 25 belonged to the CYT-high and 41 to the CYT-low subgroups (average CNA status change times; CYT-high vs CYT-low SKCM, 50.6 ± 35.17 vs 41.12 ± 21.59). Notably, we observed distinct patterns of chromothriptic events between the two cytolytic subgroups, the majority of which, were harbored mainly in CYT-low skin melanomas (41/66, 62% of the chromothriptic events, CN change times ≥ 20 and log_10_ of LR ≥ 8) (Fig. 5b) and affected a higher number of chromosomes in CYT-low tumors (Fig. 5c). Interestingly, the chromosomal regions among CYT-low skin melanomas that harbored chromothripsis, contained a higher number of cancer genes, including *KRAS*, *NOTCH2*, *BCL9*, *CCND1* (gains) and *BRAF*, *NRAS*, *PAX3*, *ATM* and *CD274* (losses) (in total, 306 gains and 209 losses among CYT-low tumors vs. a total of 156 gains and 75 losses among CYT-high tumors) (Fig. 5b and Table S4).

Overall, our findings support the existence of a broader pattern of chromothripsis across cytolytic low skin melanomas.

### Increased neoantigen load associates with high cytolytic levels in primary skin melanomas

Cancer neoantigens arise from mutated peptides and favorably drive T-cell recognition of cancer cells [39]. They are therefore, an attractive immune target since their selective expression in cancer cells can minimize immune tolerance.

**Fig. 6:**
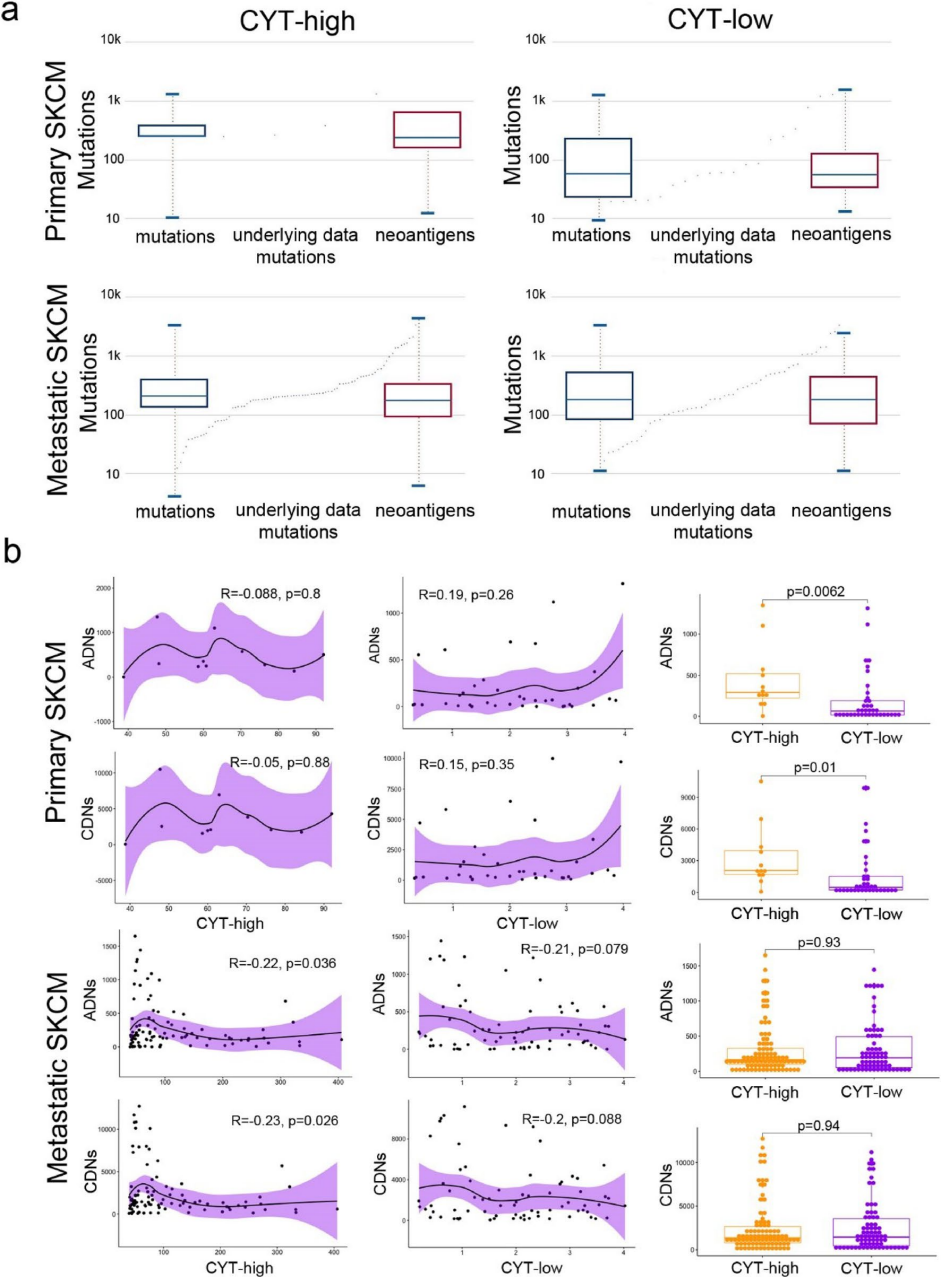
Increased neoantigen load associates with high cytolytic levels in primary skin melanomas. **a** Both the number of total mutations and neoantigens, was significantly higher within CYT-high primary skin melanomas (SKCMs), compared to CYT-low ones. On the other hand, the total mutation load, as well as the neoantigenic load did not differ between the two cytolytic subgroups of metastatic skin melanomas. **b** CYT-high primary (and not metastatic) tumors contain significantly more classically (CDNs) and alternatively defined (ADNs) neoantigens; but these, were not correlated with a high cytolytic activity

It was of interest to confirm whether cancer neoantigents associate with high cytolytic levels in skin melanoma. Analyzing mutation data from the Cancer Immunome Database (TCIA), it was evident that primary CYT-high skin tumors contained a higher number of neoantigens, apart from mutations, compared to their CYT-low counterparts. This was not evident, however, in metastatic CYT-high tumors (Fig. 6a).

To investigate this further, we predicted the missense mutations that could potentially function as T-cell neoepitopes in skin melanomas, using *antigen.garnish*. Our results confirmed that CYT-high primary (and not metastatic) tumors are significantly enriched in (classically and alternatively defined) neoantigens, but these, were not correlated with a high cytolytic activity (Fig. 6b). In addition, the tumors’ MATH scores did not correlate with their neoantigen load. In total, these data support that a high cytolytic activity is driven by a high mutational burden, as well as neoantigenic load, only in primary skin melanomas.

### Prediction of skin melanoma patients’ response to immune checkpoint inhibition

Since just a subgroup of skin melanoma patients responds to immune checkpoint inhibition, the need to elucidate the mechanisms of resistance and predict markers is high. TILs, the expression of PD-1 or PD-L1 [15, 40], the mutational load [41], or clonal neoantigens [39], have all been proposed as markers; however, none of them has been fully validated, yet.

Hypothesizing that CYT-high skin melanomas have higher immunophenoscore due to increased immunogenicity, which in turn results in better prognosis and response to therapy, we analyzed two data sets containing skin melanoma patients, who, if treated with anti-CTLA-4 and/or anti-PD-1 inhibitory molecules, we could use their IPS to predict their clinical response.

**Fig. 7:**
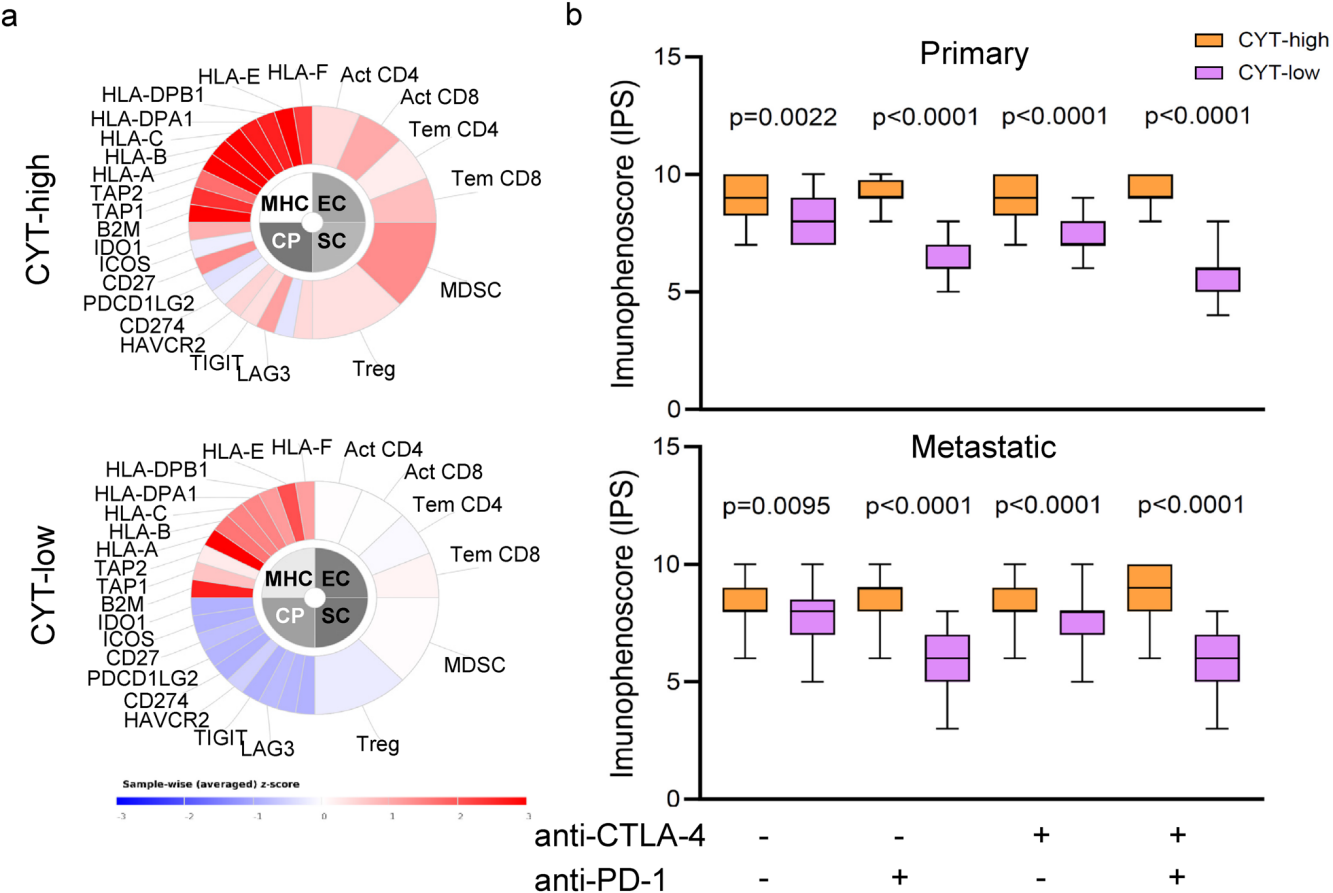
Prediction of skin melanoma patients’ response to immune checkpoint inhibition. Indicative immunophenograms depicting the immunophenoscores (IPS) across distinct cytolytic subgroups in primary and metastatic skin melanomas. **a** Tumors of the expected responders were enriched in cytotoxic cells (CD8 + T cells, γδT cells, NK cells) and depleted of MDSC and Treg cells. Sample-wise z-score from gene expression of all cell types included in any of the ten best predictors within each cancer type are color coded and divided into four categories; MHC molecules (MHC), Immunomodulators (CP), Effector cells (EC), and Suppressor cells (SC). The outer part of the wheel includes individual factors; whereas, the inner wheel illustrates the weighted average z-scores of the factors included in the particular category. **b** Average IPS scores across the two cytolytic subgroups in primary and metastatic SKCMs. Overall, CYT-high tumors had significantly higher IPS, which is indicative of a better response to CTLA-4 and PD-1 blockade. The difference in IPS was more dramatic in patients who were to receive combination therapy either with both CTLA-4 and PD-1 blockers [anti-CTLA4( +), anti-PD-1( +)], or with one of the two [anti-CTLA4( +), anti-PD-1(−) and anti-CTLA4(−), anti-PD-1( +)] compared to patients under no expected immune checkpoint inhibition therapy [CTLA4(−), anti-PD-1(−)], (*p* < 0.0001)

Assessed globally, CYT-high skin melanomas (both primary and metastatic) had significantly higher HLA levels compared to the intratumoral mean expression. In contrast, CYT-low melanomas were characterized by downregulation of several immune checkpoints, compared to the intratumoral mean expression. Importantly, we could observe similar patterns across patients who were to be treated with anti-CTLA-4 alone or combined with anti-PD-1, as well as across patients who were to be treated with anti-PD-1, alone (Fig. 7a, b). In addition, CYT-high SKCMs contained considerably higher numbers of cytotoxic cells (CD8 + T cells, γδT cells, NK cells), and lower numbers of the suppressor MDSC and Treg cells.

As expected, CYT-high SKCM patients had a markedly higher IPS (and consequently, an expected clinical benefit) compared to CYT-low patients, who were not expected to receive checkpoint inhibition. Interestingly, the IPS scores were significantly higher across all CYT-high tumors (both primary and metastatic), upon treatment with CTLA-4 or PD-1 blockers alone, or a combination of both (*p* < 0.0001) (Fig. 7c).

Our data suggest that the IPS has a predictive value in CYT-high melanoma patients who were to receive CTLA-4 and PD-1 inhibition therapy, and are in accordance with previous observations that patients with higher levels of tumor cytolytic activity, and expression of immune checkpoints, benefited more from the corresponding immune checkpoint blockers [42].

## Discussion

A deeper understanding of the complex immunobiology of skin melanoma, including the mechanisms of immunosurveillance and immunoediting, which contribute to the tumor’s resistance to immune checkpoint inhibition therapies, will confidently help scientists develop more effective immunotherapeutic approaches in the near future.

Here, we extensively analyzed the gene expression and genomic landscape of skin melanoma in the context of intratumoral immune cytolytic activity [43]. We stratified primary and metastatic tumors according to their immune cytolytic levels, and discriminated a subgroup of them with noticeable T-cell reactivity. We show that CYT-high skin melanomas are significantly enriched for immune-related gene sets linked with activated CD8 + T cells, B cells, M1 macrophages, activated dendritic cells and NK cells, among others, corroborating the existence of an inflamed tumor microenvironment in these patients. In contrast, the CYT-low tumors were enriched for non-immune-related gene sets, primarily related with monocytes and CD4 + T cells.

Abundant CD8 + T cell infiltrates are well known to exist in inflamed metastatic melanoma and drive the upregulation of PD-L1, IDO and Tregs in the tumor microenvironment [44]. Additionally, CD8 + T-cell infiltration associates with a better response of cancer patients to chemotherapy [45], neoadjuvant therapy [46] and anti–PD-1 immunotherapy [47]. A key role for tumor-infiltrating B cells in modulating the anti-tumor immune response, was recently proposed in skin melanoma [48]. B cells play an important prognostic role and can predict non-response to an immune checkpoint inhibitor in metastatic melanoma [48]. The enrichment of CYT-high tumors in M1 macrophages is in line with their suppressive role in cancer progression, while that of M2 macrophages in CYT-low tumors, underscores their role in favoring tumor growth and dissemination [49]. Activated dendritic cells are also important in the immune response against cancer cells and can be used as a strong independent prognostic factor [50]; whereas, activated NK cells can also efficiently kill malignant melanoma cells [51].

Taken together, these findings propose the stratification of skin melanoma patients according to their gene expression profiling, which can distinguish them between those having a strong cytolytic T-cell response, and those with a less privileged immune microenvironment. A similar, CYT-based stratification was previously performed for other types of cancer [7, 14, 15].

We also showed that the mutational burden is higher among metastatic tumors, and that the increased mutational and neoantigen load that elicits an immune response by TILs, associates with high cytolytic levels in primary skin melanomas. In a similar study, a high mutational burden was previously linked with response in melanoma patients who were treated with immune checkpoint inhibitors, but not with the patients’ overall survival [52]. CYT and the mutational load, along with the expression of the PD-axis genes, were also proposed as interdependent factors that associate with survival in melanoma [40].

Both cytolytic subgroups of metastatic melanomas included driver mutations in some well-defined genes (*BRAF, NRAS, CDKN2A, TP53* and *PTEN*) [34]. In addition, our data provide evidence that distinct cytolytic subgroups in primary and metastatic skin melanoma have different patterns of significantly mutated genes. We found that CYT-low primary tumors associate with mutations in *BRAF, DMRT3, GSTA5, TP53, CRYBA4, STRA13, PRAMEF12, RNF32, SLC1A6, TEKT2* and *NRAS*; whereas, CYT-high primary tumors with *LCK, GSTSF1L, BMF, CSTL1, DRGX, ENTPD3, CAMK4, GPR151* and *LTF*.

Similarly, we show that CYT-low metastatic tumors associate with mutations in *GALNTL5, RGPD4, DNAJC5B, DEFA3, IRF2, COL2A1, VEGFC, WFDC11, PROL1, CD80, ADAM18, APOBEC3H* and *CSHL1*; whereas, CYT-high metastatic tumors with *PPP6C, DSG1, DEFB112, SPATA16, STARD6, RARRES2 PDE1A, IQCF3, KLK8, CDKN2A, SPANXN5, C16orf90, CD48, TXNDC3* and *RAC1*.

Although many of these mutations were previously characterized in skin melanoma (e.g., *BRAF*, *NRAS*, *TP53*, *PPP6C, CDKN2A, RAC1*, etc.) [3, 34], to our knowledge, this is the first report that appreciates them in association with tumors of different cytolytic activity.

We also provide evidence that different genomic structural variations are implicated in the progression of distinct cytolytic subgroups in skin melanoma. We show that CYT-low skin melanomas have a higher number of amplifications and deletions in their genome. These, include mainly recurrent *NOTCH2* amplifications, and non-silent mutations and/or deletions in *PD-L1, PD-L2, CDKN2A/B, PAX5, ETS1, BCL7, RAD51, JAK2, APAF1, FOXO3, CTNNA1* and *IGF1*, among others. These genomic structural variations have a profound effect on immune activation in these tumors.

Other distinct chromosomal aberrations included *JAK2, KCNH2, MED21* and *R3HDM2* amplifications or *NRAS*, *RHOC, CDKN2B, ATM, CHEK1* and *ETS1* deletions, and were associated with CYT-high tumors.

Many of them were previously appreciated in the genome of skin melanomas (e.g., *MITF, NOTCH2, PD-L1* and *JAK2* amplifications, or *CDKN2A* deletions [3]); however, the CNAs in genomic locations harboring *PAX5, ETS1, BCL7, RAD51, APAF1, FOXO3* and *CTNNA1* are novel.

Following these observations, we detected marked differences in the chromothriptic pattern between CYT-high and CYT-low tumors. These regarded the prevalence of chromothriptic events, the number of chromothriptic chromosomes per tumor and the cancer genes that they harbor. Such differences suggest that distinct mechanisms could give rise to chromothripsis across the two cytolytic subgroups in skin melanoma.

In addition, we highlight several targetable immune-related genes, other than *PD-1, PD-L1/2* and *CTLA-4*, which may coordinately contribute to immunosuppression. *IDO1* and *LAG3* overexpression, which was evident in CYT-high skin melanomas, provides an alternative immunosuppressive barrier that is needed to hamper the antitumor activity of CTL and NK cells, in these tumors. This is something that was initially proposed in skin melanoma cell lines [53], and later corroborated at the human level, by us and others [7, 15]. We suggest that combined immune inhibition of these markers along with the PD-1/PD-L1 axis and/or CTLA-4, could provide a better therapeutic outcome for CYT-high patients.

Together with the synergistic effect of GZMA and PRF1 in patient survival, we provide evidence that CYT-high skin melanoma patients who are to receive CTLA-4 and/or PD-1 checkpoint blockade therapy, have a markedly higher immunophenoscore and are consequently, expected to have a clinical benefit, compared to CYT-low patients.

## Conclusions

In the aggregate, we highlight new links between certain expressional or genomic changes and the activation of an antitumor immune response in skin melanoma. Our findings also corroborate that immunotherapies with PD-1 and/or CTLA-4 blockade, should be able to overcome resistance and broaden the clinical benefit, especially for CYT-high skin melanoma patients.

## Supplementary Information

Below is the link to the electronic supplementary material.

Supplementary file1 (XLSX 9 kb)

Supplementary file2 (PDF 426 kb)

Supplementary file3 (PDF 405 kb)

Supplementary file4 (PDF 125 kb)

Supplementary file5 (PDF 795 kb)

Supplementary file6 (PDF 801 kb)

Supplementary file7 (PDF 73 kb)

Supplementary file8 (PDF 150 kb)

Supplementary file9 (PDF 563 kb)

Supplementary file10 (PDF 656 kb)

Supplementary file11 (PDF 64 kb)

Supplementary file12 (PDF 55 kb)

## Data Availability

All data generated or analyzed during this study are included in this published article [and its supplementary information files].

## Abbreviations

AND: Alternatively defined neoepitope
CDN: Classically defined neoepitope
CNA: Copy number alteration
CTL: Cytotoxic T cells
CTLA-4: Cytotoxic T-lymphocyte-associated protein 4
CYT: Immune cytolytic activity
GZMA: Granzyme A
MATH: Mutant-allele tumor heterogeneity
MSigDB: Molecular Signatures Database
NK cells: Natural killer cells
PD-1: Programmed cell death protein 1
PD-L1: Programmed death-ligand 1
PRF1: Perforin-1
SKCM: Skin melanoma
SMGs: Significantly mutated genes
TCGA: The Cancer Genome Atlas
VAF: Variant allele frequency
