# Supplementary figures and images for: Distinct genomic features across cytolytic subgroups in skin melanoma
Source: Cancer Immunol Immunother. 2021 Mar 29;70(11):3137–54. doi: 10.1007/s00262-021-02918-3 (PMC8505325; doi:10.1007/s00262-021-02918-3)

a

CYT-high

CYT-low

Primary SKCM

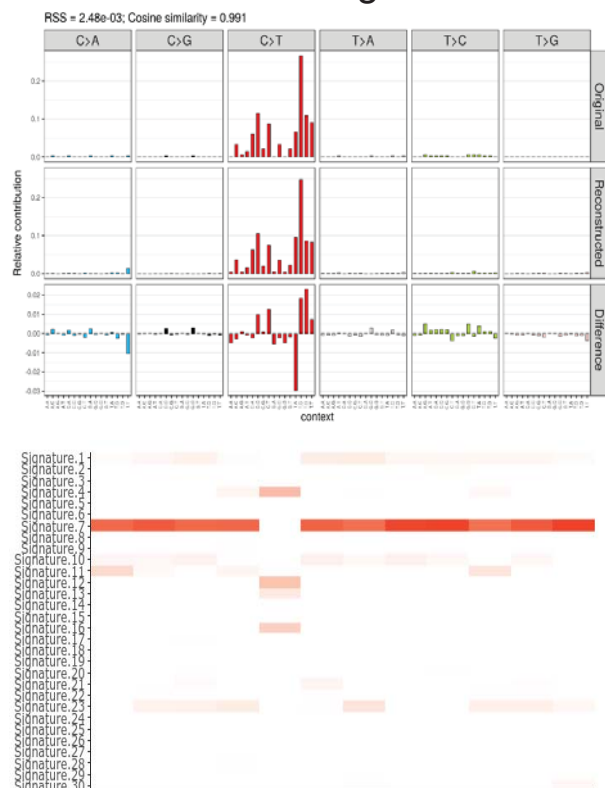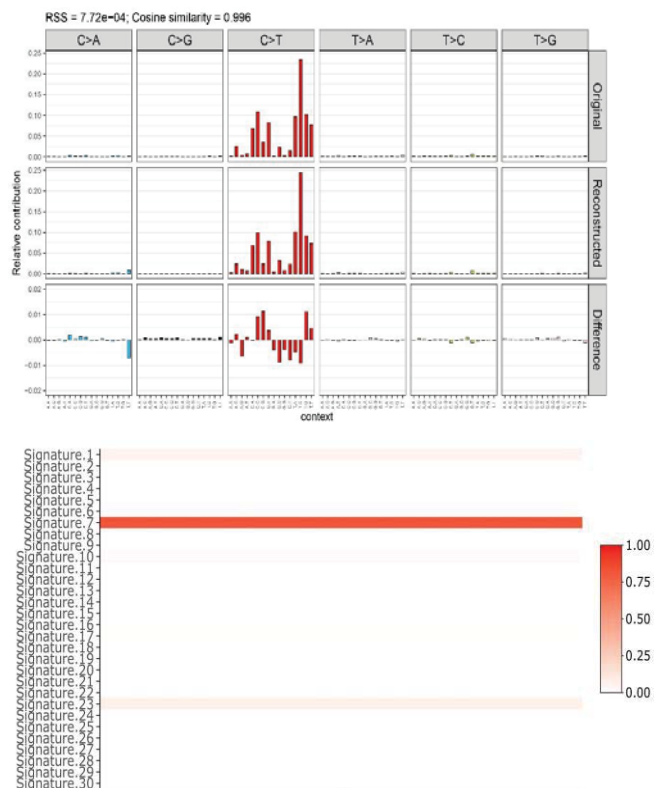

b

Metastatic SKCM

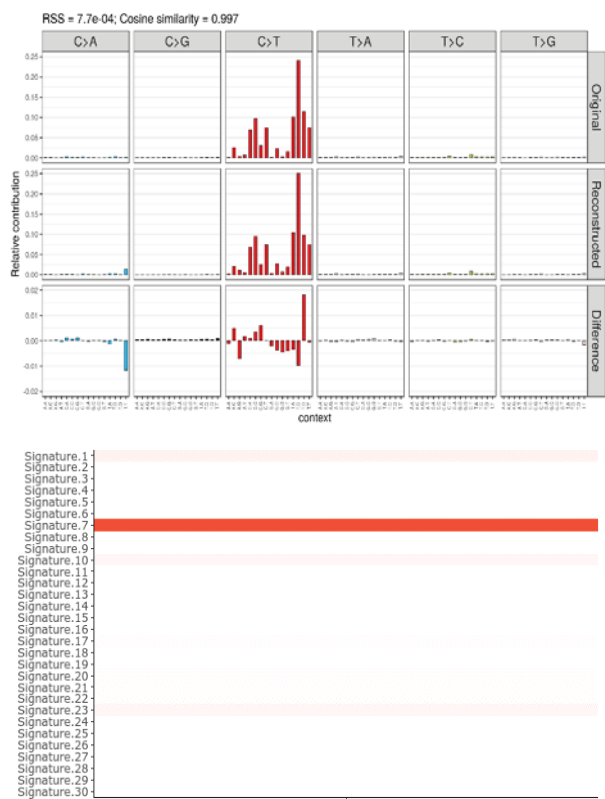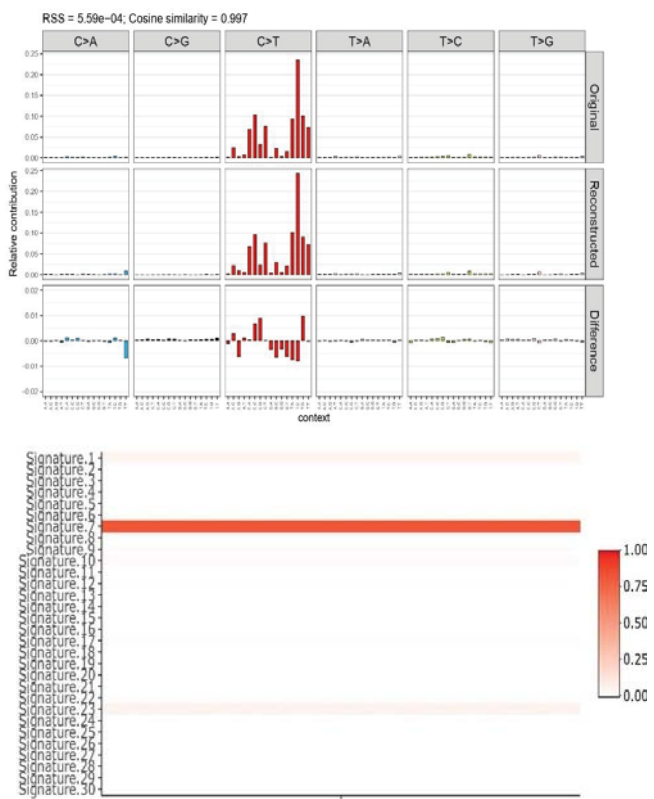

c

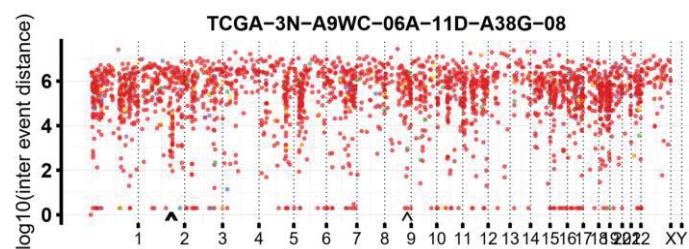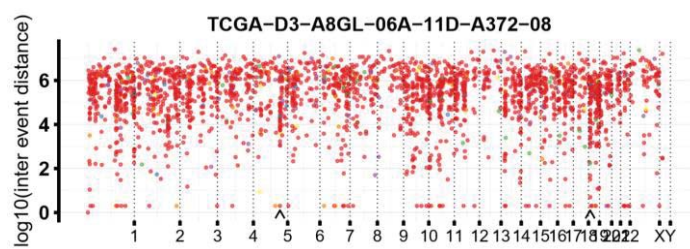

● C>A ● C>T ● T>C  
● C>G ● T>A ● T>G

Supplement: Supplementary file 2 — Supplementary file2 (PDF 426 kb) [file 262_2021_2918_MOESM2_ESM.pdf]

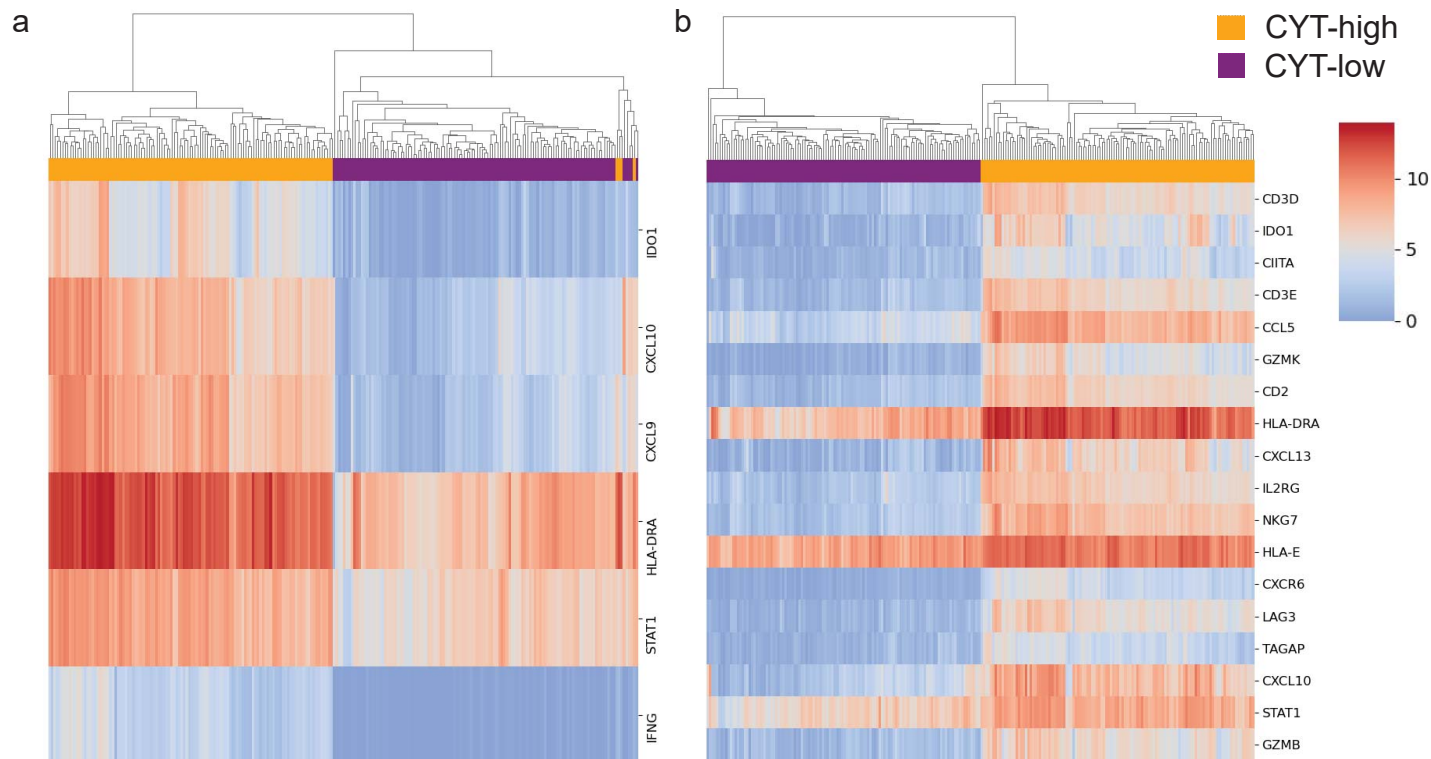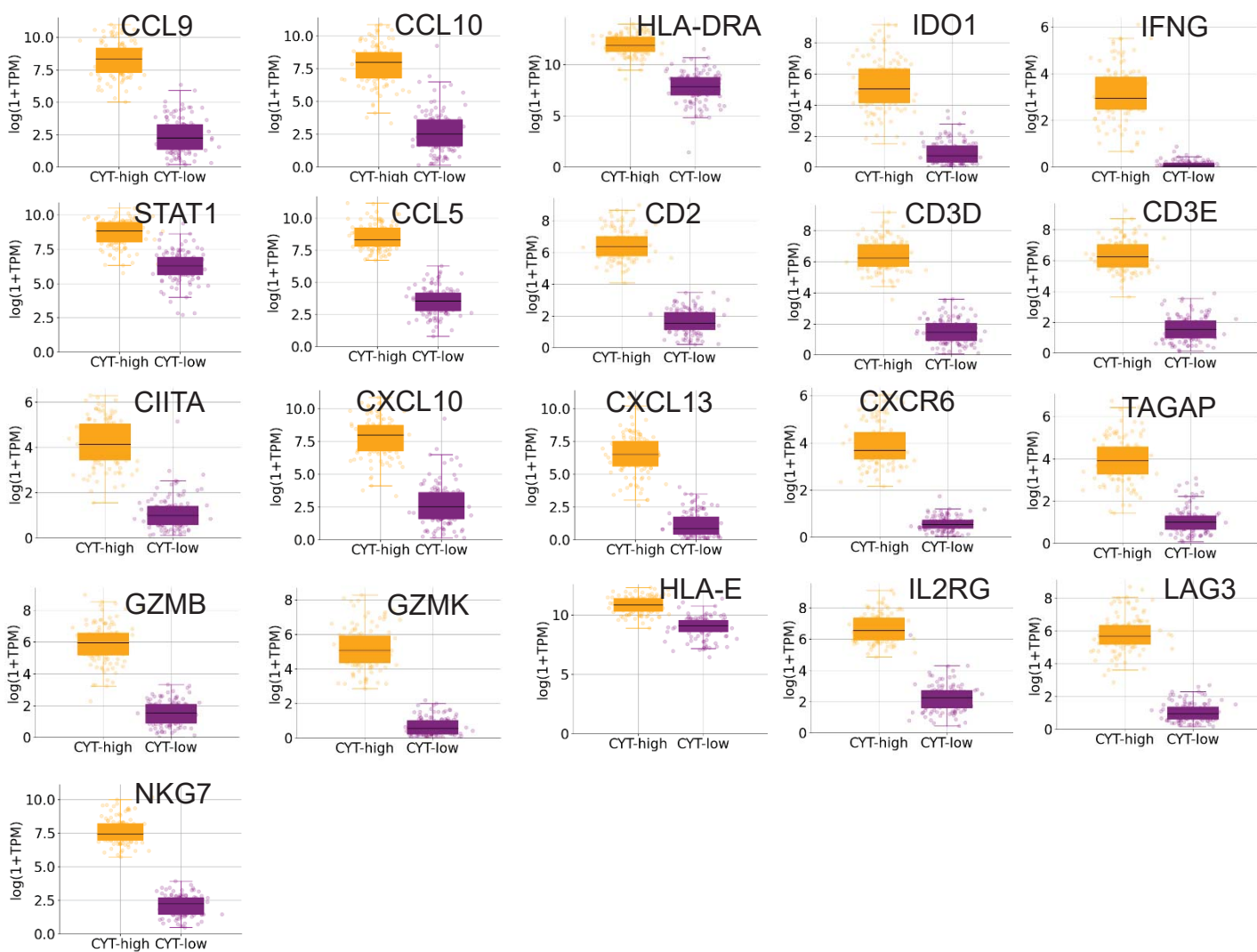

Supplement: Supplementary file 3 — Supplementary file3 (PDF 405 kb) [file 262_2021_2918_MOESM3_ESM.pdf]

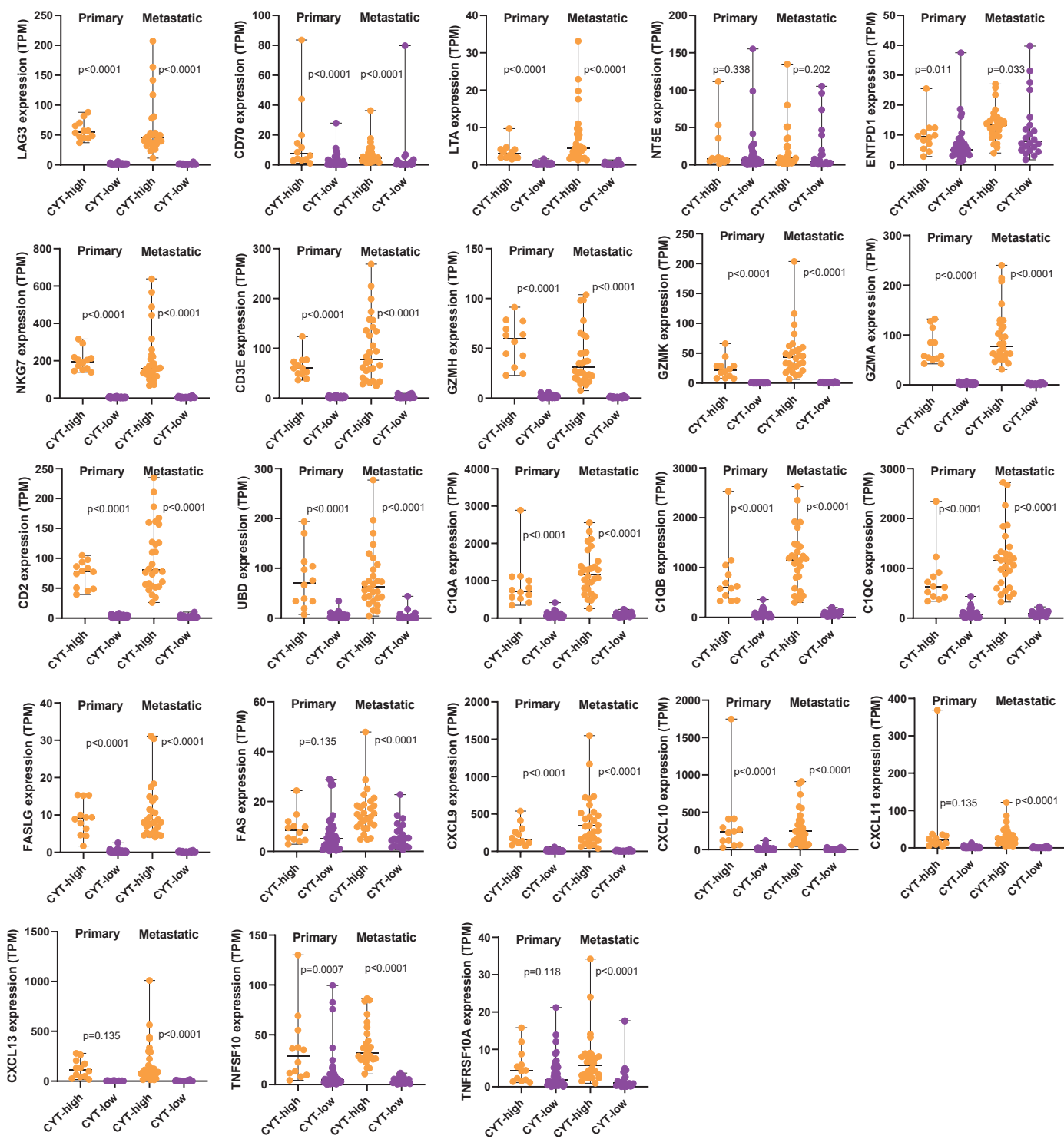

Supplement: Supplementary file 4 — Supplementary file4 (PDF 125 kb) [file 262_2021_2918_MOESM4_ESM.pdf]

a

## Primary SKCM

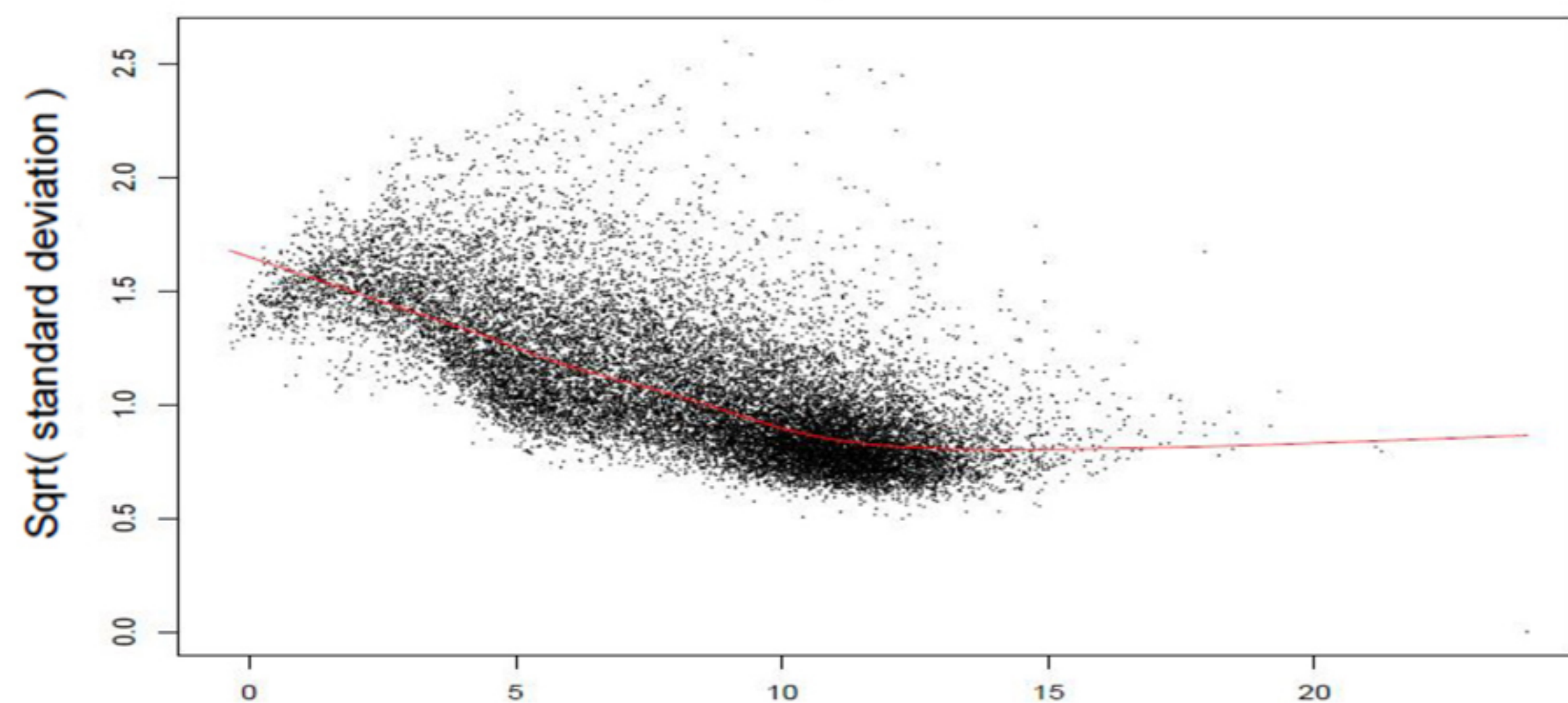

## Metastatic SKCM

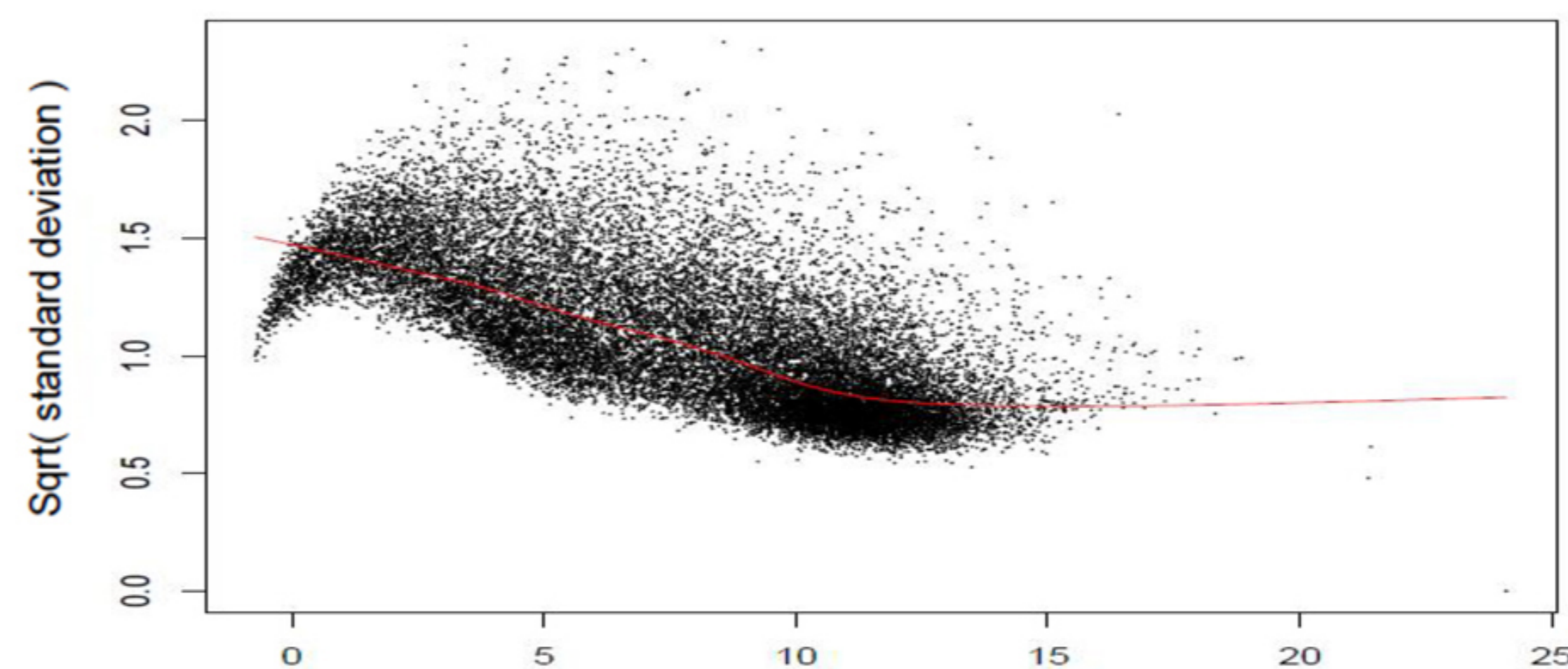

b

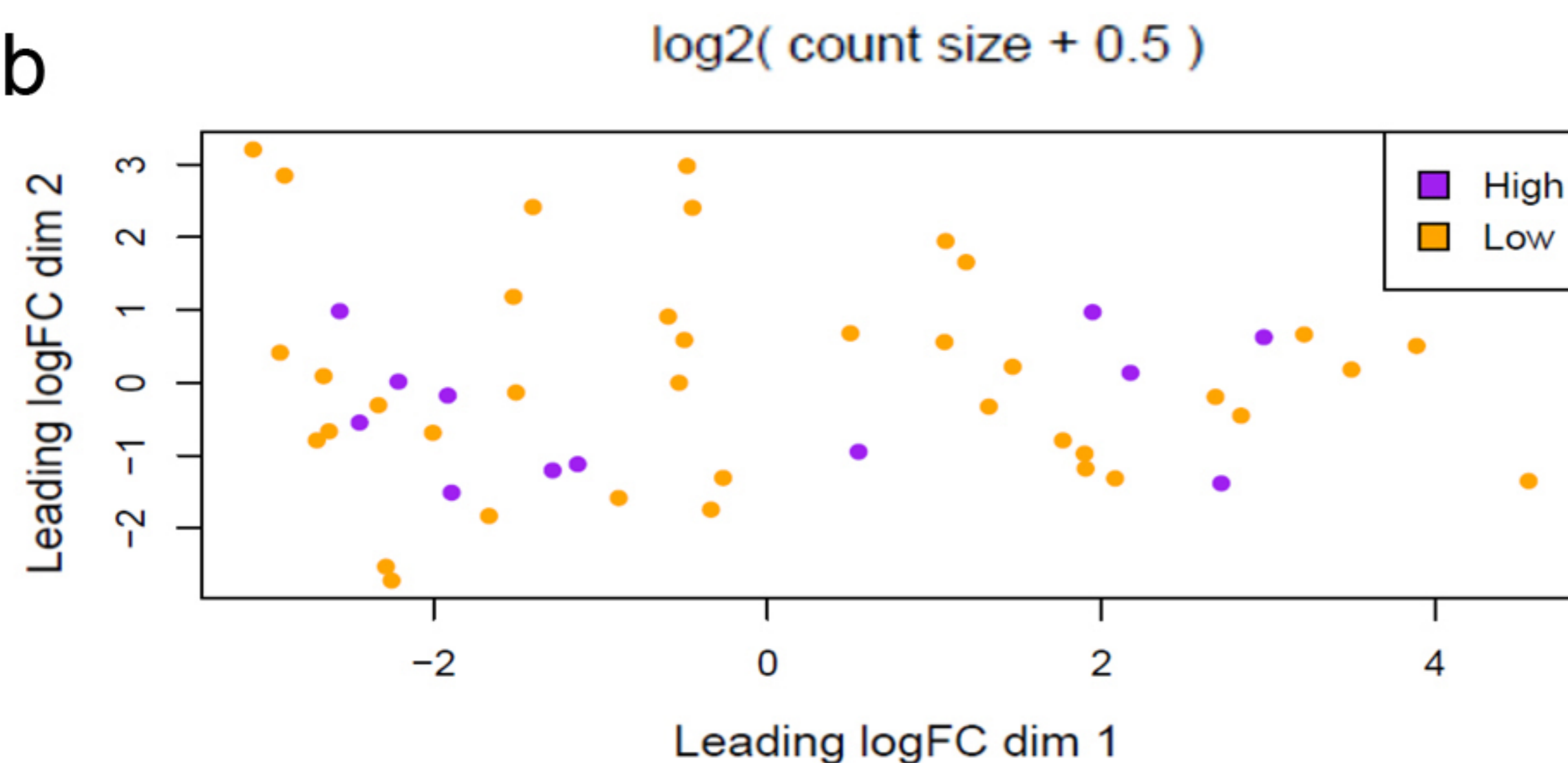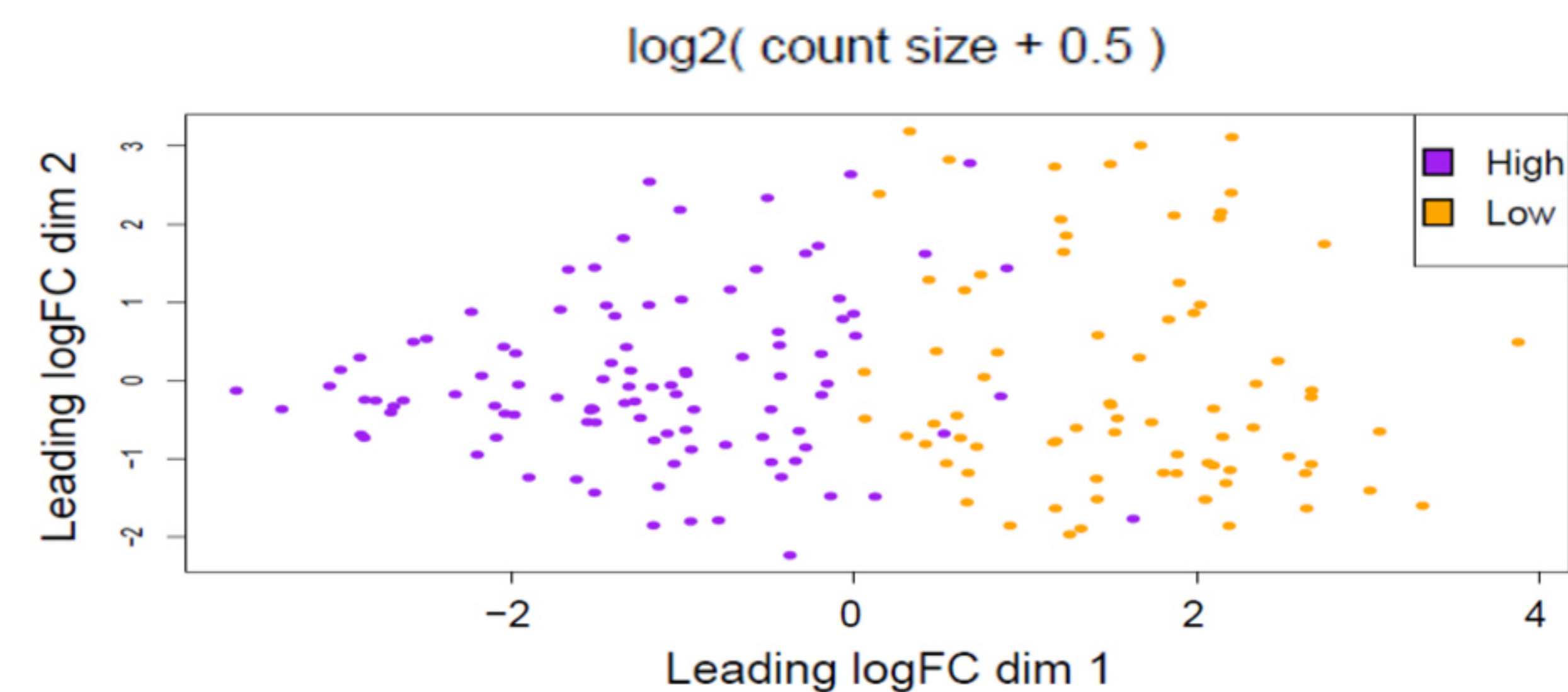

c

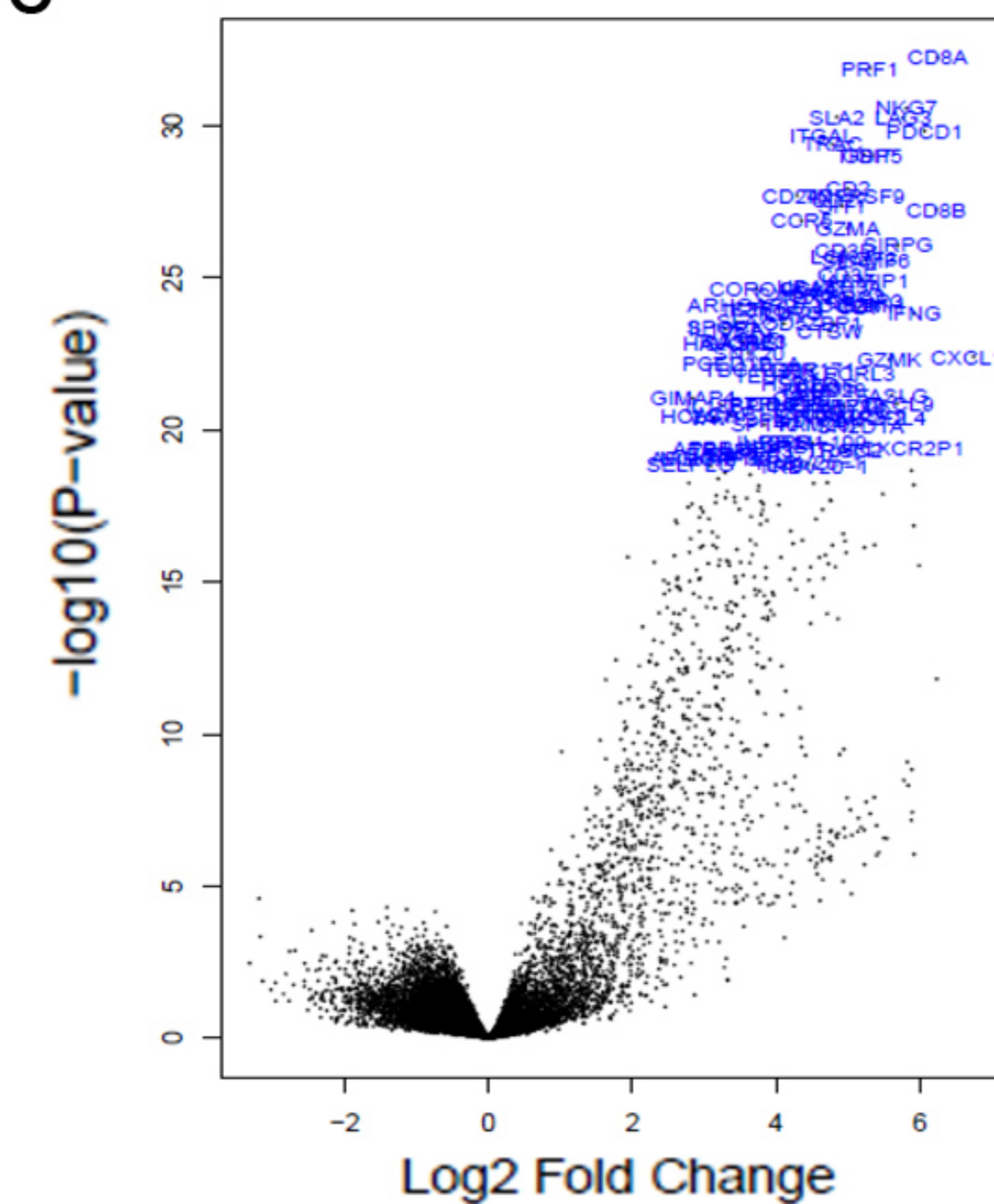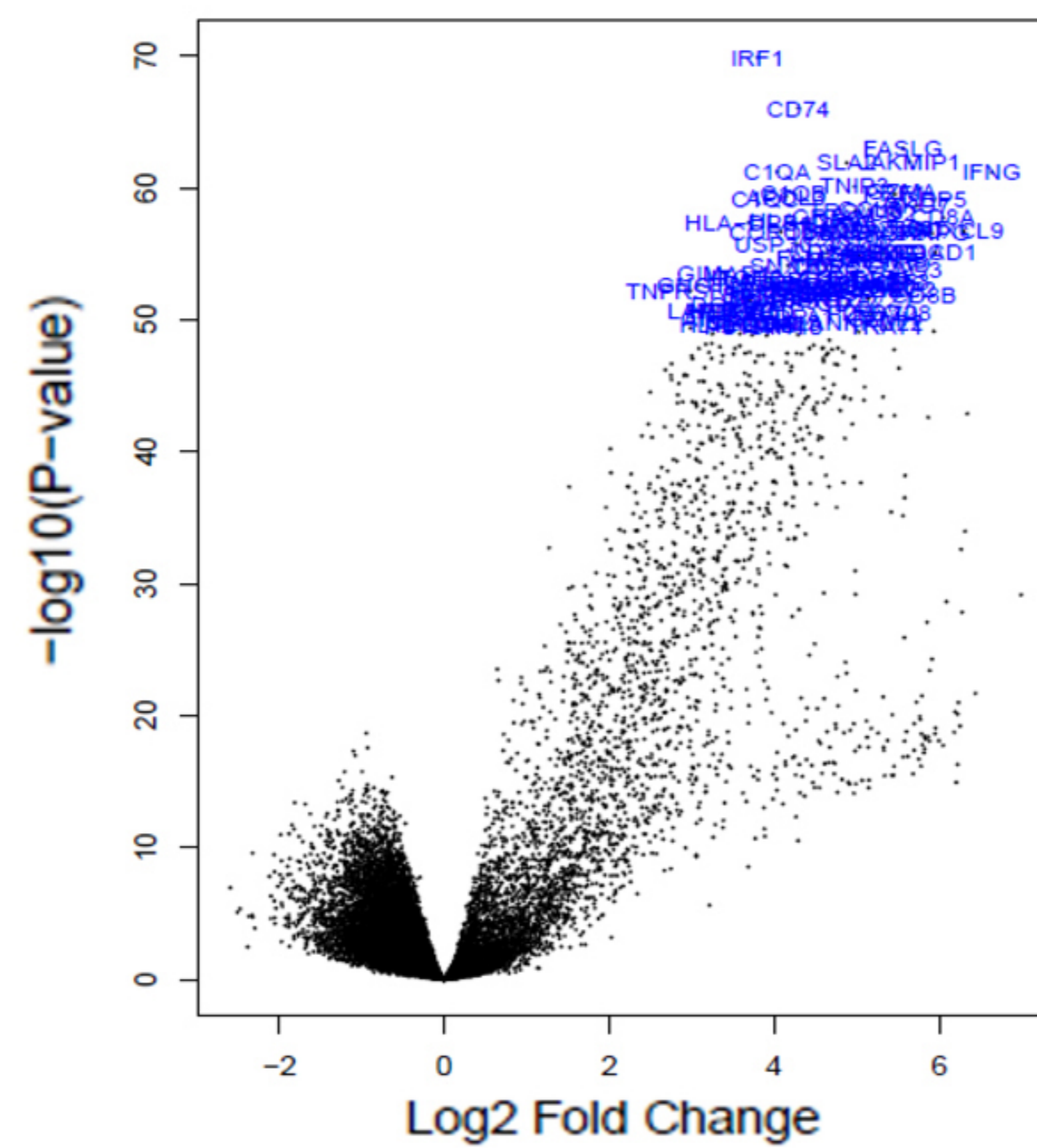

d

Primary SKCM

Metastatic SKCM

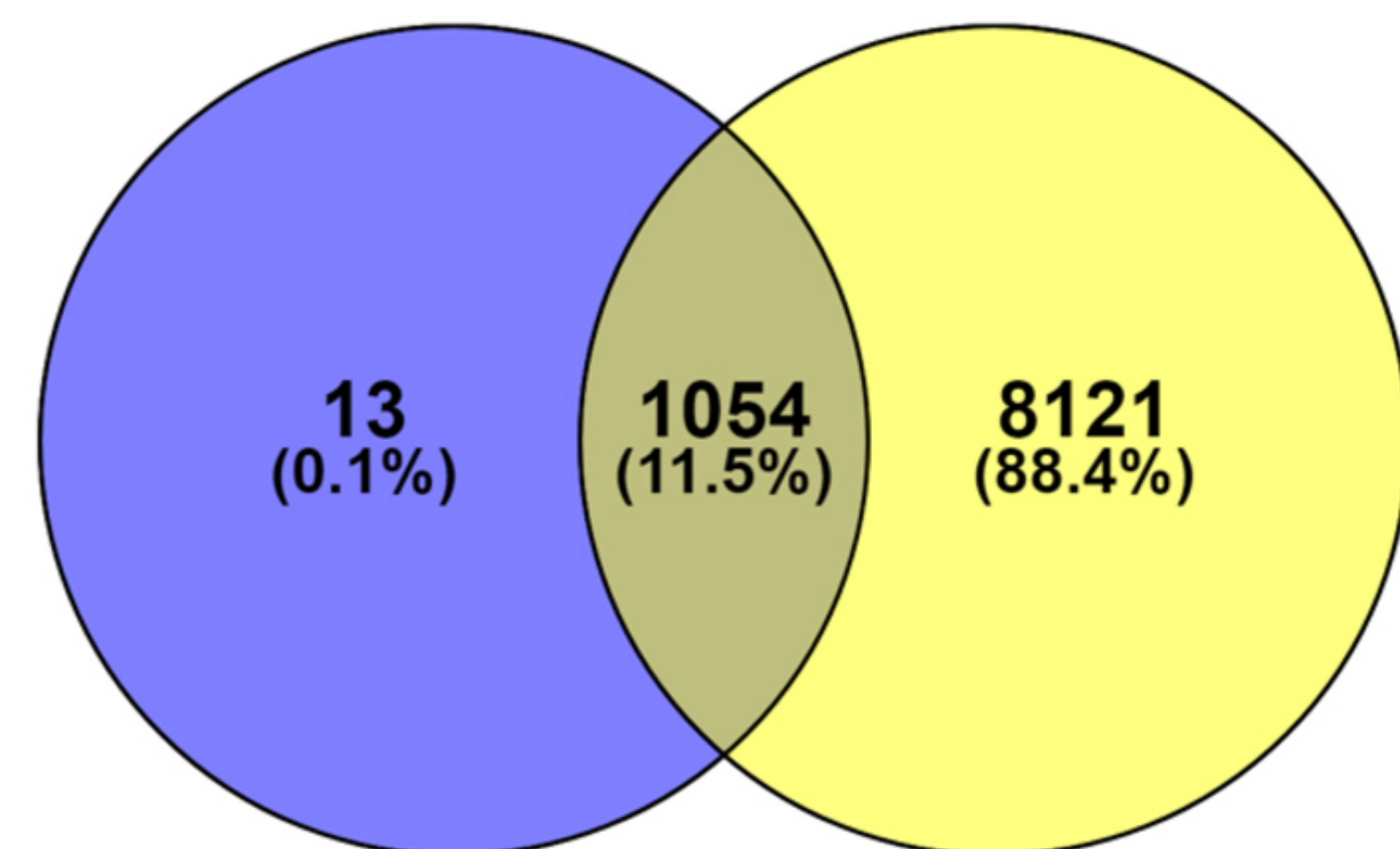

Supplement: Supplementary file 6 — Supplementary file6 (PDF 801 kb) [file 262_2021_2918_MOESM6_ESM.pdf]

a

## Primary SKCM

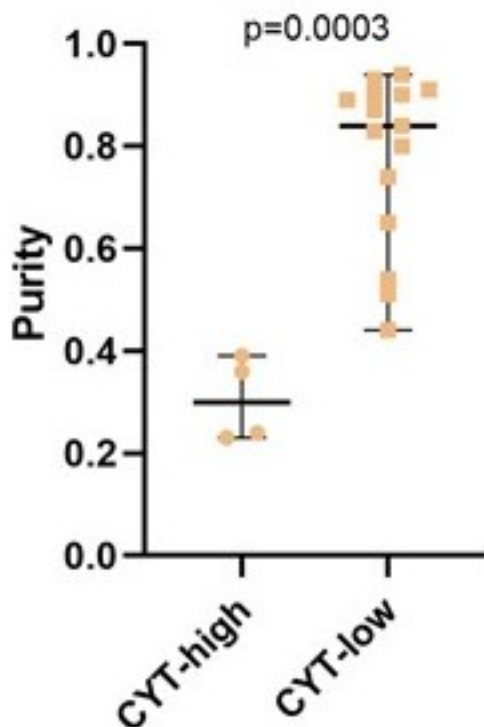

## Metastatic SKCM

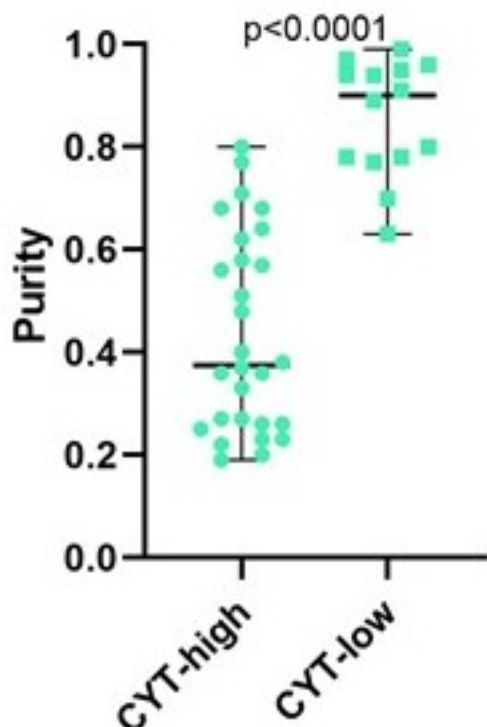

b

## Primary SKCM

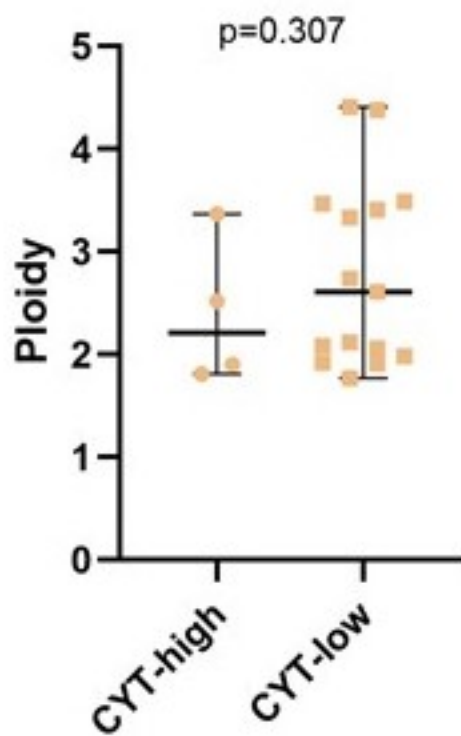

## Metastatic SKCM

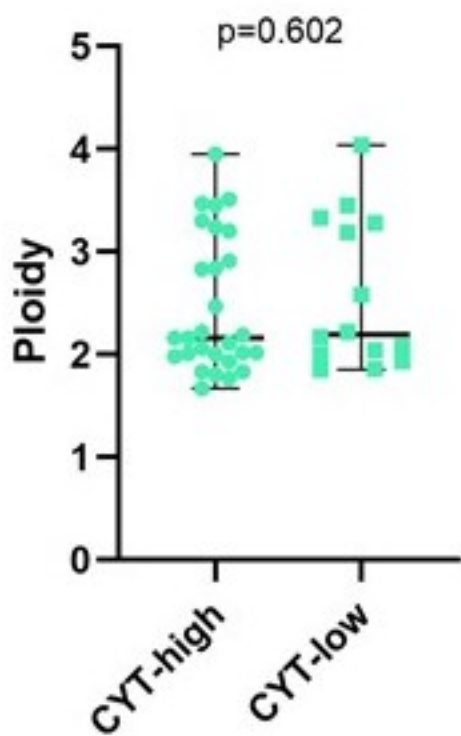

Supplement: Supplementary file 7 — Supplementary file7 (PDF 73 kb) [file 262_2021_2918_MOESM7_ESM.pdf]

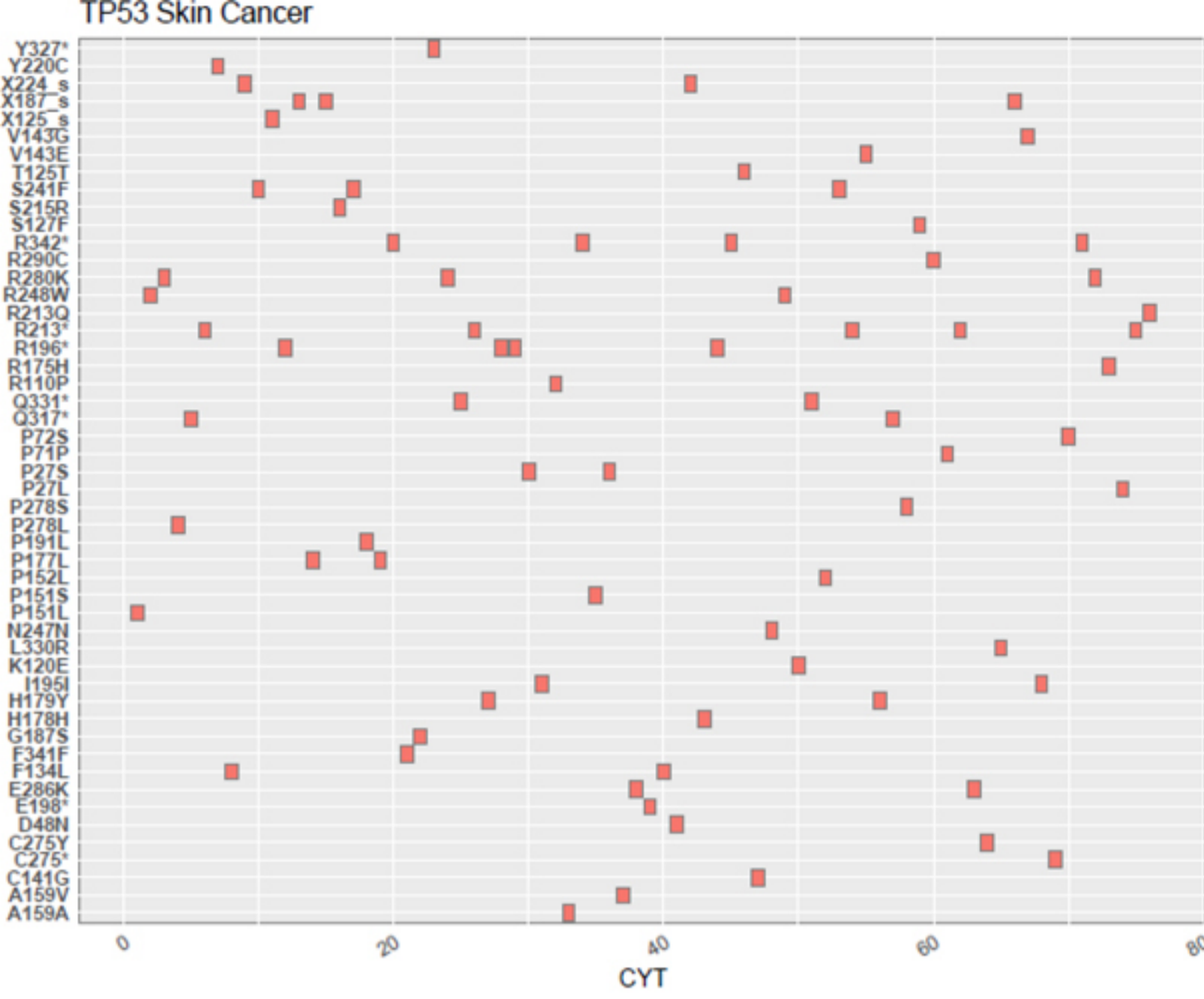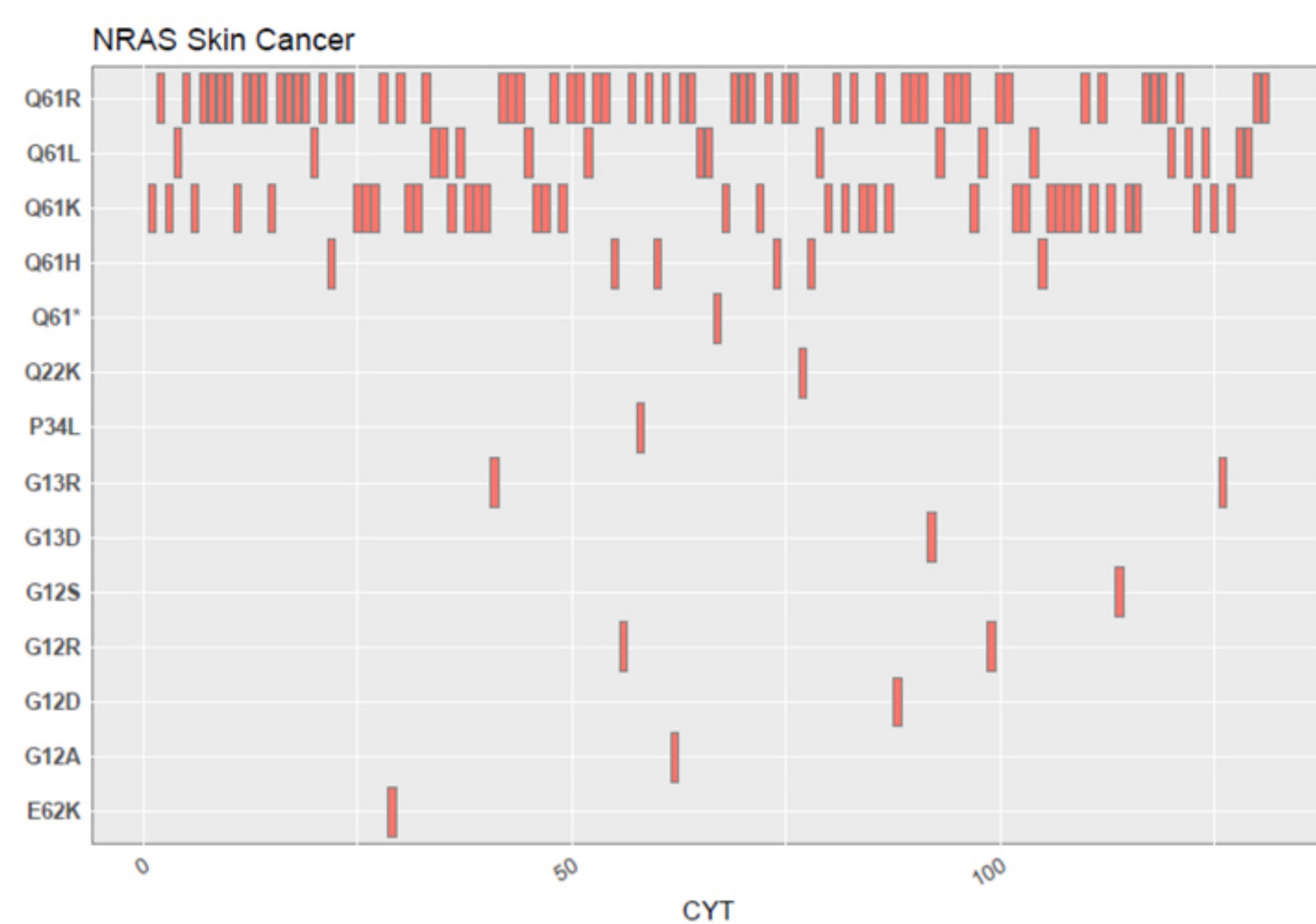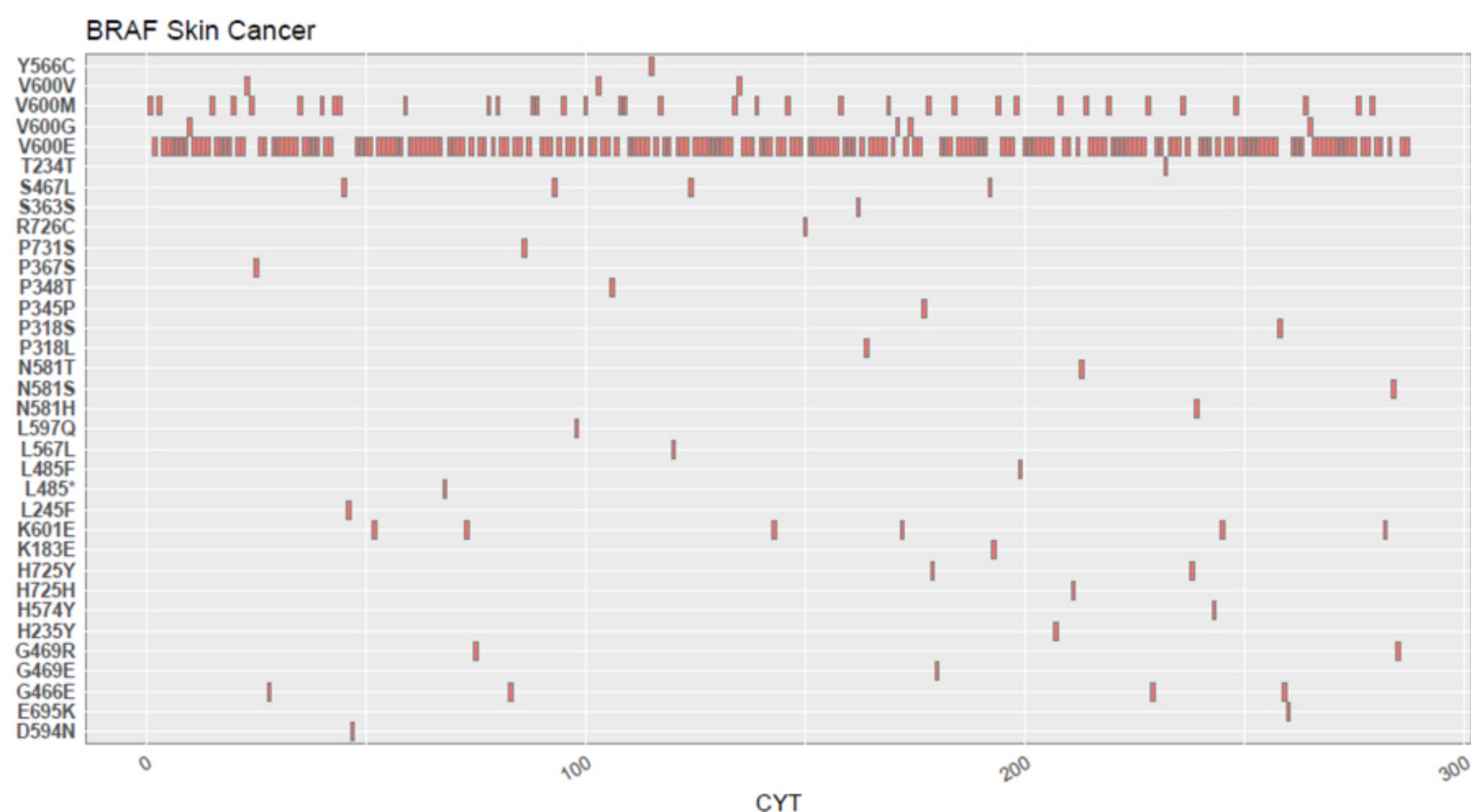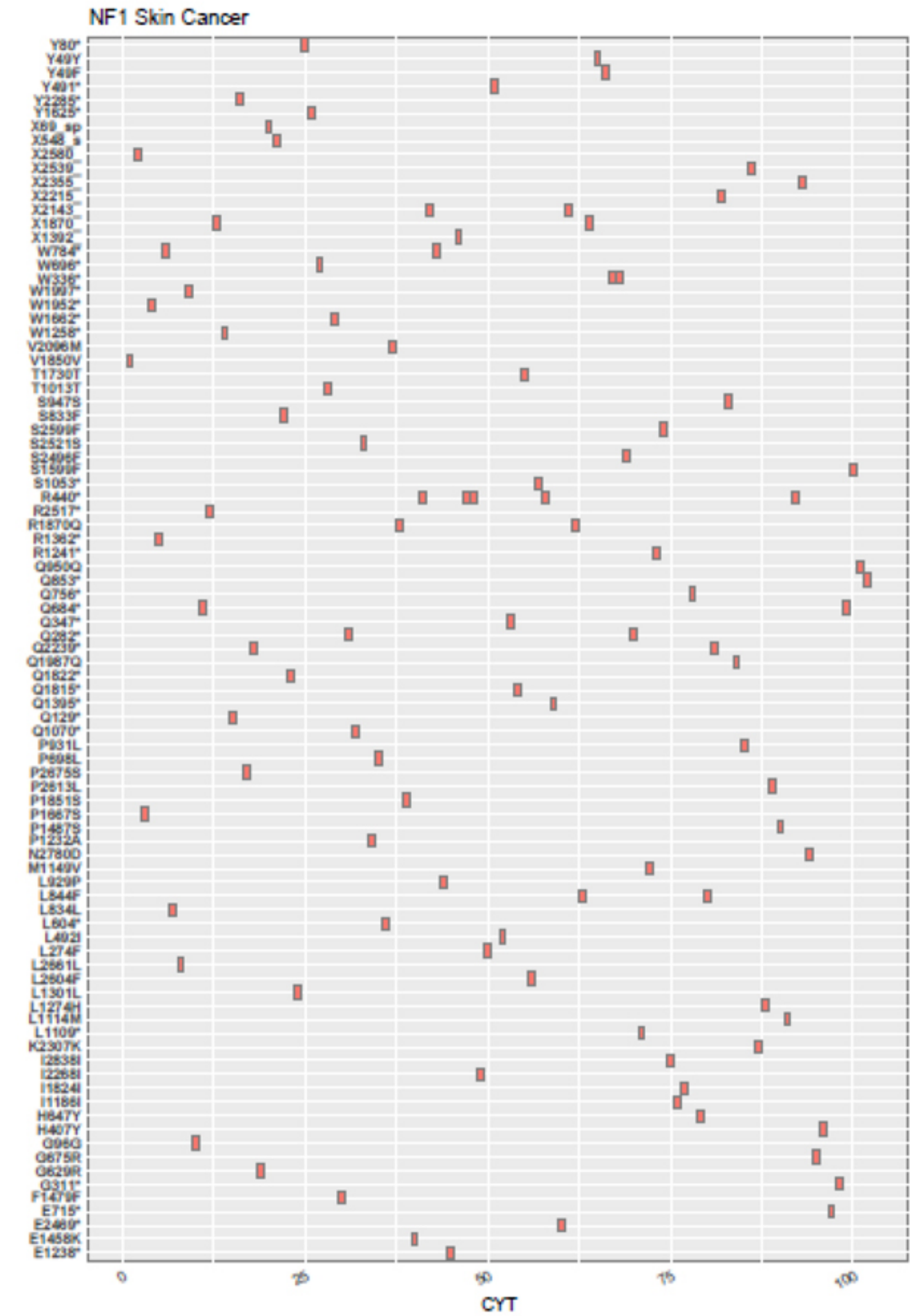

Supplement: Supplementary file 9 — Supplementary file9 (PDF 563 kb) [file 262_2021_2918_MOESM9_ESM.pdf]
