# Supplementary material for: Distinct genomic features across cytolytic subgroups in skin melanoma
Source: Cancer Immunol Immunother. 2021 Mar 29;70(11):3137–54. doi: 10.1007/s00262-021-02918-3 (PMC8505325; doi:10.1007/s00262-021-02918-3)
Supplement: Supplementary file 5 — Supplementary file5 (PDF 795 kb) [file 262_2021_2918_MOESM5_ESM.pdf]

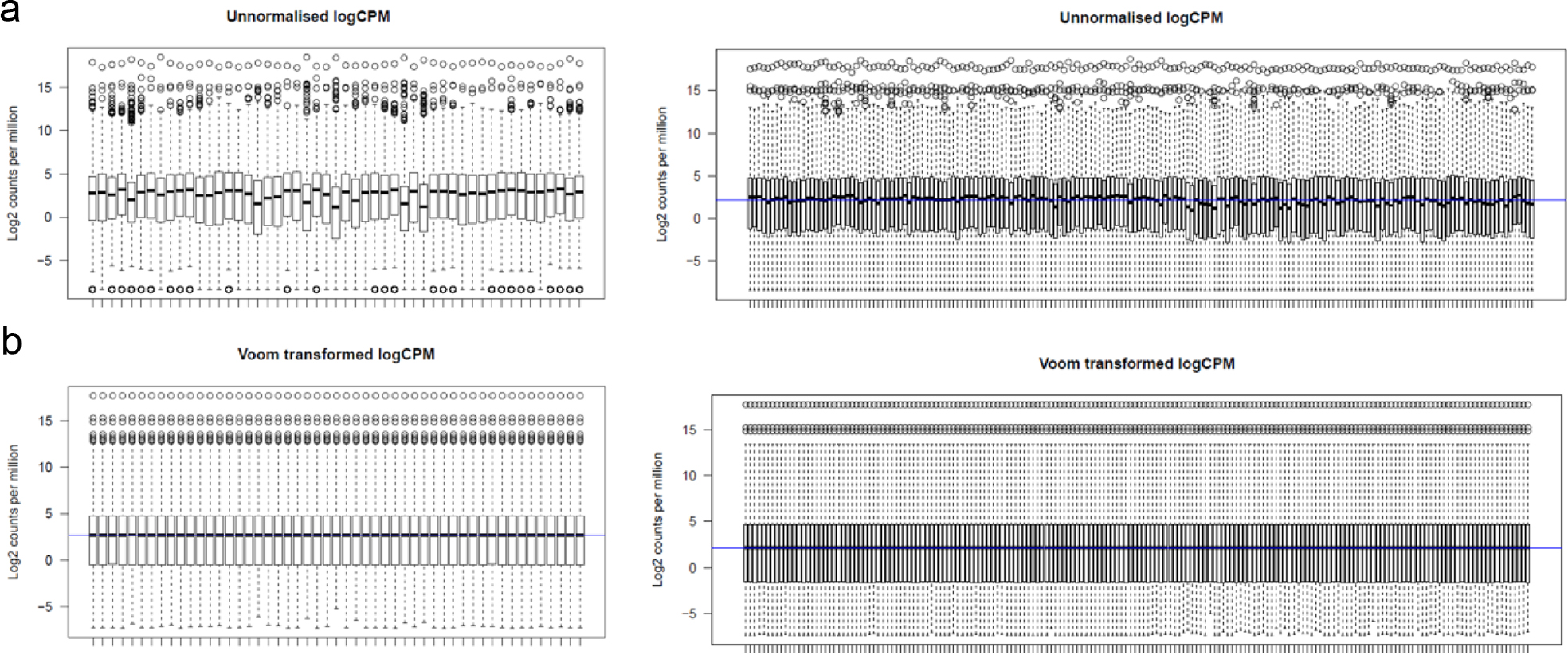
a
Unnormalised logCPM
Log2 counts per million
15
10
5
0
-5
Unnormalised logCPM
Log2 counts per million
15
10
5
0
-5
b
Voom transformed logCPM
Log2 counts per million
15
10
5
0
-5
Voom transformed logCPM
Log2 counts per million
15
10
5
0
-5
