# Supplementary material for: Distinct genomic features across cytolytic subgroups in skin melanoma
Source: Cancer Immunol Immunother. 2021 Mar 29;70(11):3137–54. doi: 10.1007/s00262-021-02918-3 (PMC8505325; doi:10.1007/s00262-021-02918-3)

CYT-high metastatic melanoma

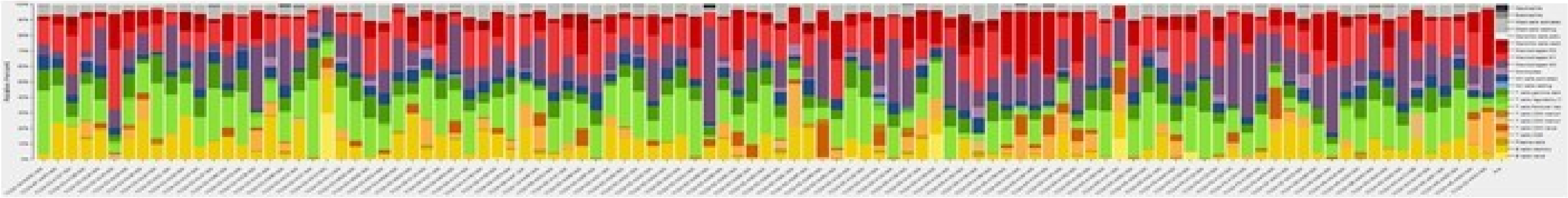

CYT-low metastatic melanoma

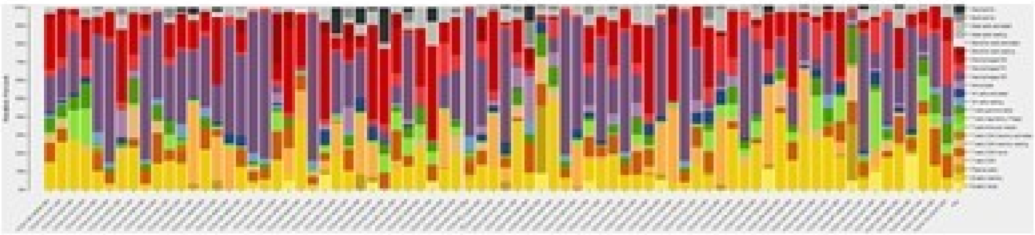

CYT-high primary melanoma

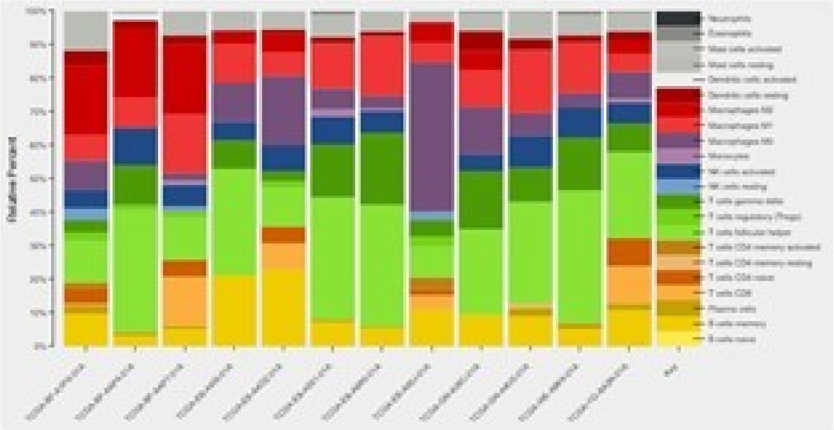

CYT-low primary melanoma

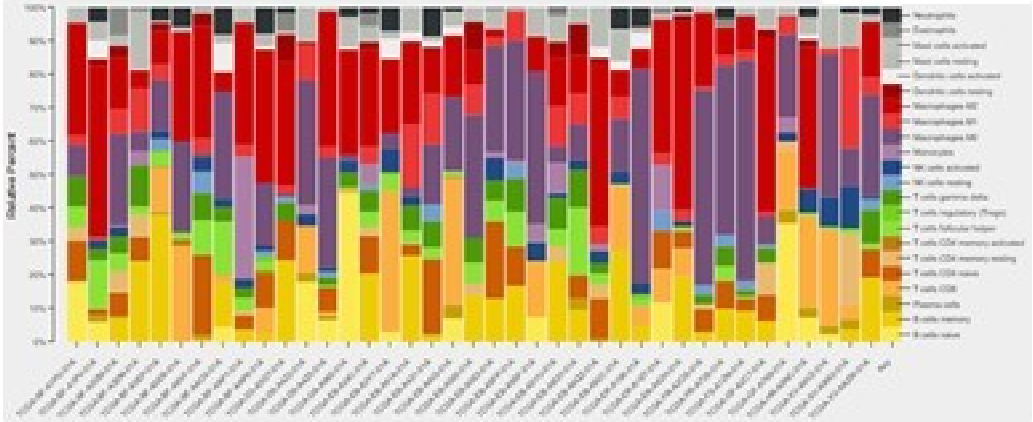

Supplement: Supplementary file 8 — Supplementary file8 (PDF 150 kb) [file 262_2021_2918_MOESM8_ESM.pdf]
