# Supplementary material for: Distinct genomic features across cytolytic subgroups in skin melanoma
Source: Cancer Immunol Immunother. 2021 Mar 29;70(11):3137–54. doi: 10.1007/s00262-021-02918-3 (PMC8505325; doi:10.1007/s00262-021-02918-3)
Supplement: Supplementary file 10 — Supplementary file10 (PDF 656 kb) [file 262_2021_2918_MOESM10_ESM.pdf]

gender	primary_location	breslow_thickness	clark_level	primary_melanoma_tumor_ulceration	ajcc_tumor_pathologic_pt	ajcc_nodes_pathologic_pn	ajcc_metastasis_pathologic_pm	submitted_tumor_site
MALE	Distant Metastasis	0.7	III	NO	T1a	NX	M0	Trunk
MALE	Regional Lymph Node	1.8	IV	YES	T2b	NX	M0	Trunk
MALE	Regional Lymph Node	1.25	III	NO	T2a	N1a	M0	Other Specify
FEMALE	Primary Tumor	13	III	YES	T4b	N0	M0	Extremities
FEMALE	Primary Tumor	9	IV	YES	T4b	N0	M0	Trunk
MALE	Primary Tumor	12	III	YES	T4b	N2a	M0	Trunk
FEMALE	Primary Tumor	8	III	NO	T4a	N0	M0	Extremities
MALE	Primary Tumor	5	III	YES	T4b	N0	M0	Trunk
FEMALE	Primary Tumor	11	III	YES	T4b	N1	M0	Extremities
FEMALE	Primary Tumor	3	IV	YES	T3b	N2	M0	Extremities
MALE	Primary Tumor	1.5	V	YES	T2b	N0	M0	Trunk
FEMALE	Primary Tumor	3	IV	YES	T3b	N3	M0	Extremities
MALE	Primary Tumor	8	III	YES	T4b	N0	M0	Trunk
FEMALE	Primary Tumor	70	V	YES	T4b	N3	M0	Trunk
MALE	Primary Tumor	5	III	NA	T4b	N0	M0	Trunk
MALE	Primary Tumor	14	IV	YES	T4b	N0	M0	Trunk
FEMALE	Primary Tumor	8	IV	YES	T4b	N0	M0	Trunk
MALE	Primary Tumor	12	III	YES	T4b	N0	M0	Extremities
FEMALE	Primary Tumor	12	IV	YES	T4b	N0	M0	Trunk
MALE	Primary Tumor	11	IV	YES	T4b	N0	M0	Extremities
FEMALE	Regional Lymph Node	NA	NA	NA	T4	NX	M1	Trunk
MALE	Primary Tumor	15	IV	YES	T4b	N0	M0	Extremities
MALE	Primary Tumor	3	III	YES	T3b	N0	M0	Extremities
MALE	Primary Tumor	15	V	YES	T4b	N0	M0	Trunk
MALE	Primary Tumor	20	IV	YES	T4b	N2	M0	Extremities
FEMALE	Primary Tumor	5	IV	YES	T4b	N0	M0	Extremities
MALE	Primary Tumor	6	IV	YES	T4b	N0	M0	Extremities
FEMALE	Regional Lymph Node	0.4	II	NO	T1b	N3	M0	Extremities
MALE	Regional Lymph Node	4.6	NA	YES	T4b	N0	M0	Extremities
FEMALE	Regional Lymph Node	1.45	IV	YES	T2b	N1b	M0	Extremities
MALE	Regional Lymph Node	NA	NA	NA	TX	N0	M0	Trunk

MALE	Regional Cutaneous or Subcutaneous Tissue (includes satellite and in-transit metastasis)		50	V	NA	T4	N1b	M0	Extremities
FEMALE	Regional Lymph Node		0.68	III	NO	T1b	N0	M0	Trunk
MALE	Regional Lymph Node	NA		NA	NA	T0	N3	M1b	NA
MALE	Regional Lymph Node		6	V	YES	T4b	N2a	M0	Extremities
MALE	Regional Lymph Node		1.12	III	NO	T2a	N0	M0	Extremities
FEMALE	Regional Lymph Node	NA		NA	NA	T0	N2c	M0	NA
MALE	Regional Lymph Node		3.2	IV	YES	T3b	N0	M0	Extremities
MALE	Regional Lymph Node		3.6	III	YES	T3b	N1b	M0	Trunk
MALE	Regional Lymph Node		1.4	IV	NO	T2a	N0	M0	Extremities
MALE	Regional Lymph Node		21	V	YES	T4b	N3	M0	Trunk|Extremities
MALE	Regional Cutaneous or Subcutaneous Tissue (includes satellite and in-transit metastasis)		1.2	III	NO	T2a	N1a	M0	Trunk
FEMALE	Regional Lymph Node	NA		NA	NA	Tis	N0	M0	Extremities
FEMALE	Regional Lymph Node	NA		NA	NA	T0	N2b	M0	NA
MALE	Regional Lymph Node		18	V	YES	T4b	N1b	M0	Extremities
FEMALE	Regional Lymph Node	NA		NA	NA	TX	N3	M0	Trunk
MALE	Distant Metastasis		0.28	III	NO	T1a	N0	M0	Extremities|Trunk
FEMALE	Regional Lymph Node		2.5	IV	NO	T3a	N1a	M0	Extremities
MALE	Regional Cutaneous or Subcutaneous Tissue (includes satellite and in-transit metastasis)		1	II	NO	T1b	N0	M0	Extremities
MALE	Regional Lymph Node		4.3	IV	YES	T4b	N2b	M0	Trunk
FEMALE	Regional Lymph Node	NA		NA	NA	TX	N0	M0	Extremities
FEMALE	Regional Lymph Node	NA		NA	NA	T0	N1b	M0	NA
FEMALE	Regional Cutaneous or Subcutaneous Tissue (includes satellite and in-transit metastasis)	NA		NA	YES	TX	N3	M0	Extremities
MALE	Regional Lymph Node	NA		NA	NA	T0	N3	M0	NA
MALE	Regional Lymph Node		4.6	V	NO	T4a	N0	M0	Head and Neck
MALE	Regional Lymph Node	NA		NA	NA	TX	N0	M0	Trunk
FEMALE	Regional Lymph Node	NA		NA	NA	TX	N0	M0	Trunk
FEMALE	Regional Lymph Node		1.3	IV	NO	T2a	N0	M0	Extremities
FEMALE	Regional Lymph Node	NA		NA	NA	T0	N1b	M0	NA

FEMALE	Regional Cutaneous or Subcutaneous Tissue (includes satellite and in-transit metastasis)	NA	NA	NA	TX	N3	M0	Head and Neck
MALE	Regional Lymph Node	1.75	III	NA	T2	N0	M0	Trunk
FEMALE	Regional Cutaneous or Subcutaneous Tissue (includes satellite and in-transit metastasis)	7.2	V	YES	T4b	N0	M0	Other Specify
FEMALE	Regional Lymph Node	NA	NA	NA	T0	N1b	M0	NA
FEMALE	Regional Lymph Node	9	V	YES	T4b	N3	M0	Other Specify
MALE	Regional Lymph Node	2.3	IV	NO	T3a	N2a	M0	Head and Neck
MALE	Regional Cutaneous or Subcutaneous Tissue (includes satellite and in-transit metastasis)	NA	NA	NA	TX	N2c	M0	Trunk
MALE	Regional Cutaneous or Subcutaneous Tissue (includes satellite and in-transit metastasis)	NA	NA	NA	T0	N1b	M0	NA
MALE	Regional Lymph Node	2.9	IV	NO	T3a	N2a	M0	Trunk
FEMALE	Regional Cutaneous or Subcutaneous Tissue (includes satellite and in-transit metastasis)	1.2	IV	YES	T2b	N2a	M0	Extremities
FEMALE	Regional Cutaneous or Subcutaneous Tissue (includes satellite and in-transit metastasis)	1.2	III	NA	T2	N0	M0	Extremities
MALE	Regional Lymph Node	7	IV	YES	T4b	N1b	M0	Extremities
MALE	Regional Cutaneous or Subcutaneous Tissue (includes satellite and in-transit metastasis)	0	I	NA	Tis	N0	M0	Trunk
MALE	Regional Lymph Node	0.91	IV	YES	T1b	N3	M0	Trunk
MALE	Regional Cutaneous or Subcutaneous Tissue (includes satellite and in-transit metastasis)	NA	NA	NA	T0	N1b	M0	NA
MALE	Regional Lymph Node	0	I	NA	Tis	N2b	M0	Extremities
FEMALE	Regional Lymph Node	NA	NA	NA	T0	N3	M1c	NA
MALE	Regional Lymph Node	2.5	IV	NO	T3a	N0	M0	Extremities
FEMALE	Regional Lymph Node	4.2	IV	YES	T4b	N1b	M0	Trunk
MALE	Regional Lymph Node	1.6	IV	NO	T2a	N0	M0	Extremities
FEMALE	Regional Lymph Node	0.6	III	NA	T1	N0	M0	Trunk
MALE	Distant Metastasis	4.3	V	NA	T4	N0	M0	Head and Neck
FEMALE	Regional Lymph Node	0.98	III	NA	T1b	N1	M0	Extremities
MALE	Regional Lymph Node	0.62	II	NO	T1b	N1b	M1c	Trunk

MALE	Primary Tumor	1.92	IV	YES	T2b	N3	M0	Trunk
MALE	Regional Lymph Node	0.48	III	NO	T1b	N0	M0	Extremities
MALE	Regional Lymph Node	3.5	IV	NO	T3a	N1b	M0	Extremities
MALE	Regional Lymph Node	NA	NA	NA	TX	N3	M0	NA
FEMALE	Regional Lymph Node	20	IV	YES	T4b	N3	M0	Extremities
MALE	Regional Cutaneous or Subcutaneous Tissue (includes satellite and in-transit metastasis)	NA	NA	NA	TX	N0	M1b	NA
MALE	Regional Lymph Node	0.98	IV	NO	T1a	N0	M0	Trunk
MALE	Distant Metastasis	2.4	III	NA	T3	N0	M0	Head and Neck
MALE	Distant Metastasis	2.6	IV	NO	T3a	N0	M0	Extremities
MALE	Regional Cutaneous or Subcutaneous Tissue (includes satellite and in-transit metastasis)	1.32	IV	NO	T2a	N1b	M0	Extremities
MALE	Regional Lymph Node	3.8	IV	YES	T3b	N0	M0	Trunk
FEMALE	Distant Metastasis	NA	NA	NA	TX	N0	M0	NA
FEMALE	Regional Lymph Node	1.2	III	NA	T2	N0	M0	Extremities
MALE	Distant Metastasis	1.8	IV	NA	T2	N2c	M0	Extremities
MALE	Distant Metastasis	2.6	III	NA	T3	N0	M0	Trunk
FEMALE	Distant Metastasis	0.01	I	NA	Tis	N0	M0	Extremities
MALE	Distant Metastasis	0.6	III	NA	T1	N0	M0	Trunk
MALE	Distant Metastasis	NA	NA	NA	TX	N0	M0	Extremities
MALE	Regional Lymph Node	NA	NA	NO	TX	N3	M1b	Trunk
FEMALE	Regional Cutaneous or Subcutaneous Tissue (includes satellite and in-transit metastasis)	NA	NA	NO	TX	N1b	M0	Trunk
MALE	Regional Lymph Node	NA	NA	NO	T1a	N2a	M0	Head and Neck
FEMALE	Regional Lymph Node	NA	III	NO	TX	NX	M0	Extremities
MALE	Regional Cutaneous or Subcutaneous Tissue (includes satellite and in-transit metastasis)	12	V	YES	T4b	N2b	NA	Extremities
MALE	Regional Lymph Node	1.7	IV	NA	T2a	N3	M0	NA
FEMALE	Regional Lymph Node	3.9	IV	NO	T3a	N1b	M0	NA
MALE	Primary Tumor	12	V	YES	T4b	N3	M0	NA
MALE	Primary Tumor	25	IV	YES	T4b	N3	M0	Trunk|Extremities
FEMALE	Primary Tumor	75	V	YES	T4b	N1b	M0	Extremities

MALE	NA	NA	III	NO	T4a	N0	M0	Head and Neck
MALE	Regional Lymph Node	NA	NA	NA	T3b	N1b	M0	Trunk
FEMALE	Primary Tumor	4	IV	NO	T3a	N1	M0	Extremities
MALE	[Discrepancy]	6	IV	NO	T4a	N3	M0	Trunk
MALE	Regional Lymph Node	3	III	NO	T3a	N1	M0	Trunk
MALE	Regional Cutaneous or Subcutaneous Tissue (includes satellite and in-transit metastasis)	5	IV	YES	T4a	N1	M0	Trunk
FEMALE	Regional Lymph Node	NA	NA	NA	T0	N2b	M0	NA
FEMALE	Regional Lymph Node	0.7	III	NO	T1a	N1b	M0	Trunk
MALE	Regional Lymph Node	1.13	IV	YES	T2b	N1	M0	Extremities
MALE	Regional Cutaneous or Subcutaneous Tissue (includes satellite and in-transit metastasis)	4.5	IV	YES	T4b	N3	M1a	Trunk
MALE	Regional Cutaneous or Subcutaneous Tissue (includes satellite and in-transit metastasis)	NA	NA	NA	T0	N2a	M0	NA
MALE	Regional Lymph Node	NA	IV	YES	T4b	N2b	M0	Trunk
MALE	Regional Cutaneous or Subcutaneous Tissue (includes satellite and in-transit metastasis)	3.4	IV	YES	T3b	N2b	M0	Trunk
FEMALE	Distant Metastasis	0.6	IV	NO	T1a	N0	M1c	Head and Neck
MALE	Regional Cutaneous or Subcutaneous Tissue (includes satellite and in-transit metastasis)	NA	NA	NA	T0	N2b	M0	NA
FEMALE	Regional Cutaneous or Subcutaneous Tissue (includes satellite and in-transit metastasis)	8	IV	YES	T4b	N0	M0	Extremities
FEMALE	Distant Metastasis	1.3	IV	NO	T2a	N1b	M0	Extremities
FEMALE	Regional Lymph Node	1.5	IV	YES	T2b	N2b	M0	Extremities
MALE	Regional Cutaneous or Subcutaneous Tissue (includes satellite and in-transit metastasis)	2.5	IV	NO	T3a	N2c	M0	Extremities
MALE	Regional Cutaneous or Subcutaneous Tissue (includes satellite and in-transit metastasis)	5.1	V	NO	T4a	N2b	M0	Trunk
MALE	Regional Lymph Node	NA	NA	NA	T0	N2b	M0	NA
MALE	Regional Lymph Node	0.4	III	NA	T1a	N0	M0	Trunk
MALE	Regional Lymph Node	1.9	IV	NO	T2a	N2b	M0	Head and Neck
FEMALE	Regional Lymph Node	4.1	IV	YES	T4b	N0	NA	Head and Neck

MALE	Regional Lymph Node	NA	NA	NA	TX	N1b	M0	NA
MALE	Regional Lymph Node	1.45	IV	NO	T2a	N0	M0	Extremities
MALE	Regional Lymph Node	5.03	IV	YES	T4b	N0	M0	Trunk
MALE	Regional Cutaneous or Subcutaneous Tissue (includes satellite and in-transit metastasis)	NA	NA	NA	TX	N0	M1a	NA
MALE	Regional Cutaneous or Subcutaneous Tissue (includes satellite and in-transit metastasis)	2.32	IV	YES	T3b	N0	M0	Trunk
MALE	Primary Tumor	8	IV	YES	T4b	N0	M0	Extremities
MALE	Primary Tumor	10	NA	YES	T4b	NX	M0	Trunk
MALE	Primary Tumor	36	NA	NO	T4a	N2b	M0	Extremities
MALE	Primary Tumor	2	III	YES	T2b	N0	M0	Extremities
MALE	Primary Tumor	13	IV	YES	T4b	N0	M0	Trunk
MALE	Primary Tumor	NA	IV	NA	T4	NX	M0	Trunk
MALE	Primary Tumor	NA	NA	YES	T4b	N0	M0	Trunk
FEMALE	Primary Tumor	NA	NA	NA	T4b	NX	M0	Extremities
FEMALE	Primary Tumor	3	NA	NO	T3a	N0	M0	Trunk
MALE	Primary Tumor	10	NA	YES	T4b	N0	M0	Trunk
FEMALE	Primary Tumor	4.5	NA	YES	T4b	N0	M0	Extremities
FEMALE	Primary Tumor	4	NA	NO	T3a	N2c	M0	Head and Neck
MALE	Primary Tumor	7	NA	YES	T4b	N0	M0	Head and Neck
FEMALE	Primary Tumor	28	V	YES	T4b	N0	M0	Extremities
FEMALE	Primary Tumor	5	NA	YES	T4b	N0	M0	Extremities
MALE	Primary Tumor	10	NA	YES	T4b	N1b	M0	Extremities
MALE	Primary Tumor	18	IV	YES	T4b	N0	M0	Trunk
MALE	Primary Tumor	15	III	YES	T4b	N0	M0	Extremities
MALE	Primary Tumor	5	III	YES	T4b	N0	M0	Extremities
MALE	Primary Tumor	5	NA	NO	T4a	N0	M0	Trunk
FEMALE	Primary Tumor	NA	NA	NO	T4b	N0	M0	Trunk
FEMALE	Regional Lymph Node	NA	NA	YES	TX	N3	M0	Extremities
MALE	Regional Lymph Node	NA	NA	NA	TX	N2b	M0	Extremities
FEMALE	Primary Tumor	15	NA	YES	T4b	N1	M0	Trunk
MALE	Primary Tumor	2.5	NA	YES	T3b	NX	M0	Trunk

FEMALE	Primary Tumor	10	V	NA	T4b	N1a	M0	Extremities
FEMALE	Primary Tumor	18	V	NO	T4a	N3	M0	Trunk
MALE	Primary Tumor	8	IV	YES	T4b	N0	M0	Trunk
FEMALE	Primary Tumor	7	IV	YES	T4b	NX	M0	Extremities
MALE	Primary Tumor	10	III	YES	T4b	NX	M0	Trunk
FEMALE	Primary Tumor	15	IV	YES	T4b	NX	M0	NA
FEMALE	Primary Tumor	10	V	YES	T4b	N2b	M0	Extremities
MALE	Primary Tumor	10	NA	YES	T4b	N0	M0	Trunk
MALE	Primary Tumor	8	NA	YES	T4b	N1	M0	Extremities
FEMALE	Primary Tumor	8	NA	YES	T4b	NX	M1b	Trunk
MALE	Regional Lymph Node	NA	NA	NA	T0	N1	M0	Extremities
MALE	Primary Tumor	4	IV	YES	T3b	NX	M0	Trunk
FEMALE	Primary Tumor	11	V	YES	T4b	NX	M0	Extremities
FEMALE	Regional Lymph Node	NA	NA	NO	NA	NA	NA	Extremities
FEMALE	Regional Lymph Node	5	V	YES	T4	N0	M0	Extremities
MALE	Regional Lymph Node	4	NA	NA	TX	N1	M0	Trunk
FEMALE	Primary Tumor	5	NA	YES	T4b	N0	M0	Trunk
MALE	Distant Metastasis	14	IV	YES	T4b	NX	M0	Trunk
MALE	Primary Tumor	15	NA	YES	T4b	N1	M0	Extremities
FEMALE	Regional Lymph Node	4	NA	YES	T3b	N1	M0	Head and Neck
MALE	Regional Lymph Node	NA	NA	YES	TX	N3	M0	Extremities
MALE	Primary Tumor	10	NA	YES	T4b	N0	M0	Trunk
FEMALE	Primary Tumor	3	NA	NO	T3a	N0	M0	Trunk
FEMALE	Primary Tumor	10	IV	YES	T4b	N0	M0	Trunk
FEMALE	Primary Tumor	20	V	YES	T4b	N2	M0	Extremities
FEMALE	Primary Tumor	10	V	YES	T4b	N0	M0	Extremities
MALE	Primary Tumor	10	NA	YES	T4b	N0	M0	Trunk
FEMALE	Primary Tumor	5.5	NA	NO	T4a	N0	M0	Trunk
MALE	Primary Tumor	15	IV	YES	T4b	N0	M0	Trunk
MALE	Regional Lymph Node	0.8	III	NO	T1a	N0	M0	Trunk
MALE	Regional Cutaneous or Subcutaneous Tissue (includes satellite and in-transit metastasis)	2.8	IV	YES	T3b	N1a	M0	Trunk

MALE	Regional Lymph Node		29	V	NO	T4a	N0	M0	Trunk
MALE	Distant Metastasis		4.9	IV	NO	T4a	N0	M0	Trunk
FEMALE	Regional Lymph Node		2.5	IV	NA	T3	N0	M0	Extremities
FEMALE	Distant Metastasis		14	V	YES	T4b	N1b	M0	Trunk
MALE	Regional Lymph Node	NA		I	NA	Tis	N0	M0	Trunk
MALE	Distant Metastasis		1.95	IV	NO	T2a	N0	M0	Extremities
FEMALE	Regional Lymph Node		6	IV	YES	T4b	N3	M0	Extremities
FEMALE	Regional Cutaneous or Subcutaneous Tissue (includes satellite and in-transit metastasis)		2.6	III	NA	T3	N0	M0	Extremities
MALE	Distant Metastasis	NA		I	NA	Tis	N0	M0	Extremities
MALE	Regional Lymph Node		0.5	NA	NA	T1	N0	M0	Extremities
MALE	Distant Metastasis		1.05	III	NA	T2	N0	M0	Extremities
MALE	Regional Cutaneous or Subcutaneous Tissue (includes satellite and in-transit metastasis)	NA		NA	YES	TX	N0	M1c	Extremities
MALE	Distant Metastasis		2.3	IV	NO	T3a	N1a	M0	Trunk
MALE	Distant Metastasis		4	III	YES	T3b	N0	M0	Extremities
MALE	Regional Lymph Node		1.7	IV	NO	T2a	N0	M0	Trunk
MALE	Regional Lymph Node		3.2	IV	YES	T3b	N1b	M0	Head and Neck
MALE	Regional Lymph Node		3.2	IV	NO	T3a	N1b	M0	Extremities
MALE	Regional Cutaneous or Subcutaneous Tissue (includes satellite and in-transit metastasis)		7.4	V	NO	T4a	N2a	M0	Head and Neck
FEMALE	Regional Lymph Node		1	II	NO	T1a	N0	M0	Trunk
MALE	Regional Lymph Node		7	V	YES	T4b	N3	M0	Extremities
FEMALE	Regional Lymph Node		1.02	III	NO	T2a	N0	M0	Extremities
MALE	Regional Lymph Node	NA		NA	NA	TX	N0	M0	Trunk
FEMALE	Regional Lymph Node		4.5	IV	YES	T4b	N0	M0	Trunk
FEMALE	Regional Cutaneous or Subcutaneous Tissue (includes satellite and in-transit metastasis)		2.3	IV	YES	T3b	N0	M0	Extremities
FEMALE	Regional Lymph Node		3.5	V	YES	T3b	N1b	M0	Head and Neck
MALE	Regional Lymph Node		2.5	IV	NO	T3a	N0	M0	Extremities
FEMALE	Regional Lymph Node	NA		NA	NA	TX	NX	M0	Trunk
MALE	Distant Metastasis		3.5	IV	YES	T3b	N1b	M0	Head and Neck

MALE	Regional Lymph Node	0	I	NA	Tis	N0	M0	Extremities
FEMALE	Regional Lymph Node	2	IV	NO	T2a	N0	M0	Head and Neck
FEMALE	Regional Lymph Node	2.2	IV	NO	T3a	N0	M0	Extremities
MALE	Regional Lymph Node	1.3	IV	NO	T2a	N0	M0	Extremities
MALE	Regional Lymph Node	7	IV	YES	T4b	N1b	M0	Trunk
MALE	Regional Cutaneous or Subcutaneous Tissue (includes satellite and in-transit metastasis)	1.1	IV	NO	T2a	N0	M0	Extremities
MALE	Regional Lymph Node	0.55	II	NO	T1a	N0	M0	Trunk
MALE	Regional Lymph Node	1.65	III	YES	T2b	N1a	M0	Trunk
MALE	Regional Lymph Node	2.3	III	YES	T3b	N0	M0	Head and Neck
FEMALE	Regional Cutaneous or Subcutaneous Tissue (includes satellite and in-transit metastasis)	4.4	IV	YES	T4	N0	M0	Extremities
MALE	Regional Lymph Node	0.9	III	NO	T2	N0	M0	Trunk
MALE	Regional Lymph Node	0.8	III	NA	T2	N0	M0	Trunk
MALE	Regional Lymph Node	0.65	III	NO	T1a	N0	M0	Extremities
MALE	Regional Lymph Node	0.4	II	NO	T1a	N0	M0	Extremities
FEMALE	Regional Lymph Node	0.8	III	NA	T1	N0	M0	Trunk
FEMALE	Regional Lymph Node	3.1	IV	NO	T3a	N0	M0	Extremities
FEMALE	Regional Cutaneous or Subcutaneous Tissue (includes satellite and in-transit metastasis)	6.5	IV	YES	T4b	N0	M0	Trunk
MALE	Regional Cutaneous or Subcutaneous Tissue (includes satellite and in-transit metastasis)	1.9	IV	YES	T2b	N0	M0	Trunk
FEMALE	Regional Cutaneous or Subcutaneous Tissue (includes satellite and in-transit metastasis)	3.4	IV	NO	T3b	N0	M0	Extremities
MALE	Regional Cutaneous or Subcutaneous Tissue (includes satellite and in-transit metastasis)	4.2	IV	YES	T4b	N1a	M0	Extremities
MALE	Regional Cutaneous or Subcutaneous Tissue (includes satellite and in-transit metastasis)	6.9	IV	NA	T4	N0	M0	Extremities|Extremities
FEMALE	Regional Cutaneous or Subcutaneous Tissue (includes satellite and in-transit metastasis)	1.8	IV	NO	T2a	N0	M0	Extremities
MALE	Regional Lymph Node	2.2	IV	NO	T3a	N0	M0	Trunk
FEMALE	Regional Lymph Node	0.3	II	NO	T1a	N0	M0	Extremities

MALE	Regional Lymph Node		0.8	III	NO	T2	N0	M0	Trunk
MALE	Regional Lymph Node		0.7	II	NO	T1	N0	M0	Trunk
MALE	Regional Lymph Node		2.2	III	NA	T3a	N0	M0	Trunk
FEMALE	Regional Lymph Node		2	IV	NO	T3a	N1	M0	Extremities
MALE	Regional Lymph Node		1	III	NO	T2	N0	M0	Extremities
MALE	Regional Lymph Node		3	III	YES	T3a	N0	M0	Extremities
MALE	Regional Lymph Node		0.52	III	NO	T2	N0	M0	Trunk
FEMALE	Regional Lymph Node		1.5	IV	NA	T2	N0	M0	Extremities
MALE	Regional Lymph Node		0.7	III	NA	T2	N0	M0	Trunk
MALE	Regional Lymph Node		3.4	V	NO	T4a	N0	M0	Trunk
MALE	Regional Lymph Node		0.4	II	NA	T4	N0	M0	Extremities
MALE	Regional Lymph Node		11	V	NO	T4b	N0	M0	Trunk
FEMALE	Regional Lymph Node		5.2	IV	NO	T4a	N0	M0	Extremities
MALE	Regional Lymph Node		3	III	NO	T3a	N0	M0	Trunk
FEMALE	Regional Lymph Node		0.6	III	NA	T2	N0	M0	Trunk
MALE	Regional Lymph Node		1	III	NO	T2	N0	M0	Trunk
FEMALE	Regional Lymph Node		1.05	NA	NA	T2	N0	M0	Extremities
FEMALE	Regional Lymph Node		3.1	IV	NO	T3a	N2a	M0	Trunk
MALE	Regional Lymph Node		1.25	III	NO	T2	N0	M0	Trunk
MALE	Regional Lymph Node		1.8	IV	NO	T3a	N0	M0	Extremities
MALE	Regional Lymph Node		1.5	IV	NO	T2a	N0	M0	Trunk
MALE	Regional Cutaneous or Subcutaneous Tissue (includes satellite and in-transit metastasis)		0.5	II	NO	T1a	N0	M0	Extremities
MALE	Regional Lymph Node	NA		NA	NA	T0	N2a	M0	NA
MALE	Regional Lymph Node	NA		NA	NA	T0	N2a	M0	NA
MALE	Regional Lymph Node	NA		NA	NA	T0	N2b	M0	NA
MALE	Regional Lymph Node	NA		NA	NA	T0	N1b	M0	NA
FEMALE	Regional Lymph Node		0.9	III	NO	T1a	N0	M0	Extremities
FEMALE	Regional Lymph Node	NA		NA	NA	T0	N3	M0	NA
MALE	Regional Cutaneous or Subcutaneous Tissue (includes satellite and in-transit metastasis)	NA		NA	NA	T0	N2c	M0	NA
MALE	Regional Lymph Node		3.7	III	NO	T3b	N0	M0	Extremities

MALE	Regional Cutaneous or Subcutaneous Tissue (includes satellite and in-transit metastasis)		0.98	NA	NA	T2	N0	M0	Trunk
MALE	Regional Cutaneous or Subcutaneous Tissue (includes satellite and in-transit metastasis)		2	IV	YES	T3a	N0	M0	Extremities
MALE	Regional Cutaneous or Subcutaneous Tissue (includes satellite and in-transit metastasis)		4.8	V	NO	T4a	N1	M0	Extremities
MALE	Regional Cutaneous or Subcutaneous Tissue (includes satellite and in-transit metastasis)		1.12	II	YES	T2	N0	M0	Trunk
MALE	Regional Cutaneous or Subcutaneous Tissue (includes satellite and in-transit metastasis)		4.8	IV	NO	T4a	N1a	M0	Trunk
MALE	Regional Lymph Node		1.5	IV	NO	T2a	N0	M0	Extremities
FEMALE	Regional Cutaneous or Subcutaneous Tissue (includes satellite and in-transit metastasis)		1.86	IV	NA	T3a	N1	M0	Extremities
MALE	Regional Lymph Node	NA		NA	NA	TX	N2b	M0	NA
MALE	Regional Cutaneous or Subcutaneous Tissue (includes satellite and in-transit metastasis)		3.7	IV	YES	T3b	N1a	M0	Trunk
MALE	Regional Lymph Node		1.3	III	NA	T2	N0	M0	Trunk
MALE	Regional Lymph Node		1.2	IV	NO	T2	N0	M0	Extremities|Trunk
MALE	Regional Lymph Node	NA		NA	NO	T3b	N0	M0	Head and Neck
MALE	Primary Tumor		4.5	IV	YES	NA	N0	M0	Other Specify
MALE	Regional Lymph Node	NA		NA	NA	TX	N0	M0	Trunk
FEMALE	Primary Tumor		22	IV	YES	T4b	N0	M0	Extremities
FEMALE	NA		8	NA	YES	T4b	N1a	M0	Other Specify
MALE	Regional Cutaneous or Subcutaneous Tissue (includes satellite and in-transit metastasis)		10	NA	YES	NA	NX	M0	Other Specify
FEMALE	Regional Lymph Node		3	IV	YES	T4b	N3	M0	Extremities
MALE	Regional Cutaneous or Subcutaneous Tissue (includes satellite and in-transit metastasis)	NA		NA	NA	TX	N0	M1	NA
MALE	Regional Cutaneous or Subcutaneous Tissue (includes satellite and in-transit metastasis)	NA		NA	NA	TX	N0	M0	Trunk
MALE	Regional Lymph Node		0.75	III	NO	T2a	NX	M0	Trunk
FEMALE	Regional Cutaneous or Subcutaneous Tissue		1.75	IV	NO	T2a	N0	M0	Trunk

	(includes satellite and<br>in-transit metastasis)							
FEMALE	Regional Lymph Node	1.1	III	NO	T2a	N0	M0	Extremities
MALE	Regional Lymph Node	6.7	NA	NA	NA	N0	M0	Extremities
FEMALE	Regional Lymph Node	1.6	II	NO	NA	N0	M0	Extremities
MALE	Regional Cutaneous or<br>Subcutaneous Tissue<br>(includes satellite and<br>in-transit metastasis)	0.4	II	NA	NA	N0	M0	Trunk
MALE	Regional Lymph Node	NA	NA	NA	TX	N3	M1	NA
FEMALE	Primary Tumor	6.8	V	YES	T4b	N0	M0	Head and Neck
MALE	Regional Lymph Node	NA	NA	NA	NA	NA	NA	Extremities
MALE	Regional Lymph Node	1.9	IV	NO	T2a	N0	M0	Extremities
MALE	Regional Lymph Node	NA	NA	NA	NA	NA	NA	NA
MALE	Regional Lymph Node	NA	NA	NA	T3b	N1b	M0	Trunk
FEMALE	Regional Lymph Node	NA	IV	NA	NA	N0	M0	Extremities
FEMALE	Regional Lymph Node	1.2	III	NO	NA	N0	M0	Extremities
FEMALE	Regional Lymph Node	NA	NA	NA	NA	NA	NA	NA
MALE	Primary Tumor	15	V	NO	T4a	N3	M1a	Extremities
FEMALE	Regional Cutaneous or<br>Subcutaneous Tissue<br>(includes satellite and<br>in-transit metastasis)	0.75	II	NO	NA	NA	NA	Trunk
MALE	Regional Lymph Node	NA	NA	NA	TX	N3	M0	NA
MALE	Primary Tumor	4.39	IV	YES	T4b	N2	M0	Other Specify
MALE	Distant Metastasis	1.97	NA	NO	T2a	N0	M0	Extremities
FEMALE	Distant Metastasis	1	IV	NO	T1b	N3	M0	Extremities
MALE	Distant Metastasis	NA	NA	NA	Tis	N0	M0	Extremities
MALE	Primary Tumor	4	V	YES	T3b	N3	M0	Head and Neck
FEMALE	Regional Lymph Node	3	III	YES	T3b	N3	M0	Trunk
MALE	Regional Lymph Node	4	IV	NO	T3a	N3	M0	Trunk
MALE	Distant Metastasis	NA	NA	NA	NA	NA	NA	Extremities
FEMALE	Regional Cutaneous or<br>Subcutaneous Tissue<br>(includes satellite and<br>in-transit metastasis)	3.55	IV	NA	T3a	N1a	M0	Other Specify
MALE	Distant Metastasis	4.4	NA	NA	T4	N0	M0	Extremities
MALE	Regional Cutaneous or<br>Subcutaneous Tissue<br>(includes satellite and<br>in-transit metastasis)	2.2	V	YES	T3b	N0	M1a	Extremities

MALE	Primary Tumor	NA	NA	NA	NA	NA	NA	Other Specify
FEMALE	Regional Lymph Node	5.5	IV	YES	T4b	N3	M0	Extremities
MALE	Distant Metastasis	1.46	IV	NO	T3	N0	M0	Other Specify
FEMALE	Primary Tumor	NA	V	YES	T4b	N0	M0	Extremities
MALE	Primary Tumor	6.3	V	YES	T4b	N0	M0	Extremities
MALE	Regional Lymph Node	1.25	IV	NO	T2a	N0	M0	Extremities
FEMALE	Distant Metastasis	NA	NA	NA	T2	N0	M0	Extremities
FEMALE	Regional Lymph Node	2.51	IV	NO	T3a	N0	M0	Extremities
FEMALE	Regional Lymph Node	NA	NA	NA	TX	N3	NA	NA
MALE	Primary Tumor	14	IV	YES	T4b	N0	M0	Head and Neck
FEMALE	Primary Tumor	12	IV	YES	T4b	N2a	M0	Extremities
FEMALE	Regional Lymph Node	0.25	II	NO	T1	N0	M0	Trunk
MALE	Distant Metastasis	NA	NA	NA	TX	N3	M0	NA
FEMALE	Regional Lymph Node	2.8	IV	YES	T3b	N3	M0	Extremities
FEMALE	Regional Lymph Node	1.02	IV	NO	T2a	N0	M0	Extremities
MALE	Distant Metastasis	2.6	IV	YES	T3b	N0	M0	Extremities
FEMALE	Distant Metastasis	4.5	IV	YES	T4b	N0	M0	Other Specify
MALE	Regional Lymph Node	0.79	III	NO	T1a	N0	M0	Trunk
MALE	Regional Lymph Node	1	IV	NO	T1b	N0	M0	Trunk
FEMALE	Regional Cutaneous or Subcutaneous Tissue (includes satellite and in-transit metastasis)	1.1	IV	NO	T2	N0	M0	Trunk
FEMALE	Distant Metastasis	2.7	IV	NO	T3a	N0	M0	Trunk
FEMALE	Distant Metastasis	0.95	III	NO	T1a	N0	M0	Extremities
FEMALE	Distant Metastasis	NA	NA	NA	TX	N0	M1	NA
MALE	Regional Lymph Node	0.85	III	NO	T1	N0	M0	Extremities
MALE	Regional Lymph Node	17	V	YES	T4b	N1b	M0	Head and Neck
FEMALE	Distant Metastasis	4.5	IV	YES	T4b	N2c	M0	Trunk
MALE	Distant Metastasis	3	II	NO	T3a	N0	M0	Trunk
MALE	Distant Metastasis	NA	III	NA	TX	N0	M0	Trunk
MALE	Regional Lymph Node	1.18	III	YES	T2b	N0	M0	Extremities
MALE	Distant Metastasis	5	III	YES	T4b	N0	M0	Trunk
FEMALE	Distant Metastasis	4.3	IV	YES	T4b	N0	M0	Extremities

FEMALE	Regional Lymph Node		6	V	YES	T4b	N2b	M0	Extremities
FEMALE	Regional Lymph Node		3	IV	YES	T3b	N2c	M1c	Extremities
FEMALE	Regional Lymph Node		1.22	IV	NO	T2	N0	M0	Trunk
MALE	Distant Metastasis		5	IV	NA	T4	N0	M0	Head and Neck
MALE	Regional Cutaneous or Subcutaneous Tissue (includes satellite and in-transit metastasis)		1.2	IV	NA	T2	N2c	M0	Extremities
MALE	Primary Tumor		8	IV	YES	T4b	N1a	M0	Trunk
MALE	Regional Lymph Node		2.5	III	NA	T3	N0	M0	Extremities
MALE	Distant Metastasis	NA		NA	NA	TX	N0	M0	Extremities
MALE	Regional Cutaneous or Subcutaneous Tissue (includes satellite and in-transit metastasis)		2	IV	NA	T2	N0	M0	Trunk
MALE	Regional Lymph Node		1.15	NA	NA	T2	N0	M0	Trunk
MALE	Distant Metastasis		1.15	IV	NA	T2	N1b	M0	Extremities
FEMALE	Distant Metastasis		8.4	V	YES	T4b	N0	M0	Extremities
MALE	Regional Lymph Node		1.2	III	YES	T2b	N1a	M0	Extremities
MALE	Distant Metastasis		2.9	IV	YES	T3b	N0	M0	Trunk
FEMALE	Distant Metastasis		3.8	IV	YES	T3b	N0	M0	Trunk
FEMALE	Regional Cutaneous or Subcutaneous Tissue (includes satellite and in-transit metastasis)		14	V	NO	T4a	N0	M0	Trunk
FEMALE	Regional Cutaneous or Subcutaneous Tissue (includes satellite and in-transit metastasis)		4.7	V	YES	T4b	N0	M0	Head and Neck
MALE	Regional Cutaneous or Subcutaneous Tissue (includes satellite and in-transit metastasis)		2	IV	YES	T3a	N0	M0	Extremities
FEMALE	Regional Cutaneous or Subcutaneous Tissue (includes satellite and in-transit metastasis)		1.48	IV	NO	T2a	N0	M0	Extremities
MALE	Regional Lymph Node		0.9	NA	NA	T1	N0	M0	Extremities
MALE	Regional Lymph Node		10	NA	YES	T4b	N3	M0	Trunk
FEMALE	Regional Lymph Node		2	IV	NA	T2	N1a	M0	Extremities
FEMALE	Regional Cutaneous or Subcutaneous Tissue (includes satellite and in-transit metastasis)		4	IV	NO	T3a	N0	M0	Extremities
MALE	Regional Lymph Node		2	IV	NA	T2	N3	M0	Trunk|NA
FEMALE	Distant Metastasis	NA		NA	NA	NA	N0	M1	NA

MALE	Regional Lymph Node	NA		NA	NA	TX	N2	M0	Head and Neck
FEMALE	Distant Metastasis	NA		IV	NA	NA	NA	NA	Trunk
FEMALE	Regional Lymph Node		0.5	III	NA	T1	N2b	M0	Trunk
MALE	Primary Tumor		7	NA	NO	T4a	N3	NA	Trunk
FEMALE	Regional Lymph Node	NA		NA	NA	T3	N1	NA	Trunk
MALE	Primary Tumor	NA		IV	YES	T4b	N0	M0	Head and Neck
FEMALE	Regional Lymph Node	NA		NA	NA	T3	N3	M0	Extremities
FEMALE	Regional Lymph Node	NA		NA	NA	T0	N3	M0	NA
FEMALE	Regional Lymph Node		4	IV	YES	T3b	NX	M0	Extremities
MALE	Distant Metastasis	NA		NA	NA	NA	NA	NA	NA
MALE	Primary Tumor		74	IV	YES	T4b	NX	M0	Extremities
FEMALE	Regional Cutaneous or Subcutaneous Tissue (includes satellite and in-transit metastasis)		3	NA	NA	NA	NA	NA	Trunk
MALE	Primary Tumor	NA		V	YES	T4b	N3	M1c	Trunk
MALE	Regional Lymph Node		7	V	NO	NA	NA	NA	Extremities
MALE	Regional Lymph Node	NA		NA	NA	NA	NA	NA	NA
MALE	Regional Cutaneous or Subcutaneous Tissue (includes satellite and in-transit metastasis)	NA		NA	NA	NA	NA	NA	NA
MALE	Regional Lymph Node		5.2	IV	NO	T4a	N1a	M0	Trunk
FEMALE	Regional Lymph Node		9.8	V	NO	T4a	N0	M0	Extremities
MALE	Primary Tumor		5	V	YES	T4b	N3	M0	Trunk
FEMALE	Regional Cutaneous or Subcutaneous Tissue (includes satellite and in-transit metastasis)		2.3	IV	NO	T3a	N1a	M0	Trunk
MALE	Primary Tumor		14	IV	YES	T4b	N2b	M0	Trunk
FEMALE	Regional Lymph Node		12	V	YES	T4b	N0	NA	Extremities
MALE	Distant Metastasis		3	IV	NO	T3a	N1a	M0	Extremities
MALE	Regional Lymph Node		1.7	IV	YES	T2b	NX	M0	Trunk
FEMALE	Primary Tumor		1.15	IV	NO	T2a	NX	M0	Trunk
FEMALE	Regional Lymph Node		1.39	IV	NO	T2b	N3	M0	Extremities
MALE	Regional Lymph Node	NA		NA	NA	NA	NA	NA	Trunk
MALE	Regional Lymph Node		1.7	IV	YES	T2b	N3	M0	Extremities
MALE	Distant Metastasis		0.7	IV	NO	T1b	NX	NA	Head and Neck

FEMALE	Regional Lymph Node	5	NA	YES	T4b	NX	M0	Extremities
MALE	Primary Tumor	4.85	IV	YES	T4b	NX	M0	Trunk
FEMALE	Regional Lymph Node	0.5	III	NO	T1a	NX	M0	Extremities
FEMALE	Distant Metastasis	NA	NA	NA	NA	NA	NA	Extremities
FEMALE	Distant Metastasis	3.4	NA	YES	T3b	N2a	M0	Extremities
FEMALE	Primary Tumor	8	V	NA	T4	NX	M0	Trunk
MALE	Primary Tumor	NA	NA	YES	T4b	N0	M0	Head and Neck
FEMALE	Distant Metastasis	NA	III	NA	NA	NA	NA	Trunk
MALE	Distant Metastasis	NA	NA	NA	TX	NX	M0	Trunk
MALE	Regional Lymph Node	NA	NA	NA	T0	N3	M0	NA
MALE	Regional Lymph Node	NA	NA	NA	TX	N3	M0	NA
FEMALE	Distant Metastasis	NA	NA	NA	NA	NA	NA	NA
MALE	Distant Metastasis	NA	NA	NA	TX	NX	M1c	NA
MALE	Distant Metastasis	NA	NA	NA	TX	NX	M1c	NA
MALE	Distant Metastasis	NA	NA	NA	TX	NX	M1c	Head and Neck
FEMALE	Distant Metastasis	NA	NA	NA	NA	NA	NA	NA
MALE	Regional Lymph Node	1.5	NA	NA	T2	N0	M0	Trunk
FEMALE	Distant Metastasis	1.3	IV	NA	T3	N0	M0	Extremities
MALE	Distant Metastasis	0.5	IV	NA	T3	N0	M0	Head and Neck
MALE	Regional Lymph Node	NA	NA	NA	TX	N2	M0	NA
MALE	Distant Metastasis	NA	NA	NA	TX	N1	M0	NA
MALE	Regional Lymph Node	NA	V	NA	T3	N0	M0	Head and Neck
MALE	Regional Lymph Node	1.3	IV	NO	T3	N0	M0	Other Specify
MALE	Distant Metastasis	1.54	IV	NO	T3	N0	M0	Trunk
MALE	Regional Lymph Node	1.1	III	NO	T2	N0	M0	Extremities
MALE	Regional Lymph Node	14	NA	YES	T4b	N1a	M0	Extremities
MALE	Regional Cutaneous or Subcutaneous Tissue (includes satellite and in-transit metastasis)	3	NA	YES	T3b	N3	M0	Extremities
MALE	Primary Tumor	12	NA	NA	T4a	NX	M0	Extremities
MALE	Regional Cutaneous or Subcutaneous Tissue (includes satellite and in-transit metastasis)	1.7	III	NO	T2a	N3	M1c	Extremities
MALE	Regional Lymph Node	NA	NA	NA	TX	N1b	M0	Trunk

MALE	Regional Cutaneous or Subcutaneous Tissue (includes satellite and in-transit metastasis)	NA	NA	NA	TX	N1b	M0	Extremities
MALE	Regional Cutaneous or Subcutaneous Tissue (includes satellite and in-transit metastasis)	4.5	NA	NO	T4a	NX	M0	Extremities
FEMALE	Regional Lymph Node	2.4	IV	NO	T3a	N1b	M0	Extremities
MALE	Regional Lymph Node	2.3	NA	NO	T3a	N0	M0	Extremities
MALE	Regional Lymph Node	7.3	NA	YES	T4b	N1b	M0	Trunk
FEMALE	Regional Lymph Node	4	NA	YES	T3b	N1b	M1b	Trunk
MALE	Regional Lymph Node	NA	NA	NA	TX	N1b	M0	NA
MALE	Regional Cutaneous or Subcutaneous Tissue (includes satellite and in-transit metastasis)	3	NA	NO	T3a	NX	M0	Trunk
MALE	Regional Lymph Node	1.4	NA	NO	T2a	N3	M0	Extremities
MALE	Regional Lymph Node	0.8	NA	NO	T1a	N0	M0	Head and Neck
FEMALE	Regional Lymph Node	7	NA	YES	T4b	N2b	M0	Extremities
FEMALE	Regional Cutaneous or Subcutaneous Tissue (includes satellite and in-transit metastasis)	NA	NA	NA	TX	N3	M0	Extremities
FEMALE	Primary Tumor	5	IV	NA	T4	N0	M0	Other Specify
MALE	Primary Tumor	1	II	NA	T1	N0	M0	Extremities
MALE	Primary Tumor	2	IV	NA	T2	N0	M0	Extremities
FEMALE	Primary Tumor	10	IV	NA	T4	N0	M0	Trunk
FEMALE	Primary Tumor	13	V	NA	T4	N0	M0	Extremities
FEMALE	Primary Tumor	16	V	NA	T4	N3	M0	Trunk
FEMALE	Regional Lymph Node	NA	NA	NA	T3	NX	M0	Head and Neck
FEMALE	Distant Metastasis	1	IV	NO	T1a	NX	M0	Extremities
MALE	Distant Metastasis	1.5	III	NA	NA	NA	NA	Head and Neck
FEMALE	Distant Metastasis	1.5	IV	NO	NA	NA	NA	Head and Neck
MALE	Primary Tumor	17	IV	NA	T4b	N0	M0	Trunk
MALE	Regional Lymph Node	NA	NA	NA	NA	NA	NA	Trunk
FEMALE	Distant Metastasis	10	V	NO	T4a	N0	M0	Extremities
FEMALE	Regional Cutaneous or Subcutaneous Tissue (includes satellite and in-transit metastasis)	4	III	YES	T3b	N0	M0	Extremities
MALE	Regional Lymph Node	0.5	II	NO	T1a	N0	M0	Trunk

FEMALE	Regional Lymph Node	0.5	NA	NO	T1a	N0	M0	Trunk
