# Supplementary material for: Distinct genomic features across cytolytic subgroups in skin melanoma
Source: Cancer Immunol Immunother. 2021 Mar 29;70(11):3137–54. doi: 10.1007/s00262-021-02918-3 (PMC8505325; doi:10.1007/s00262-021-02918-3)
Supplement: Supplementary file 11 — Supplementary file11 (PDF 64 kb) [file 262_2021_2918_MOESM11_ESM.pdf]

Top 50 DEGs in Primary SKCM

rownames.fit.cont.	gene_name	logFC	AveExpr	t	P.Value	adj.P.Val	B
ENSG00000153563.14	CD8A	6.243667	0.995724	27.19791	5.54E-33	1.07E-28	64.36526
ENSG00000180644.6	PRF1	5.299835	1.13931	26.75623	1.25E-32	1.21E-28	63.61611
ENSG00000105374.8	NKG7	5.809775	1.021256	25.1716	2.59E-31	1.67E-27	60.71411
ENSG00000101082.12	SLA2	4.83996	-0.83275	24.8381	5.00E-31	2.07E-27	59.38819
ENSG00000089692.7	LAG3	5.760131	0.971241	24.80307	5.35E-31	2.07E-27	60.00903
ENSG00000188389.9	PDCD1	6.0598	-0.94789	24.31333	1.43E-30	4.60E-27	58.50531
ENSG00000005844.16	ITGAL	4.640358	2.014015	24.1146	2.14E-30	5.90E-27	58.81917
ENSG00000277734.3	TRAC	4.780387	2.240318	23.79937	4.07E-30	9.84E-27	58.22006
ENSG00000181847.10	TIGIT	5.214556	-0.00129	23.41899	8.94E-30	1.78E-26	57.07392
ENSG00000154451.13	GBP5	5.334773	1.47647	23.40465	9.21E-30	1.78E-26	57.34788
ENSG00000116824.4	CD2	4.994926	1.026293	22.21061	1.17E-28	2.06E-25	54.82477
ENSG00000049249.7	TNFRSF9	5.078757	-0.68347	21.98684	1.91E-28	2.85E-25	53.93718
ENSG00000198821.9	CD247	4.288519	-0.07737	21.985	1.92E-28	2.85E-25	54.0649
ENSG00000139193.3	CD27	4.888322	0.023724	21.83639	2.66E-28	3.67E-25	53.83326
ENSG00000137078.7	SIT1	4.910188	-1.13351	21.62766	4.22E-28	5.44E-25	53.00398
ENSG00000172116.20	CD8B	6.224656	-1.26076	21.49941	5.62E-28	6.79E-25	52.81993
ENSG00000160791.13	CCR5	4.349069	0.744498	21.13448	1.28E-27	1.45E-24	52.45256
ENSG00000145649.7	GZMA	4.990869	0.061184	20.88174	2.27E-27	2.44E-24	51.79389
ENSG00000089012.13	SIRPG	5.686322	-1.28433	20.30948	8.50E-27	8.66E-24	50.2077
ENSG00000167286.8	CD3D	4.95175	0.084085	20.15467	1.22E-26	1.18E-23	50.1763
ENSG00000182866.15	LCK	4.777145	0.638055	19.92029	2.12E-26	1.96E-23	49.72693
ENSG00000161405.15	IKZF3	5.2559	1.016038	19.86329	2.43E-26	2.14E-23	49.64763
ENSG00000162739.12	SLAMF6	5.245601	-0.93121	19.81963	2.70E-26	2.27E-23	49.18617
ENSG00000198851.8	CD3E	4.98766	1.570627	19.34949	8.34E-26	6.73E-23	48.48247
ENSG00000152969.15	JAKMIP1	5.190261	-2.66155	19.21076	1.17E-25	9.04E-23	47.2146
ENSG00000160185.12	UBASH3A	4.746446	-1.69972	19.02017	1.86E-25	1.39E-22	47.07182
ENSG00000102879.14	CORO1A	3.725987	3.708418	18.94293	2.25E-25	1.61E-22	47.57292
ENSG00000275302.1	CCL4	4.439561	0.786384	18.91854	2.39E-25	1.65E-22	47.38134
ENSG00000172215.5	CXCR6	4.242653	-0.16062	18.74493	3.68E-25	2.45E-22	46.80628
ENSG00000211772.7	TRBC2	5.001347	1.687483	18.7063	4.05E-25	2.61E-22	46.93963
ENSG00000073861.2	TBX21	4.512241	-1.84747	18.66518	4.48E-25	2.80E-22	46.17922
ENSG00000186810.7	CXCR3	5.228591	-0.3239	18.58629	5.45E-25	3.30E-22	46.4464
ENSG00000271503.4	CCL5	4.967192	3.379208	18.44508	7.77E-25	4.34E-22	46.341
ENSG00000123329.16	ARHGAP9	3.520994	1.477701	18.44215	7.83E-25	4.34E-22	46.26089
ENSG00000100450.11	GZMH	5.310887	-0.57899	18.44122	7.84E-25	4.34E-22	46.05093
ENSG00000173762.6	CD7	5.153592	0.646401	18.38021	9.15E-25	4.91E-22	46.07253
ENSG00000185905.3	C16orf54	4.024689	-0.73315	18.33958	1.01E-24	5.26E-22	45.68544
ENSG00000185811.15	IKZF1	3.689346	1.197146	18.33151	1.03E-24	5.26E-22	45.96961
ENSG00000147168.11	IL2RG	4.188071	2.344002	18.18706	1.49E-24	7.22E-22	45.67716
ENSG00000111537.4	IFNG	5.914525	-3.88148	18.18639	1.49E-24	7.22E-22	44.47318
ENSG00000155926.12	SLA	3.461915	1.899949	17.94365	2.77E-24	1.31E-21	45.04377
ENSG00000124256.13	ZBP1	4.803576	-1.29471	17.90129	3.09E-24	1.41E-21	44.53629

ENSG00000110448.9	CD5	4.109148	0.081908	17.89711	3.12E-24	1.41E-21	44.77657
ENSG00000107742.11	SPOCK2	3.386949	2.711187	17.7872	4.14E-24	1.82E-21	44.67333
ENSG00000110324.8	IL10RA	3.301621	2.709367	17.7429	4.65E-24	2.00E-21	44.55997
ENSG00000172543.6	CTSW	4.740676	0.508798	17.66872	5.63E-24	2.37E-21	44.27788
ENSG00000125347.12	IRF1	3.805249	4.290253	17.38985	1.16E-23	4.79E-21	43.65644
ENSG00000105122.11	RASAL3	3.536191	1.389763	17.35603	1.27E-23	5.13E-21	43.51964
ENSG00000122122.9	SASH3	3.519849	1.928626	17.34146	1.32E-23	5.22E-21	43.50682
ENSG00000135077.7	HAVCR2	3.324741	1.76274	17.31381	1.42E-23	5.50E-21	43.42655

Top 50 DEGs in Metastatic SKCM

rownames.fit.cont.	gene_name	logFC	AveExpr	t	P.Value	adj.P.Val	B
ENSG00000125347.12	IRF1	3.79973	5.240043	29.35768	1.27E-70	2.76E-66	150.6093
ENSG00000019582.13	CD74	4.292487	9.947889	27.64676	9.08E-67	9.84E-63	141.7068
ENSG00000117560.7	FASLG	5.562351	-0.59938	26.33774	1.02E-63	7.35E-60	134.2037
ENSG00000152969.15	JAKMIP1	5.640063	-0.86795	25.90407	1.09E-62	5.45E-59	131.8215
ENSG00000101082.12	SLA2	4.880802	0.735137	25.87821	1.26E-62	5.45E-59	131.991
ENSG00000173372.15	C1QA	4.03581	6.701105	25.59481	6.01E-62	2.05E-58	130.6395
ENSG00000111537.4	IFNG	6.637651	-1.98054	25.57719	6.62E-62	2.05E-58	129.7783
ENSG00000050730.14	TNIP3	4.975161	-1.97453	25.13254	7.86E-61	2.13E-57	127.4545
ENSG00000145649.7	GZMA	5.544264	1.721416	24.9575	2.10E-60	5.05E-57	127.0268
ENSG00000173369.14	C1QB	4.234297	6.839926	24.91819	2.62E-60	5.67E-57	126.8666
ENSG00000180644.6	PRF1	5.464134	3.100662	24.90046	2.89E-60	5.69E-57	126.7974
ENSG00000128284.18	APOL3	4.136586	3.939916	24.82101	4.52E-60	8.16E-57	126.3829
ENSG00000159189.10	C1QC	3.892286	6.750542	24.72213	7.89E-60	1.27E-56	125.76
ENSG00000154451.13	GBP5	5.934884	3.520192	24.71523	8.20E-60	1.27E-56	125.7658
ENSG00000105374.8	NKG7	5.733726	2.85556	24.53606	2.26E-59	3.26E-56	124.7389
ENSG00000271503.4	CCL5	5.153811	4.673677	24.41332	4.53E-59	6.14E-56	124.0849
ENSG00000211694.2	TRGV10	4.990289	-3.01619	24.39278	5.09E-59	6.49E-56	123.1659
ENSG00000116824.4	CD2	5.43008	2.708786	24.30728	8.29E-59	9.97E-56	123.4475
ENSG00000109943.7	CRTAM	4.727746	-0.22252	24.22036	1.36E-58	1.55E-55	122.6888
ENSG00000153563.14	CD8A	6.04462	3.166377	24.16773	1.84E-58	1.99E-55	122.6628
ENSG00000204287.12	HLA-DRA	4.392668	8.160863	24.06761	3.26E-58	3.36E-55	122.0158
ENSG00000223865.9	HLA-DPB1	3.691139	7.043921	24.0187	4.31E-58	4.24E-55	121.749
ENSG00000147168.11	IL2RG	4.753812	3.918856	23.9269	7.30E-58	6.87E-55	121.3154
ENSG00000235576.1	AC092580.4	5.2261	-2.43567	23.85205	1.12E-57	9.89E-55	120.2495
ENSG00000181847.10	TIGIT	5.725539	1.920839	23.84909	1.14E-57	9.89E-55	120.7939
ENSG00000073861.2	TBX21	4.622299	-0.1663	23.80008	1.51E-57	1.26E-54	120.3257
ENSG00000138755.5	CXCL9	6.299249	4.965972	23.76635	1.84E-57	1.47E-54	120.3955
ENSG00000102879.14	CORO1A	4.08129	5.13488	23.73601	2.19E-57	1.66E-54	120.1982
ENSG00000160185.12	UBASH3A	5.02775	0.011679	23.73328	2.22E-57	1.66E-54	119.9559
ENSG00000049249.7	TNFRSF9	5.182226	1.248737	23.7163	2.45E-57	1.77E-54	119.9972
ENSG00000089012.13	SIRPG	5.913086	0.751219	23.70081	2.68E-57	1.87E-54	119.8309

ENSG00000172215.5	CXCR6	4.860593	1.38135	23.55134	6.36E-57	4.31E-54	119.0728
ENSG00000256262.1	USP30-AS1	4.28117	-1.43297	23.34894	2.06E-56	1.35E-53	117.5968
ENSG00000182866.15	LCK	5.164384	2.418153	23.21693	4.44E-56	2.83E-53	117.1961
ENSG00000188389.9	PDCD1	5.952449	0.960637	23.11564	8.03E-56	4.83E-53	116.4906
ENSG00000183918.13	SH2D1A	5.492679	0.46021	23.11277	8.16E-56	4.83E-53	116.4362
ENSG00000198821.9	CD247	4.638174	1.611434	23.10753	8.42E-56	4.83E-53	116.5331
ENSG00000160654.8	CD3G	5.334625	0.829472	23.1063	8.48E-56	4.83E-53	116.4437
ENSG00000198851.8	CD3E	5.362097	3.292399	23.07536	1.02E-55	5.64E-53	116.3924
ENSG00000277734.3	TRAC	5.026759	3.924795	23.03894	1.26E-55	6.81E-53	116.1852
ENSG00000188820.11	FAM26F	4.584848	2.187836	22.96082	1.99E-55	1.05E-52	115.7069
ENSG00000172673.9	THEMIS	5.304165	-0.71893	22.91424	2.61E-55	1.35E-52	115.1572
ENSG00000005844.16	ITGAL	4.800892	3.864086	22.90456	2.76E-55	1.39E-52	115.4001
ENSG00000167286.8	CD3D	5.49325	1.612466	22.79869	5.15E-55	2.54E-52	114.7179
ENSG00000167208.13	SNX20	4.172249	2.012245	22.72701	7.86E-55	3.78E-52	114.3396
ENSG00000211689.5	TRGC1	4.709314	-2.04976	22.7196	8.21E-55	3.86E-52	113.874
ENSG00000160791.13	CCR5	4.629127	2.325572	22.68826	9.87E-55	4.55E-52	114.1171
ENSG00000089692.7	LAG3	5.661492	2.718381	22.63036	1.39E-54	6.27E-52	113.7766
ENSG00000133574.8	GIMAP4	3.38786	3.946259	22.5162	2.73E-54	1.21E-51	113.108
ENSG00000137078.7	SIT1	4.954163	0.715821	22.37449	6.32E-54	2.74E-51	112.1756
