# Supplementary material for: Distinct genomic features across cytolytic subgroups in skin melanoma
Source: Cancer Immunol Immunother. 2021 Mar 29;70(11):3137–54. doi: 10.1007/s00262-021-02918-3 (PMC8505325; doi:10.1007/s00262-021-02918-3)
Supplement: Supplementary file 12 — Supplementary file12 (PDF 55 kb) [file 262_2021_2918_MOESM12_ESM.pdf]

CYT-high SKCM		CYT-low SKCM	
Gain	Loss	Gain	Loss
KRAS	ETV6	LIFR	IL7R
WIF1	CDK4	KDM5A	IL6ST
MDM2	DDIT3	ZNF384	ARID2
CLTCL1	ALDH2	DDIT3	ATF1
BCR	PTPN11	NACA	HOXC11
SMARCB1	RBM15	CDK4	HOXC13
MN1	HIST1H4I	LRIG3	HMGA2
MYH9	CCND3	LRIG3	WIF1
IL7R	TFEB	MDM2	ETV6
LIFR	HNRNPA2I	MDM2	KRAS
ARID2	HOXA11	FGFR3	ARID2
HOXC11	HOXA13	WHSC1	ATF1
HOXC13	HOXA9	SLC34A2	CDK4
NACA	JAZF1	IL7R	DDIT3
CDK4	MLF1	MDM2	HMGA2
DDIT3	SOX2	AKT1	HOXC11
LRIG3	ETV5	CEBPA	HOXC13
ALDH2	FANCF	AKT2	LRIG3
ALDH2	WT1	CD79A	NACA
PTPN11	LMO2	CIC	WIF1
BCL7A	NTRK3	MLLT10	TSHR
NRAS	H3F3A	KIF5B	DICER1
TRIM33	BTG1	GNAS	GOLGA5
TRIM27	IL6ST	NFIB	TCL1A
POU5F1	IL7R	NFIB	TRIP11
DAXX	LIFR	MLLT3	BCL11B
FANCE	PIK3R1	FANCG	PCM1
HMGA1	APC	PAX5	CD274
PIM1	CD74	NIN	JAK2
CARD11	PDGFRB	TSHR	KTN1
CARD11	FUBP1	TRIP11	GPHN
PMS2	BCL10	DICER1	PRDM1
ETV1	RBM15	GOLGA5	GOPC
ETV1	CLTCL1	TRIP11	ROS1
GATA2	CHEK2	TCL1A	POU5F1
RPN1	MEN1	BCL11B	DAXX
FOXL2	PML	HIST1H4I	FANCE
CDK4	IDH2	TRIM27	HMGA1
DDIT3	BCL10	POU5F1	PIM1
MDM2	FUBP1	CCND3	CCND3
ALDH2	RBM15	DAXX	TFEB
PTPN11	FAM46C	FANCE	TMPRSS2
GMPS	NOTCH2	HMGA1	U2AF1
PIK3CA	NRAS	PIM1	PRDM1
BCL6	TRIM33	TFEB	GOPC
EIF4A2	PDE4DIP	HIST1H4I	ROS1
LPP	BCL9	OLIG2	CCND2
LPP	ARNT	RUNX1	ZNF384

RAP1GDS1	NTRK1	ERG	ETV6
PML	PRCC	PML	CAMTA1
BLM	FCGR2B	NTRK3	PRDM16
CRTC3	PBX1	BLM	RPL22
IDH2	SDHC	CRTC3	TNFRSF14
BCR	H3F3A	IDH2	MDS2
SMARCB1	FH	ETV6	PAX7
CHEK2	ERCC3	KRAS	SDHB
EWSR1	SLC34A2	FAM46C	ARID1A
MN1	FIP1L1	BCL9	LCK
NF2	BUB1B	NOTCH2	SFPQ
MYH9	TCF12	PDE4DIP	THRAP3
MYH9	GAS7	ARNT	CDKN2C
EP300	PER1	MUC1	EPS15
MKL1	TP53	NTRK1	MPL
PDGFB	MAP2K4	PRCC	MUTYH
NFIB	PRDM1	TPM3	TAL1
MLLT3	MYB	SDHC	JUN
NRAS	TNFAIP3	FCGR2B	JAK1
RBM15	ECT2L	PBX1	FUBP1
TRIM33	FGFR1OP	PBX1	BCL10
FAM46C	MLLT4	ABL2	RBM15
FAM46C	CHEK2	TPR	NRAS
NOTCH2	EWSR1	ELK4	TRIM33
PDE4DIP	MN1	MDM4	MAML2
PDE4DIP	NF2	SLC45A3	MLH1
BCL9	MKL1	H3F3A	CTNNB1
ARNT		FH	SETD2
BCR		PICALM	BAP1
SMARCB1		MYD88	PBRM1
MN1		FOXP1	FHIT
EP300		MITF	PCM1
MKL1		TCF12	WRN
NOTCH2		PML	FGFR1
PDE4DIP		PML	WHSC1L1
PDE4DIP		NTRK3	BUB1B
CCND1		BLM	PALB2
ARNT		CRTC3	FUS
TPM3		IDH2	CYLD
MUC1		IL21R	CHEK2
NTRK1		CLTCL1	EWSR1
PRCC		BCR	NF2
FCGR2B		BCR	CREB1
PBX1		SMARCB1	IDH1
SDHC		MN1	ACSL3
ABL2		NF2	ATIC
TPR		MYH9	FEV
ELK4		PDGFB	PAX3
MDM4		EP300	BCR
SLC45A3		MKL1	SMARCB1

H3F3A	IL7R	CHEK2
FH	LIFR	EWSR1
NTRK3	PMS2	MN1
PRDM16	ETV1	NF2
TNFRSF14	HNRNPA2E	ARID2
CAMTA1	HOXA11	ATF1
RPL22	HOXA13	HOXC11
ARID1A	HOXA9	ASPSCR1
MDS2	JAZF1	MAFB
PAX7	ASXL1	RBM15
SDHB	MAFB	FAM46C
PDE4DIP	TOP1	LMO2
BCL9	CLTCL1	WT1
MUC1	MYH9	CREB3L1
TPM3	PDGFB	DDB2
ELK4	EP300	EXT2
MDM4	MKL1	MEN1
SLC45A3	EP300	PICALM
FGFR3	HIST1H4I	MAML2
WHSC1	TRIM27	ATM
EP300	POU5F1	DDX10
FH	DAXX	POU2AF1
TTL	FANCE	SDHD
TTL	HMGA1	ATF1
PHOX2B	PIM1	HOXC11
CHIC2	CCND3	HOXC13
CHIC2	TFEB	GMPS
PDGFRA	HOXC13	MET
FIP1L1	NACA	CREB3L2
PDGFRA	DDIT3	BRAF
KIT	CDK4	DDB2
KDR	LRIG3	MEN1
HIST1H4I	HMGA2	ATM
TRIM27	WIF1	DDX10
POU5F1	MDM2	POU2AF1
DAXX	BRIP1	SDHD
FANCE	CD79B	PAFAH1B2
HMGA1	DDX5	PCSK7
CCND3	CANT1	WHSC1
TFEB	MITF	JAK2
PRDM1	MITF	CD274
GOPC	FOXP1	NFIB
ROS1	FOXP1	NFIB
YWHAE	GNAS	PAX5
USP6	SS18L1	CHEK2
BCR	LHFP	EWSR1
BCR	LCP1	MN1
GOPC	RB1	NF2
ROS1	ERCC5	MYH9
EZR	NRAS	ARID2

BCR	TRIM33	ATF1
MYH9	ARNT	CDK4
EP300	BCL9	DDIT3
ABL2	NOTCH2	HMGA2
ELK4	PDE4DIP	HOXC11
MDM4	FCGR2B	HOXC13
SLC45A3	PBX1	LRIG3
H3F3A	TPR	NACA
	FANCF	WIF1
	CCND1	HOOK3
	MEN1	TCEA1
	NUMA1	PICALM
	LIFR	MAML2
	LIFR	BUB1B
	CCND1	TPR
	NUMA1	H3F3A
	BIRC3	FANCC
	NACA	SET
	DDIT3	ABL1
	CDK4	BRD3
	LRIG3	FNBP1
	WIF1	NUP214
	HMGA2	RALGDS
	HMGA2	TSC1
	MDM2	NOTCH1
	BRAF	HNRNPA2B1
	KIAA1549	HOXA11
	LMO1	HOXA13
	LMO2	HOXA9
	PICALM	JAZF1
	FUBP1	WT1
	NOTCH2	CREB3L1
	PDE4DIP	DDB2
	PDE4DIP	EXT2
	BCL9	MEN1
	ARNT	NF1
	MUC1	SUZ12
	TPM3	TAF15
	NTRK1	PRKAR1A
	PRCC	AKT2
	FCGR2B	CD79A
	SDHC	CIC
	PBX1	BCL3
	SLC34A2	CBLC
	MLLT3	ERCC2
	MLLT3	KLK2
	FANCG	PPP2R1A
	PDGFB	SMARCB1
	MKL1	CHEK2
	MKL1	NF2

EP300	MYH9
ETV6	PDGFB
KRAS	MKL1
MDM2	WRN
HIST1H4I	FGFR1
TRIM27	WHSC1L1
POU5F1	ATF1
DAXX	BTG1
FANCE	ALDH2
HMGA1	BCL7A
PIM1	GNAS
CCND3	
TFEB	
FGFR1	
WHSC1L1	
HOOK3	
RECQL4	
BRCA2	
LHFP	
RB1	
TCEA1	
CHCHD7	
PLAG1	
CCND1	
NUMA1	
BIRC3	
TCF12	
PML	
PML	
SS18	
HIST1H4I	
TRIM27	
POU5F1	
DAXX	
FANCE	
HMGA1	
PIM1	
CCND3	
TFEB	
ARNT	
MUC1	
TPM3	
FCGR2B	
SDHC	
ABL2	
EWSR1	
MYH9	
EP300	
FIP1L1	
CHIC2	

CHIC2
PDGFRA
FIP1L1
PDGFRA
KDR
KIT
CARD11
PMS2
PMS2
ETV1
LMO2
CCND1
CDK12
ERBB2
LASP1
MLLT6
RARA
HLF
MSI2
CLTC
BRIP1
BRIP1
CD79B
DDX5
CANT1
CEBPA
AKT2
ZNF331
BCR
MN1
MKL1
EP300
EP300
PCM1
HOOK3
KDM5A
CCND2
ZNF384
ETV6
KRAS
ARID2
ARID2
HOXC11
HOXC13
NACA
CDK4
DDIT3
LRIG3
WIF1
WIF1

HMGA2
MDM2
PTPN11
ASXL1
MAFB
TOP1
SDC4
SS18L1
